# Gradual evolution of a homo-l-peptide world on homo-d-configured RNA and DNA†

**DOI:** 10.1039/d4sc03384a

**Published:** 2024-08-02

**Authors:** Ewa Węgrzyn, Ivana Mejdrová, Thomas Carell

**Affiliations:** a Department of Chemistry, Center for Nucleic Acids Therapies at the Institute for Chemical Epigenetics (ICE-M), Ludwig-Maximilians-Universität (LMU) München Butenandtstrasse 5-13 81377 Munich Germany thomas.carell@lmu.de

## Abstract

Modern life requires the translation of genetic information – encoded by nucleic acids – into proteins, which establishes the essential link between genotype and phenotype. During translation, exclusively l-amino acids are loaded onto transfer RNA molecules (tRNA), which are then connected at the ribosome to give homo-l-proteins. In contrast to the homo-l-configuration of amino acids and proteins, the oligonucleotides involved are all d-configured (deoxy)ribosides. Previously, others and us have shown that if peptide synthesis occurs at homo d-configured oligonucleotides, a pronounced l-amino acid selectivity is observed, which reflects the d-sugar/l-amino acid world that evolved in nature. Here we further explore this astonishing selectivity. We show a peptide-synthesis/recapture-cycle that can lead to a gradual enrichment and hence selection of a homo-l-peptide world. We show that even if peptides with a mixed l/d-stereochemistry are formed, they are not competitive against the homo-l-counterparts. We also demonstrate that this selectivity is not limited to RNA but that peptide synthesis on DNA features the same l-amino acid preference. In total, the data bring us a step closer to an understanding of how homochirality on Earth once evolved.

## Abstract 2

Nucleic acids with hypermodified nucleosides perform stereoselective RNA/DNA-mediated peptide coupling, followed by thermal release of the peptide, leading to temperature-driven one-pot cycles with gradual homochiral l-peptide enrichment.

## Graphical abstract

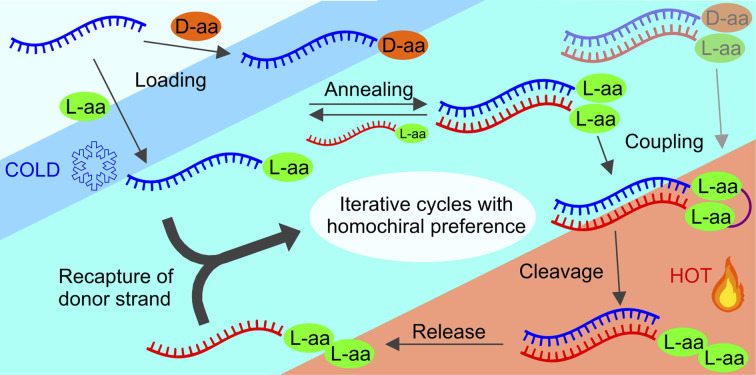

## Introduction

Life, as we know it, requires nucleic acid biomolecules to encode genetic information and amino acid–based proteins to catalyse biochemical reactions, that are essential for the maintenance of life. While the genetic code contains the blueprint for the multitude of vital proteins, the proteins in turn are essential for metabolism and required for decoding the sequence information and its replication. This strong interdependence of the genotype established by nucleic acids and the phenotype established by proteins is a hallmark of all life on Earth.^1^ The element where the genotype “meets” the phenotype is the ribosome.^2–4^ Consequently, the evolution of the ribosome is a chicken-and-egg conundrum and one of the most challenging mysteries of the origin of life.^5,6^ The translation machinery that is in place today shows an extremely high stereoselectivity towards l-amino acids. First, a specific transfer RNA (tRNA) consisting of d-riboses is selectively loaded with the corresponding l-amino acid.^7^ In the step of translation, these l-amino acids are connected to other l-amino acids in a messenger RNA (mRNA)-based templation process, catalysed by the RNA components (all l-configured) of the ribosome. The exact time-point when homochirality emerged is unknown. Mechanistically it was suggested that it was caused by a small initial enantiomeric imbalance on the monomer level,^8^ followed by processes of chiral amplification,^9,10^ leading to an enantiomeric induction between homochiral nucleic acids and peptides.^11,12^

Numerous ideas exist of how the decoding^13^ could have evolved,^14–16^ which are all connected to the RNA world concept.^17–19^ This model predicts that in prebiotic times it was only RNA which encoded information and which also catalysed the essential reactions such as loading itself with specific amino acids.^20–22^ In the RNA world, the process of connecting amino acids was catalysed by RNA.^22,23^ The fact that the peptidyl transferase center of the ribosome is composed of RNA,^24^ provides strong support for this “RNA-only” idea.^25,26^

Currently, the idea of an RNA-only world is questioned. Recent studies^27^ suggest instead the coexistence/coevolution of RNA with other entities like peptides,^28^ cofactors^29,30^ or DNA.^31–33^ Even a DNA-first theory was formulated.^33,34^ The fact that most known RNA catalysts have rather low turn-over numbers strongly suggests that amino acids, peptides and cofactors might have been early on involved.^35^ Already in 1976, White pointed out that cofactors alone could be considered as remnants of early life ribozymes,^29,30^ which acted in concert with sulfur-containing modified nucleosides.^36^ Recently the idea of an RNA-peptide world was brought forward,^37,38^ with the peptides adding stability and catalytic capabilities to the RNA.^39^ Such an early partnership could have been the foundation for the development of early amino acid-tRNA synthetases.^40^

**Fig. 1:**
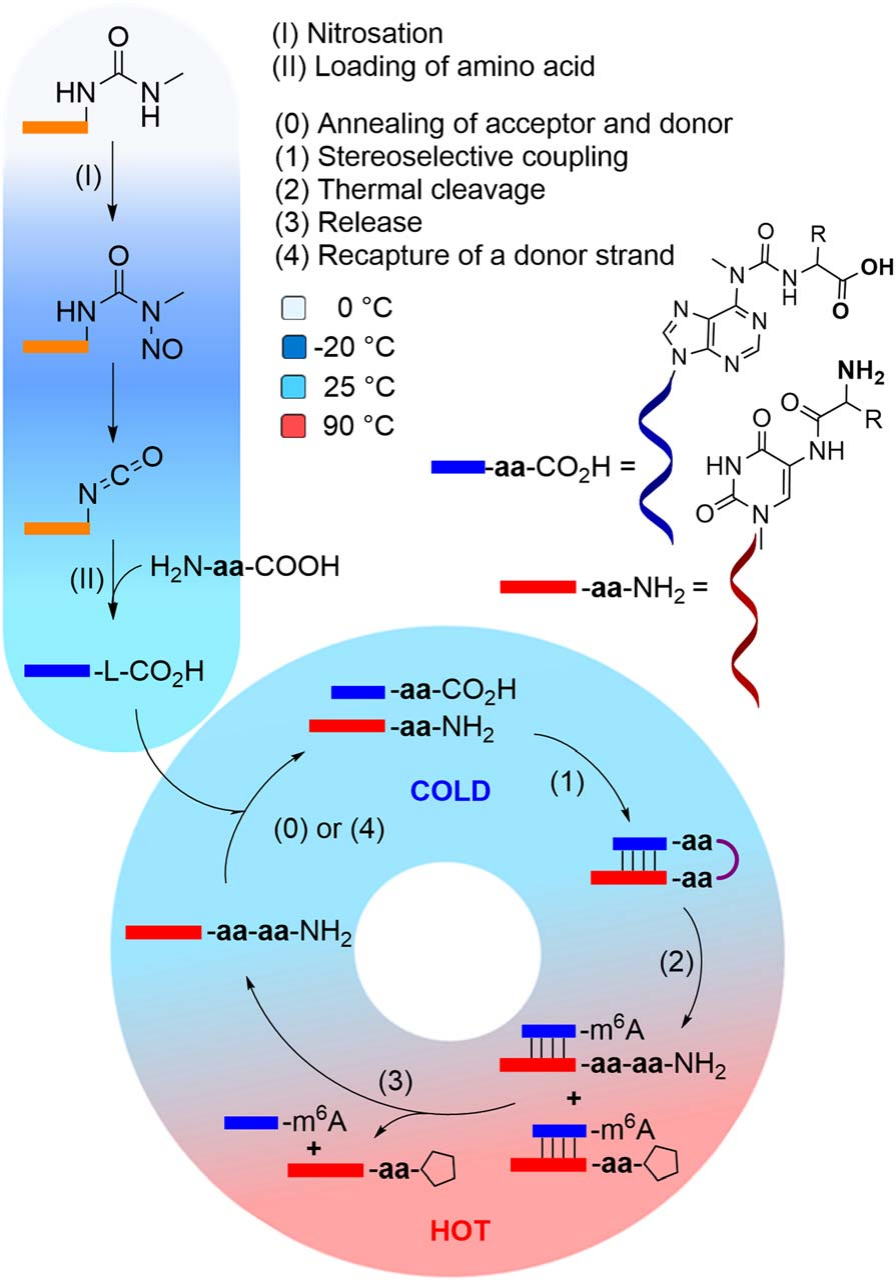
Cold/hot cycle of amino acid loading onto RNA (steps I and II) and iterative peptide synthesis cycle, that could lead to homochiral peptide formation in an outgrowth process. The steps of the cycle include: annealing of the complementary acceptor and donor strands (step 0), stereoselective peptide coupling (step 1), thermal cleavage of the peptide from the donor strand (step 2), followed by a release of the peptide-RNA acceptor conjugate (step 3), that can then recapture another amino acid-loaded donor strand (step 4), and enter another cycle. R stands for l- or d-Val.

We recently introduced the idea, that the high number of non-canonical nucleosides present in RNA, are living fossils^41,42^ of an early RNA/DNA world.^43^ We could show that these non-canonical nucleosides add functions to RNA that would strongly benefit the evolution of life.^44^ For example, non-canonical nucleosides t^6^A, g^6^A, as well as (m)nm^5^U,^45–47^ enable RNA to grow peptides on itself in a process which can establish an RNA-peptide world (Fig. 1).^48^ The loading with amino acids and peptides and their connection to more complex structures is in principle possible under prebiotically plausible freezing/thawing conditions.^49,50^ It was shown, that this loading reaction exhibits no stereochemical bias, meaning that l- and d-amino acids are loaded onto RNA with no preference.^12,51^ However, when the amino acids are allowed to react with each other to give peptides, a large kinetic advantage was observed for the l-configured amino acids.^11,52–57^

Based on this observation, we can envision a primitive peptide synthesis cycle as depicted in Fig. 1, that is driven by cold/hot cycles (−20 °C to +90 °C). In this model, amino acids (aa) and small peptides react (*via* nitrosation of *N*^6^-urea-A) with RNA (step I and II) to give (m^6^)aa^6^A-containing RNA-donor strands ((m^6^)aa^6^A-RNA),^51^ which can hybridize to complementary RNA acceptor strands containing the non-canonical nucleoside (m)nm^5^U (step 0) in the cold. Upon activation and reaction of the amino acids with the (m)nm^5^U nucleoside (step 1), a hairpin structure with a high thermal stability is formed.^48^ During a phase of increased temperature (90 °C), cleavage of the thermally labile urea bond will occur (step 2) together with an immediate dissociation of the strands (step 3). Upon cooling of the solution, a new donor strand can bind (step 4) to continue the hot/cold-governed synthesis cycle.^58^ In the prebiotic context, these temperature variations could have originated from volcanic eruptions, meteorite impact or even day/night-dependent presence of sunlight.^59,60^ Current research reports on one hand cold, even frozen environments on early Earth,^61,62^ and on the other hot temperatures provided by the hydrothermal vents.^63^

We hypothesise that the initially formed peptides were short and released into the environment upon degradation of the RNA. And such they could have been taken up again by donor strands to continue the process at another RNA “host” strand (step II). Here we show that such a scenario is realistic and we show that homo-l peptides would feature a clear competitive advantage over peptide strands contaminated with d-amino acids.

## Results and discussion

**Fig. 2:**
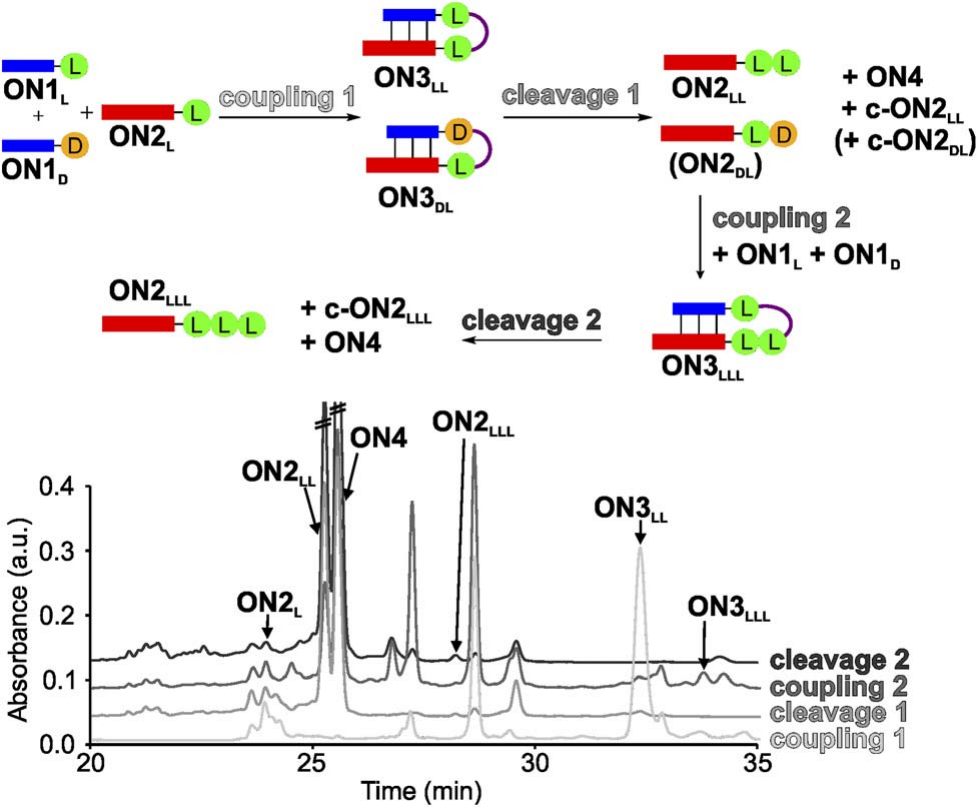
One-pot synthesis cycle scheme and HPLC-chromatograms. For reasons of simplicity, only the products formed in detectable amounts and relevant for further reactions are indicated in the scheme. Yields were determined by HPLC using the calibration curves of reference compounds (Fig. S2 and S3†).

In order to establish a stereochemical self-selecting peptide synthesis cycle, we intended to investigate the one-pot growth of a peptide using a racemic mixture of l- and d-Val RNA donor strands (Fig. 2). We selected valine because it is one of the simplest chiral amino acids for which a prebiotic existence is plausible.^64,65^ Valine has also been observed in meteorites^66,67^ and its formation has been demonstrated in Urey–Miller-type spark discharge experiments.^68–71^

For the experiment, we first synthesized several 7-mer RNA donor strands with either l- or d-Val ON1_L_ (l = l-Val) or ON1_D_ (d = d-Val), or a stereochemically mixed Val-dipeptide, connected to the RNA *via* 5′-*N*^6^-methylcarbamoyl adenosine nucleosides (m^6^-l/d-Val^6^A_m_). As the complementary RNA acceptor strand we prepared an 11-mer RNA strand with a 3′-l- or d-Val-modified 5-methylaminomethyl uridine (mnm^5^U) nucleotide (ON2_L_ or ON2_D_). 2′OMe nucleotides were used for the construction of the RNA to increase the thermal stability.^72,73^ We next combined all the donor ON1_L_ or ON1_D_ with all the acceptor ON2_L_ or ON2_D_ strands in the cold part of the cycle and performed the peptide coupling with EDC/Sulfo-NHS as activator (MES buffer pH 6, NaCl, r.t., 2 h), to obtain the different hairpin products ON3_LL_, ON3_DL_ and ON3_LD_, ON3_DD_ (not shown). In the hot part of the cycle, the hairpin products were cleaved (acetate buffer pH 4, NaCl, 90 °C, 48 h). As the result of the cycle we obtained in all cases the expected dipeptide products ON2_LL_, ON2_DL_ and ON2_LD_, ON2_DD_ (not shown), and the peptide-free m^6^A-RNA ON4, together with a small amount of the corresponding hydantoin by-product c-ON2_LL_, c-ON2_LD_, c-ON2_DL_ or c-ON2_DD_ (see Scheme S6 in ESI†).^48^ All the oligonucleotides formed were purified by high-performance liquid chromatography (HPLC) and characterized using MALDI-ToF mass spectrometry. The experiment showed that the hot/cold cycle is in all cases effective and the obtained products were next used as standards for the analysis of potential diastereoselectivities. It is known from literature, that the measured diastereoselectivity of peptide formation in the absence of the sugar is very low, arguing that it is sugar and the helical chirality that induces the selectivity.^57^

In order to analyse the diastereoselectivities, we next prepared an equimolar solution of 1 eq ON1_L_ and 1 eq ON1_D_ donor strands (always with Val) and added 1 eq of ON2_L_ (Fig. 2 and S4†). The coupling reaction was performed as described above. By using the generated oligonucleotide products from above as references, we determined by HPLC a diastereoselectivity of 94 : 6 in favour of the ll-homochiral product ON3_LL_ over the heterochiral dl-product ON3_DL_ with an overall yield of 58%. After simple filtration of the crude reaction mixture to remove unreacted activator and exchange of the buffer solution, the crude product was introduced into the hot-phase cleavage reaction (48 h, 90 °C). We indeed obtained the cleaved product ON2_LL_ with a diastereoselectivity of 98 : 2 for the ll-dipeptide over the second product ON2_DL_. In addition, the hydantoin by-products c-ON2_LL_ and c-ON2_DL_ were formed. The overall yield was 42% over the two steps.

The reaction mixture was next lyophilized (in analogy to a dry-down step) and the buffer was exchanged (comparable to fresh solution floating in a tidal process). We then added another portion of the equimolar mixture of ON1_L_ and ON1_D_. After activation, we detected the formation of the hairpin product ON3_LLL_ by HPLC and MALDI-ToF, albeit in a low yield of 3% (over three steps). The heterochiral product ON3_LLD_ was not detected. We then went through another hot-phase and performed the cleavage reaction, which provided in 1% yield (over 4 steps) the RNA strand ON2_LLL_ (Fig. 2 and S5; Table S8†). The main reason for the low yield is the large amount of possible side reactions due to a high number of reactants present in the one-pot mixture.^12^

Although we detected a high selectivity for the formation of the ll- and lll-products, we next wanted to learn how the presence of a d-contamination would influence the process. To study this, we synthesized the l,l-Val (ON1_LL_), l,d- (ON1_LD_), d,l- (ON1_DL_) and d,d-Val (ON1_DD_) RNA donors strands and performed the coupling reactions with either the ON2_L_ or the ON2_D_ acceptor strand. Again, in order to enable precise characterization, we first performed the coupling reactions with each donor–acceptor strand combination separately to obtain the tripeptide hairpin products, as reference compounds.

**Fig. 3:**
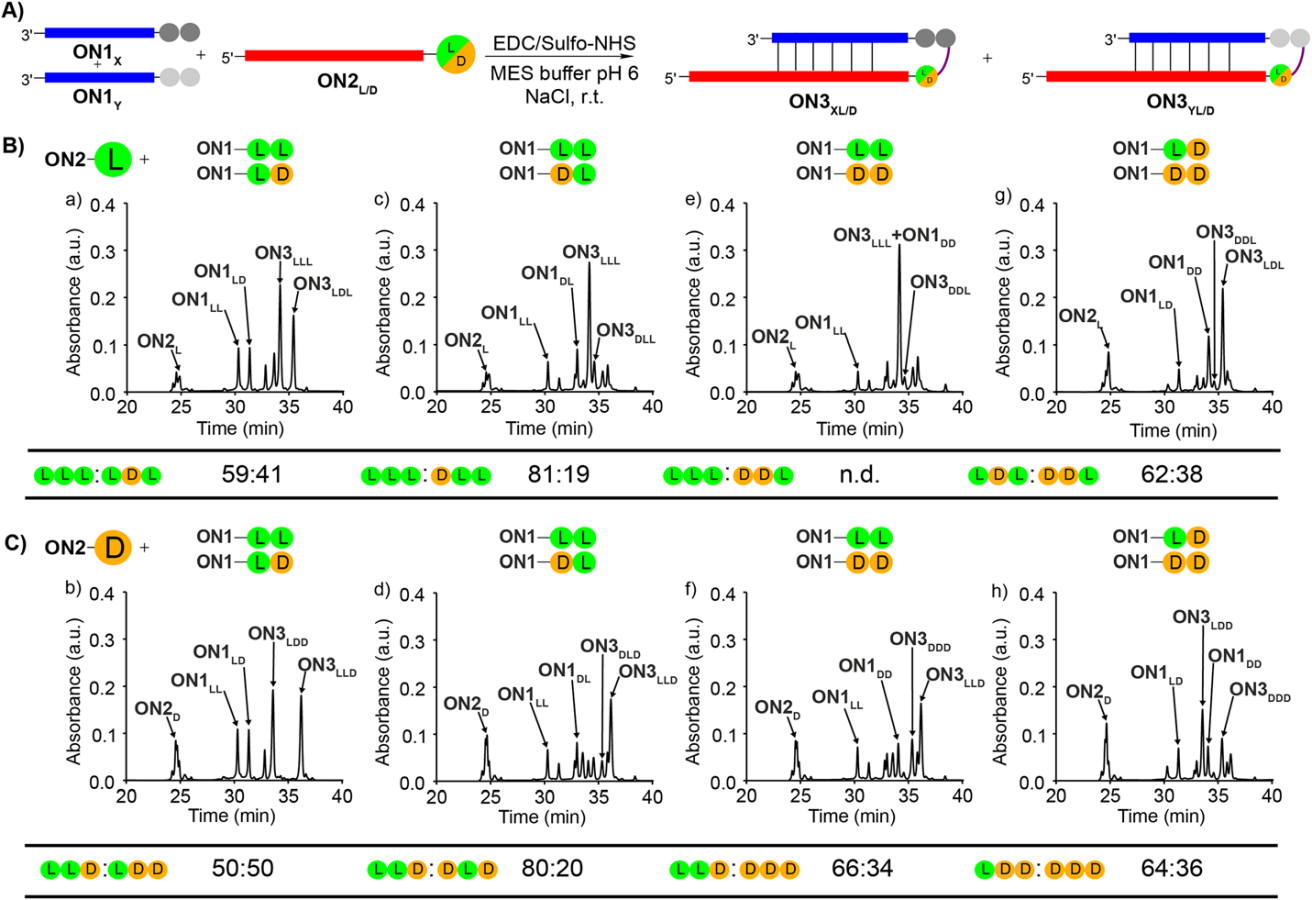
(A) General scheme for the competitive peptide coupling reactions of equimolar mixture of ON1_X_ and ON1_Y_ with (B) ON2_L_ or (C) ON2_D_. X and Y can correspond to either LL, LD, DL or DD, while X ≠ Y. The HPL-chromatograms show the analysed crude reaction mixtures and the tables summarize the results obtained for the competitive coupling reactions, specifically product ratios of (B) [ON3_XL_]/[ON3_YL_] and (C) [ON3_XD_]/[ON3_YD_]. Reaction conditions: [ON1] = 50 μM; [ON2] = 50 μM; [buffer] = 100 mM; [NaCl] = 100 mM and [activator] = 50 mM. Yields were determined by HPLC analysis using the calibration curve of a reference compound (Fig. S2†).

To study the diastereoselectivities (Fig. 3), we next prepared equimolar mixtures of the dipeptide RNA donor strands (referred to as ON1_X_ and ON1_Y_, where X and Y corresponds to either LL, LD, DL or DD, while X ≠ Y).

We allowed the ON1_X_ and ON1_Y_ RNA oligonucleotides with the different dipeptides to compete for reactions with ON2_L_ (Fig. 3A and B) or ON2_D_ (Fig. 3A and C).

First, we allowed the equimolar mixtures of ON1_X_ and ON1_Y_ to react with ON2_L_, using EDC/Sulfo-NHS as the activator (MES buffer pH 6, NaCl, r.t., 2 h). The reaction mixtures were then analysed by HPLC (Fig. 3 and Table S9†). We observed in the reactions yields between 25–56%. Next, we determined the ratio between ON3_XL_ and ON3_YL_. Again, high diastereoselectivities in favor of products that were formed with an l-Val in close proximity of the RNA were observed. The selectivity for ON3_LLL_ over ON3_LDL_ was only 59 : 41 (Fig. 3Ba), but for ON3_LLL_ over ON3_DLD_ we detected 81 : 19 (Fig. 3Bc), suggesting that the exchange of the l-amino acid directly at the nucleobase against the d-counterpart strongly decreases the diastereoselectivity. For the reaction of an equimolar mixture of ON1_LD_ and ON1_DD_ we observed a diastereoselectivity of 62 : 38 for the ON3_LDL_ product over ON3_DDL_ (Fig. 3Bg). Again, the more l-amino acids are present, the better is the peptide formation reaction. The ratios for the other combinations could not be calculated with certainty due to massive peak overlap.

In order to gain deeper insight how a d-amino acids positioned directly at the nucleobase influences the selectivities, we next repeated the competition reactions with ON2_D_ (Fig. 3C and Table S9†). Now, we detected highly fluctuating results, but again, even for ON2_D_ there is some selectivity to react with a donor strand containing a l-Val attached to the RNA-donor strand over a d-Val. We saw a rather limited influence of the second amino acid present in the donor strand. The highest stereoselectivity of 80 : 20 was observed for the products ON3_LLD_*vs.*ON3_DLD_ (Fig. 3Cd). The overall yields were ranging from 24 to 50%.

We noticed during the study that the second amino acid of *e.g.*ON1_LL_ undergoes a *ca.* 25% racemization when incubated under the described coupling conditions for 2 h. The mono-Val donors ON1_L_ and ON1_D_, however, did not racemize when subjected to the same conditions. This phenomenon makes the analysis and interpretation of the described reactions difficult, but the general result is firmly established, that the homo-l situation is always winning.

**Fig. 4:**
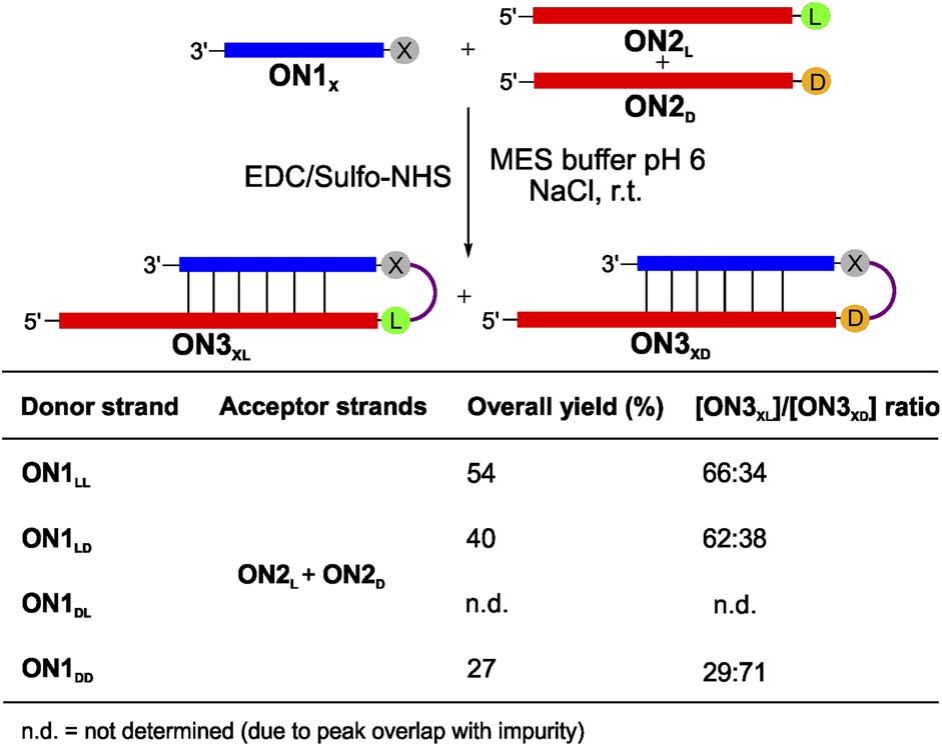
Competitive peptide coupling reactions of equimolar mixture of ON2_L_ and ON2_D_ with ON1_X_. X can correspond to either LL, LD, DL or DD. The table summarizes the results obtained for the competitive coupling reactions. Reaction conditions: [ON1] = 50 μM; [ON2] = 50 μM; [buffer] = 100 mM; [NaCl] = 100 mM and [activator] = 50 mM. Yields were determined by HPLC analysis using the calibration curve of a reference compound (Fig. S2†).

To complete the set of experiments with dipeptide donors, we also analysed the competition reactions of ON1_LL_, ON1_LD_, ON1_DL_ and ON1_DD_ with a 1 : 1 mixture of ON2_L_ and ON2_D_ (Fig. 4 and S7†). For this experiment we prepared an equimolar mixture of ON2_L_ and ON2_D_ and allowed them to react with the dipeptide RNA donor strand ON1_X_ under the described coupling conditions. The reaction with ON1_LL_ showed a selectivity of 66 : 34 in favour of the homochiral lll-hairpin product ON3_LLL_ over the product ON3_LLD_ (Fig. 4). This diastereoselectivity decreased slightly when we used ON1_LD_ (62 : 38, ON3_LDL_ : ON3_LDD_). The d,d-Val donor ON1_DD_ showed a reversed selectivity towards the homochiral all-d product ON3_DDD_ of 71 : 29 compared to ON3_DDL_ (Fig. S7d†). The coupling reaction with ON1_DL_ could not be analysed due to a HPLC peak overlap of the product with an impurity.

**Fig. 5:**
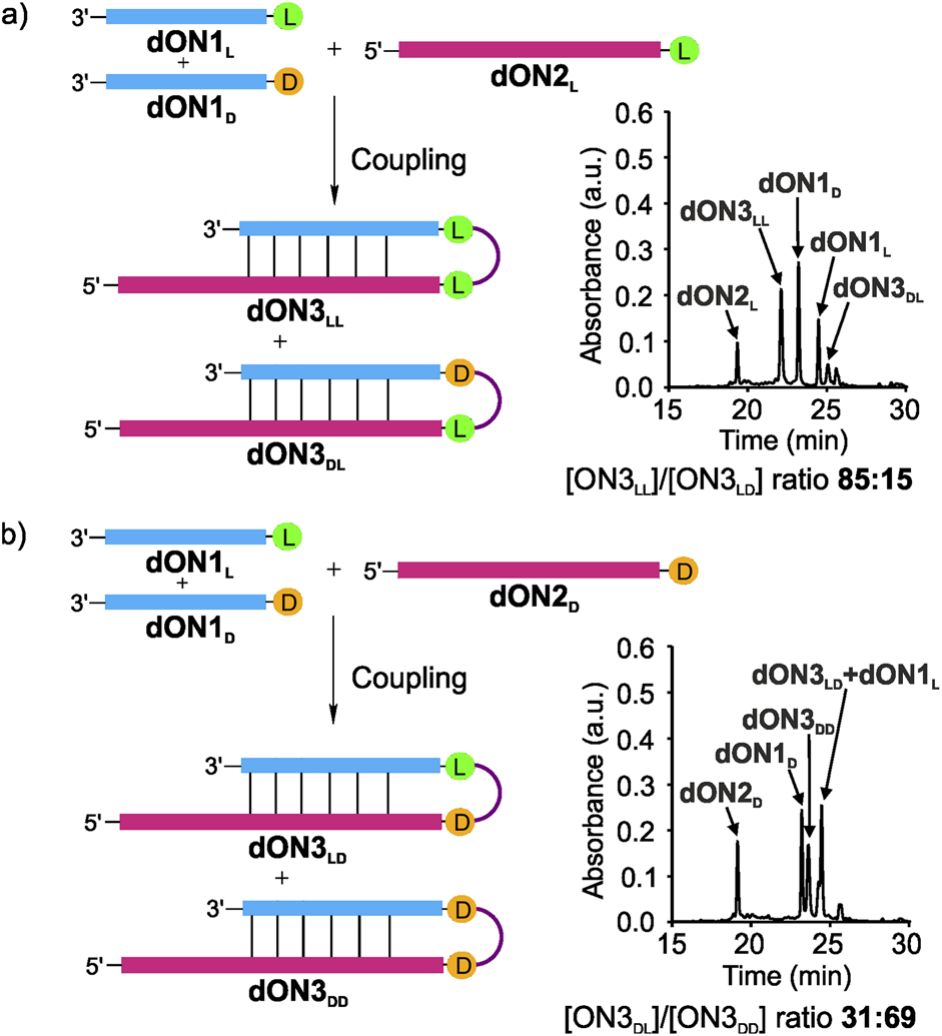
DNA-competitive peptide coupling reactions between dON1_L_ and dON1_D_ with (a) dON2_L_ or (b) dON2_D_ using EDC/Sulfo-NHS as activator. Yields were determined by HPLC analysis using the calibration curve of a reference compound (Fig. S2†).

Finally, we wanted to investigate if the observed tendency to form homo-l products prevails when one moves from RNA to DNA in light of the recent discussion that life may have started with a DNA-world^33^ or a mixed RNA–DNA world.^31^ We therefore synthesized the DNA analogues of the donor strands dON1_L_ and dON1_D_ and the acceptor strands dON2_L_ and dON2_D_ by solid-phase oligonucleotide synthesis to form the hairpin products dON3. We conducted the competitive coupling reactions and observed the formation of the dON3_LL_ with a high diastereoselectivity of 85 : 15 over the heterochiral product dON3_DL_ (Fig. 5a). The reverse competition reaction yielded a 31 : 69 stereoselectivity for dON3_LD_ over dON3_DD_ (Fig. 5b). These results suggest that DNA will induce a similar diastereoselectivity towards the homo-l products.

In order to gain more information about the conformation of the RNA duplexes, we finally measured the circular dichroism (CD) spectra of three different solutions: (1) annealed RNA duplex of ON1_L_ and ON2_L_, (2) hairpin duplex ON3_LL_ and (3) the canonical analogue annealed RNA duplex of ON5 and ON6 (Fig. S9†). We observed CD-spectra typical for double-stranded A-form RNA with no significant changes introduced by the nucleobase modification or the hairpin formation between them.

## Conclusions

In this work we explored the possibility of a primitive temperature-driven one-pot peptide synthesis cycle, in which stereoselective peptide coupling followed by thermal cleavage can lead to an enriched formation, albeit in low yields, of the homochiral all l-peptides.

We investigated how different stereochemical dipeptide combinations, namely the presence of l- and d-amino acids in the cycle, would influence the peptide-forming chemistry. We found that whatever combination we investigated, in almost all cases we observed faster reactions and higher yields with l-amino acids and homo-l-peptides. The l-situation outcompeted reactions with d-amino acids present on the donor or on the acceptor strand, especially when the (l-) amino acid was directly attached to the nucleobase and therefore in close contact with the nucleic acid strand. Important is the observation that this stereochemical “preferential handshake” (d-ribose and l-amino acid) is also true with d-deoxyribose, which forms DNA. It shows us that even in a putative prebiotic world in which DNA-only or a mixed DNA–RNA combination were the carrier of genetic information, the stereochemical preference for l amino acids prevails.

## Author contributions

E. W. and I. M. were responsible for data curation, formal analysis, investigation and visualisation. T. C. had the research idea and was in charge of funding acquisition, project administration and supervision. All authors were involved in the conceptualization and the writing of the manuscript.

## Conflicts of interest

The authors declare no conflicts of interest.

## Supplementary Material

SC-015-D4SC03384A-s001

## Data Availability

The data supporting this article have been included as part of the ESI.†
