# Supplementary material for: Gradual evolution of a homo-l-peptide world on homo-d-configured RNA and DNA
Source: Chem Sci. 2024 Aug 2;15(35):14171–6. doi: 10.1039/d4sc03384a (PMC11306956; doi:10.1039/d4sc03384a)
Supplement: SC-015-D4SC03384A-s001 [file SC-015-D4SC03384A-s001.pdf]

Supplementary Information (SI) for Chemical Science.
This journal is © The Royal Society of Chemistry 2024

Gradual evolution of a homo-L-peptide world on homo-D-configured RNA and DNA

Ewa Węgrzyn,*[a] Ivana Mejdrová,*[a] and Thomas Carell [a]

[a] Department of Chemistry, Center for Nucleic Acids Therapies at the Institute for Chemical Epigenetics (ICE-M), Ludwig-Maximilians-Universität (LMU) München, Butenandtstrasse 5-13, 81377 Munich (Germany)

[*] These authors contributed equally.

E-Mail: thomas.carell@lmu.de

Supporting Information

Table of Contents

1. General information and instruments for nucleosides and phosphoramidites 2
2. Synthesis and characterization data of phosphoramidites 2
 2.1 Amino acid-modified methyl N^6-carbamoyl adenosine phosphoramidites 2
 2.2 Amino acid-modified methyl N^6-carbamoyl deoxy adenosine phosphoramidites 17
 2.3 Npe-protected amino acids 28
 2.4 5-Methylaminomethyl uridine phosphoramidite 35
 2.5 5-Aminomethyl thymidine phosphoramidite 35
3. General information and instruments for oligonucleotides 42
4. Synthesized oligonucleotides using a DNA/RNA automated synthesizer 43
 4.1 Donor strands containing amino acid-modified methyl N^6-carbamoyl adenosine at the 5'-end 43
 4.2 Acceptor strands containing 5-methylaminomethyl uridine at the 3'-end 43
 4.3 Donor strands containing amino acid-modified methyl N^6-carbamoyl deoxy adenosine at the 5'-end 43
 4.4 Acceptor strands containing 5-aminomethyl thymidine at the 3'-end 43
5. Peptide coupling reactions between donor and acceptor oligonucleotides 44
6. Calibration curve hairpin 45
7. Cleavage reaction of hairpin products 46
8. Competitive coupling and cleavage reactions (one pot) 47
9. Pair-wise competitive peptide coupling reactions between donor and acceptor oligonucleotides containing dipeptides 48
10. Pair-wise competitive peptide coupling reactions between donor and acceptor deoxy oligonucleotides 51
11. Circular dichroism measurements 52
12. References 52

1. General information and instruments for nucleosides and phosphoramidites

Reagents were purchased from commercial suppliers and used without further purification unless otherwise stated. Anhydrous solvents, stored under inert atmosphere, were also purchased. All reactions involving air/moisture sensitive reagents/intermediates were performed under inert atmosphere using oven-dried glassware. Routine ^{1}H NMR, ^{13}C NMR and ^{31}P NMR spectra were recorded on a Bruker Ascend 500 spectrometer (500 MHz for ^{1}H NMR, 125 MHz for ^{13}C NMR and 202 MHz for ^{31}P NMR). Deuterated solvents used are indicated in the characterization and chemical shifts (δ) are reported in ppm. Residual solvent peaks were used as reference.[1] All NMR *J* values are given in Hz. COSY, HMQC and HMBC NMR experiments were recorded to help with the assignment of ^{1}H and ^{13}C signals. NMR spectra were analysed using MestReNova software version 10.0. High Resolution Mass Spectra (HRMS) were measured on a Thermo Finnigan LTQ-FT with ESI as ionization mode. IR spectra were recorded on a Perkin-Elmer Spectrum BX II FT-IR instrument or Shimadzu IRSpirit FT-IR instrument. Both equipped with an ATR accessory. Column chromatography was performed with technical grade silica gel, 40-63 µm particle size. Reaction progress was monitored by Thin Layer Chromatography (TLC) analysis on silica gel 60 F254 and stained with *para*-anisaldehyde, potassium permanganate or ninhydrin solution.

2. Synthesis and characterization data of phosphoramidites

2.1 Amino acid-modified methyl *N*⁶-carbamoyl adenosine phosphoramidites

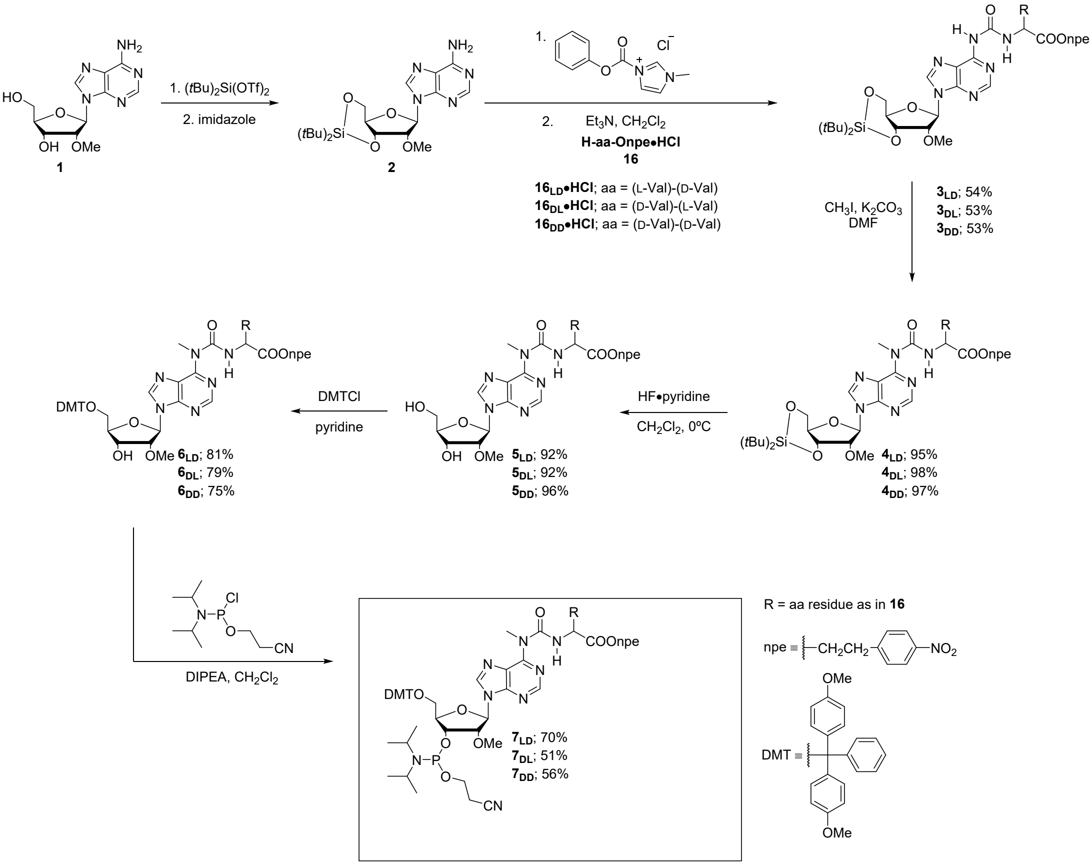

Scheme S1. Synthesis of amino acid-modified methyl *N*⁶-carbamoyl adenosine phosphoramidites **7**.

Compound **2** was synthesized following a procedure previously reported in the literature.[2]

General procedure for the synthesis of compound 3: Step 1. Compound **2** (1 equiv.) and 1-*N*-methyl-3-phenoxycarbonyl-imidazolium chloride (2 equiv.) were added to an oven dried round-bottom flask and kept under high-vacuum for 15 min. After that, dry CH_2Cl_2 was added under nitrogen atmosphere and the reaction was stirred at r.t. for 5 h. Step 2. Onpe-protected amino acid **16•HCl** (2 equiv.) was added to an oven-dried round-bottom flask and suspended in dry CH_2Cl_2, followed by the addition of Et_3N (2 equiv.). The suspension was added dropwise to the reaction mixture. The reaction was stirred at r.t. under nitrogen atmosphere for 20 h. After that, the reaction was quenched with aqueous saturated $NaHCO_3$. The organic layer was separated and the crude was further extracted with CH_2Cl_2. The combined organic layers were dried (Na_2SO_4), filtered and concentrated. The crude was purified by silica gel column chromatography affording the product as a white foam.

3_{LD}: Yield = 54%. Rf = 0.30 (EtOAc). ^{1}H NMR (500 MHz with cryoprobe, $CDCl_3$, 298 K): δ (ppm) = 10.01 (s, 1H); 9.07 (s, 1H); 8.62 (s, 1H); 8.29 (s, 1H); 8.11 – 8.04 (m, 2H); 7.34 (d, *J* = 8.4 Hz, 2H); 6.98 (s, 1H); 6.00 (s, 1H); 4.65 (s, 1H); 4.53 (dd, *J* = 8.7 Hz, *J* = 5.0 Hz, 1H); 4.46 (dd, *J* = 9.2 Hz, *J* = 5.0 Hz, 2H); 4.38 – 4.32 (m, 2H); 4.27 (d, *J* = 4.6 Hz, 1H); 4.18 (td, *J* = 10.1 Hz, *J* = 5.0 Hz, 1H); 4.03 (dd, *J* = 10.5 Hz, *J* = 9.2 Hz, 1H); 3.68 (s, 3H); 3.03 (hept, *J* = 8.1 Hz, *J* = 7.4 Hz, 2H); 2.36 (h, *J* = 6.7 Hz, 1H); 2.11 (pd, *J* = 6.9 Hz, *J* = 5.2 Hz, 1H); 1.09 (s, 9H); 1.05 (s, 9H); 1.03 (d, *J* = 9.2 Hz, 6H); 0.90 (d, *J* = 6.8 Hz, 3H); 0.83 (d, *J* = 6.9 Hz, 3H). ^{13}C{^{1}H} NMR (125 MHz with cryoprobe, $CDCl_3$, 298 K): δ (ppm) = 171.7; 171.6; 154.4; 150.0; 147.0; 145.5; 129.8; 123.8; 121.1; 89.9; 82.3; 77.4; 74.9; 67.5; 64.7; 60.1; 59.6; 57.4; 34.8; 31.1; 27.5; 27.2; 22.9; 20.5; 19.8; 19.2; 18.1; 17.9. FTIR ν_{max} (cm^{-1}): 2933 (w); 2860 (w); 1677 (m); 1520 (s); 1467 (m); 1345 (s); 1257 (m); 1138 (s); 1063 (s); 828 (s). HRMS (ESI) *m/z*: $[M+Na]^+$ Calcd for $C_{38}H_{56}O_{10}N_8SiNa$ 835.3780; Found 835.3803.

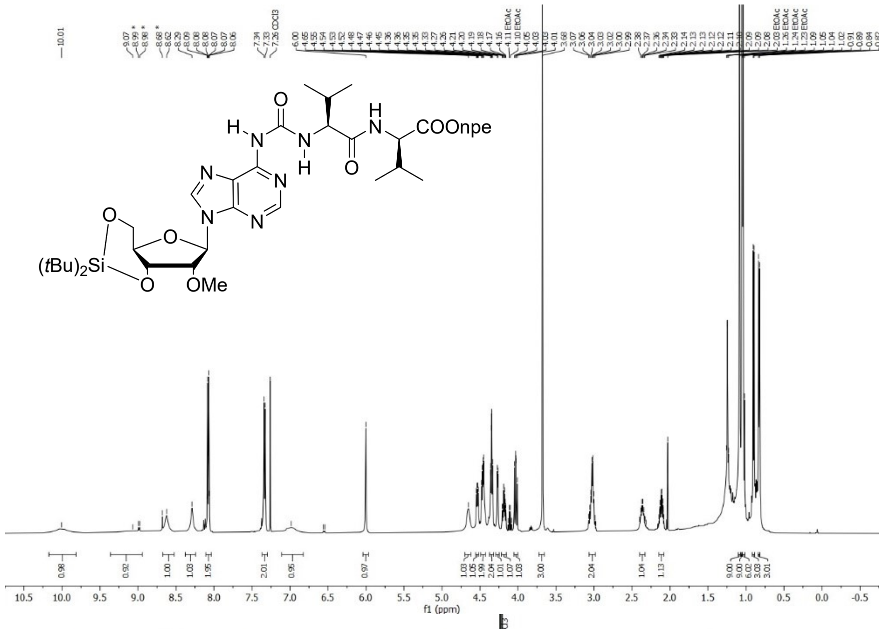

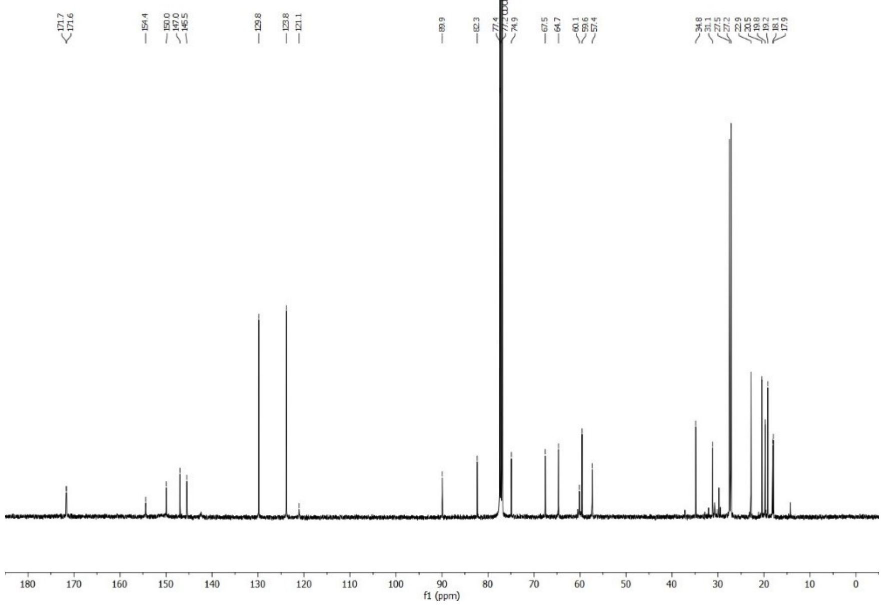

3_{DL}: Yield = 53%. Rf = 0.36 (EtOAc). ^{1}H NMR (500 MHz with cryoprobe, $CDCl_3$, 298 K): δ (ppm) = 10.03 (s, 1H); 9.20 (s, 1H); 8.59 (s, 1H); 8.30 (s, 1H); 8.10 – 8.05 (m, 2H); 7.35 – 7.31 (m, 2H); 6.94 (s, 1H); 6.00 (s, 1H); 4.73 (dd, *J* = 9.6 Hz, *J* = 4.7 Hz, 1H); 4.56 – 4.51 (m, 1H); 4.47 – 4.31 (m, 4H); 4.31 – 4.27 (m, 1H); 4.16 (td, *J* = 10.1 Hz, *J* = 5.0 Hz, 1H); 4.02 (dd, *J* = 10.5 Hz, *J* = 9.2 Hz, 1H); 3.67 (s, 3H); 3.02 (td, *J* = 6.7 Hz, *J* = 3.8 Hz, 2H); 2.42 – 2.32 (m, 1H); 2.15 – 2.07 (m, 1H); 1.09 (s, 9H); 1.06 – 1.03 (m, 15H); 0.90 (d, *J* = 6.8 Hz, 3H); 0.83 (d, *J* = 6.9 Hz, 3H). ^{13}C{^{1}H} NMR (125 MHz with cryoprobe, $CDCl_3$, 298 K): δ (ppm) = 171.8; 171.6; 154.5; 150.0; 147.0; 145.4; 129.8; 123.9; 123.8; 121.1; 89.9; 82.3; 77.4; 74.9; 67.5; 64.7; 60.1; 59.5; 57.3; 34.8; 31.2; 27.5; 27.4; 27.2; 22.9; 22.8; 20.4; 19.8; 19.2; 18.2; 17.9. FTIR ν_{max} (cm^{-1}): 2934 (w); 2860 (w); 1677 (m); 1520 (s); 1466 (m); 1345 (s); 1134 (m); 1063 (m); 828 (m). HRMS (ESI) *m/z*: $[M+Na]^+$ Calcd for $C_{38}H_{56}O_{10}N_8SiNa$ 835.3780; Found 835.3801.

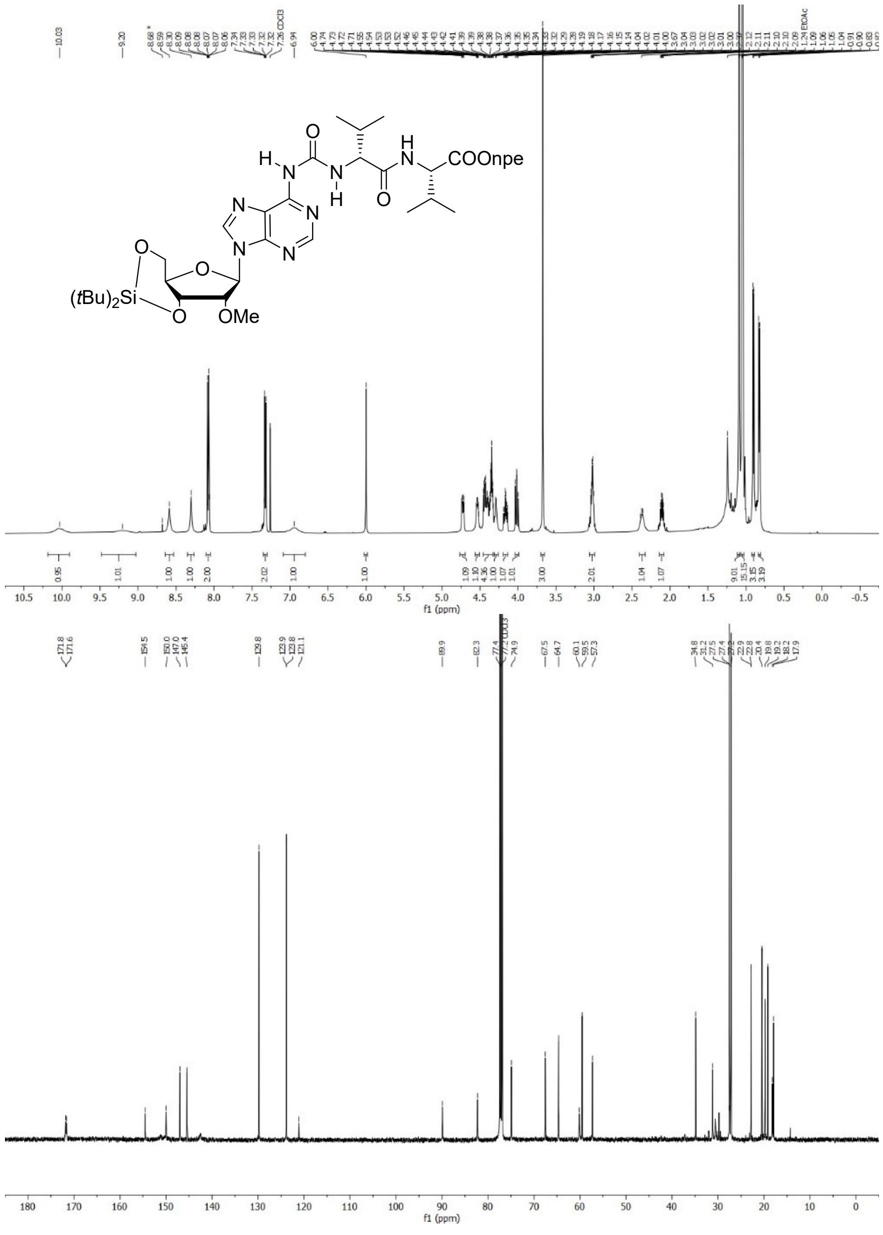

3_{DD}: Yield = 53%. Rf = 0.38 (EtOAc). ^{1}H NMR (500 MHz with cryoprobe, $CDCl_3$, 298 K): δ (ppm) = 10.06 (s, 1H); 9.36 (s, 1H); 8.59 (s, 1H); 8.32 (t, *J* = 8.1 Hz, 1H); 8.12 (td, *J* = 5.3 Hz, *J* = 2.6 Hz, 2H); 7.39 – 7.35 (m, 2H); 6.97 (s, 1H); 6.00 (s, 1H); 4.70 (dd, *J* = 10.0 Hz, *J* = 4.6 Hz, 1H); 4.49 (dd, *J* = 8.4 Hz, *J* = 5.1 Hz, 1H); 4.44 (dt, *J* = 6.8 Hz, *J* = 3.3 Hz, 2H); 4.38 (td, *J* = 6.7 Hz, *J* = 3.9 Hz, 2H); 4.26 (d, *J* = 4.6 Hz, 1H); 4.16 (td, *J* = 10.0 Hz, *J* = 4.9 Hz, 1H); 4.02 (dd, *J* = 10.5 Hz, *J* = 9.2 Hz, 1H); 3.66 (s, 3H); 3.06 (t, *J* = 6.7 Hz, 2H); 2.37 (h, *J* = 6.8 Hz, 1H); 2.12 – 2.05 (m, 1H); 1.08 (s, 9H); 1.06 – 1.00 (m, 15H); 0.86 (d, *J* = 6.8 Hz, 3H); 0.81 (d, *J* = 6.7 Hz, 3H). ^{13}C{^{1}H} NMR (125 MHz with cryoprobe, $CDCl_3$, 298 K): δ (ppm) = 171.8; 171.6; 154.5; 150.0; 147.0; 145.4; 129.8; 123.8; 121.1; 89.9; 82.3; 74.9; 67.5; 64.6; 60.1; 59.5; 57.4; 34.9; 31.0; 27.5; 27.2; 22.8; 20.4; 19.7; 19.1; 18.1; 17.9. FTIR ν_{max} (cm^{-1}): 2934 (w); 2860 (w); 1676 (m); 1520 (s); 1467 (m); 1345 (s); 1138 (m); 1063 (m); 828 (m). HRMS (ESI) *m/z*: $[M+Na]^+$ Calcd for $C_{38}H_{56}O_{10}N_8SiNa$ 835.3780; Found 835.3802.

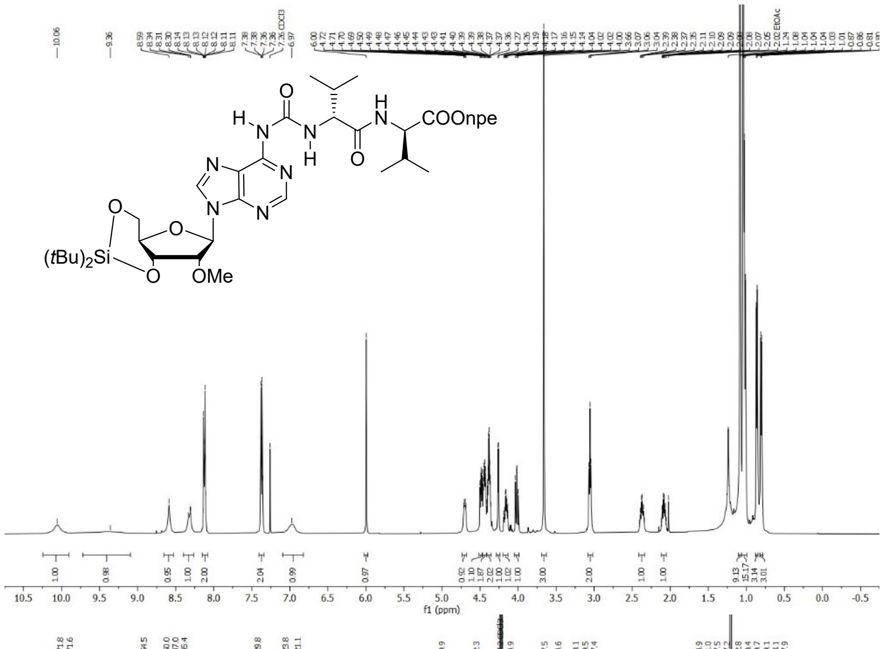

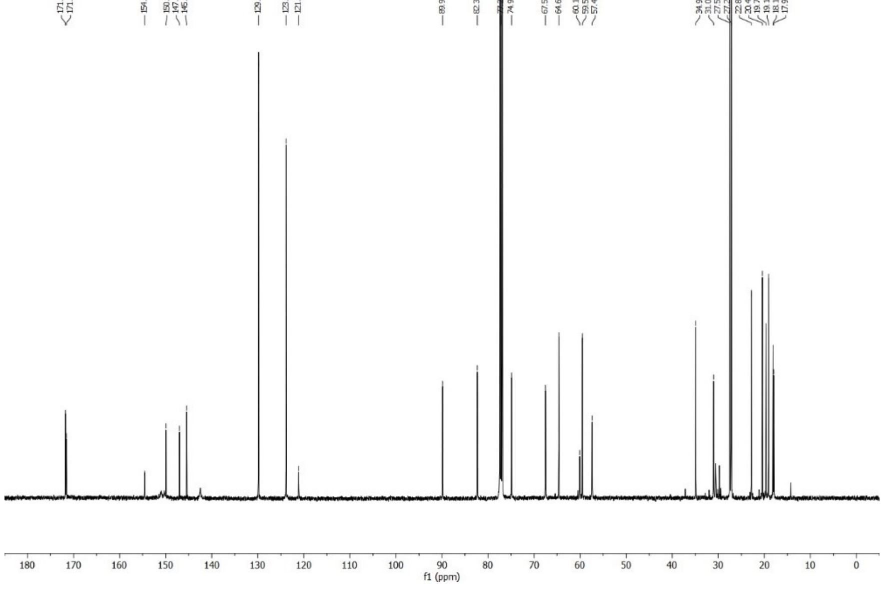

General procedure for the synthesis of compound 4: Compound **3** (1 equiv.) and K_2CO_3 (3 equiv.) were suspended in dry DMF. The suspension was stirred for 30 min at 0°C under nitrogen atmosphere. After that, CH_3I (2 equiv.) was added dropwise and the reaction was stirred at r.t. overnight. After that, the reaction mixture was diluted with CH_2Cl_2 and washed with aqueous saturated NH_4Cl and water. The organic layer was dried (Na_2SO_4), filtered and concentrated. The crude was purified by silica gel column chromatography affording the product as a white foam.

4_{LD}: Yield = 95%. Rf = 0.57 (3:7 iHex/EtOAc). ^1H NMR (500 MHz with cryoprobe, $CDCl_3$, 298 K): δ (ppm) = 10.61 (s, 1H); 8.56 (s, 1H); 8.15 – 8.11 (m, 2H); 8.02 (s, 1H); 7.38 – 7.34 (m, 2H); 6.90 (d, *J* = 8.7 Hz, 1H); 6.01 (s, 1H); 4.59 (dd, *J* = 9.7 Hz, *J* = 4.6 Hz, 1H); 4.54 (dd, *J* = 8.7 Hz, *J* = 5.0 Hz, 1H); 4.48 (dd, *J* = 9.2 Hz, *J* = 5.0 Hz, 1H); 4.37 (t, *J* = 6.7 Hz, 3H); 4.23 (d, *J* = 4.6 Hz, 1H); 4.19 (td, *J* = 10.1 Hz, *J* = 5.0 Hz, 1H); 4.01 (dd, *J* = 10.5 Hz, *J* = 9.2 Hz, 1H); 3.96 (s, 3H); 3.68 (s, 3H); 3.04 (td, *J* = 6.7 Hz, *J* = 1.6 Hz, 2H); 2.40 (pd, *J* = 6.9 Hz, *J* = 5.1 Hz, 1H); 2.09 (pd, *J* = 6.9 Hz, *J* = 5.0 Hz, 1H); 1.08 (s, 9H); 1.05 (s, 9H); 1.01 (dd, *J* = 14.0 Hz, *J* = 6.8 Hz, 6H); 0.89 (d, *J* = 6.8 Hz, 3H); 0.78 (d, *J* = 6.9 Hz, 3H). $^{13}C\{^1H\}$ NMR (125 MHz with cryoprobe, $CDCl_3$, 298 K): δ (ppm) = 171.9; 171.9; 156.2; 153.3; 151.7; 150.5; 147.0; 145.4; 139.9; 129.8; 123.9; 122.7; 89.8; 82.3; 77.3; 74.8; 67.6; 64.6; 61.2; 59.5; 57.2; 34.9; 31.2; 30.3; 27.5; 27.2; 22.9; 20.5; 19.9; 19.1; 17.9; 17.8. FTIR ν_{max} (cm^{-1}): 2934 (w); 2860 (w); 1670 (m); 1578 (m); 1520 (s); 1466 (m); 1345 (m); 1137 (m); 1065 (m); 1012 (m); 828 (m). HRMS (ESI) *m/z*: $[M+Na]^+$ Calcd for $C_{39}H_{58}O_{10}N_8SiNa$ 849.3934; Found 849.3958.

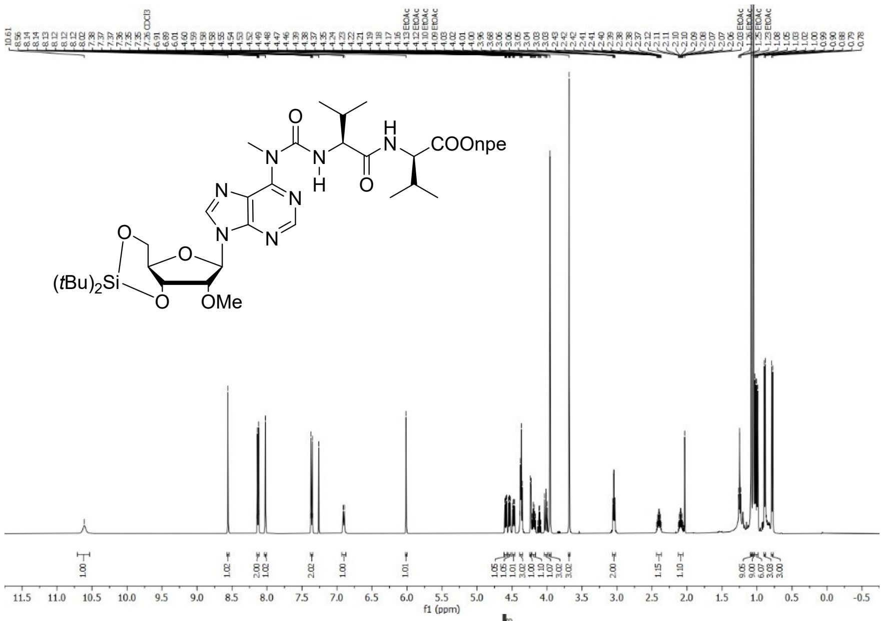

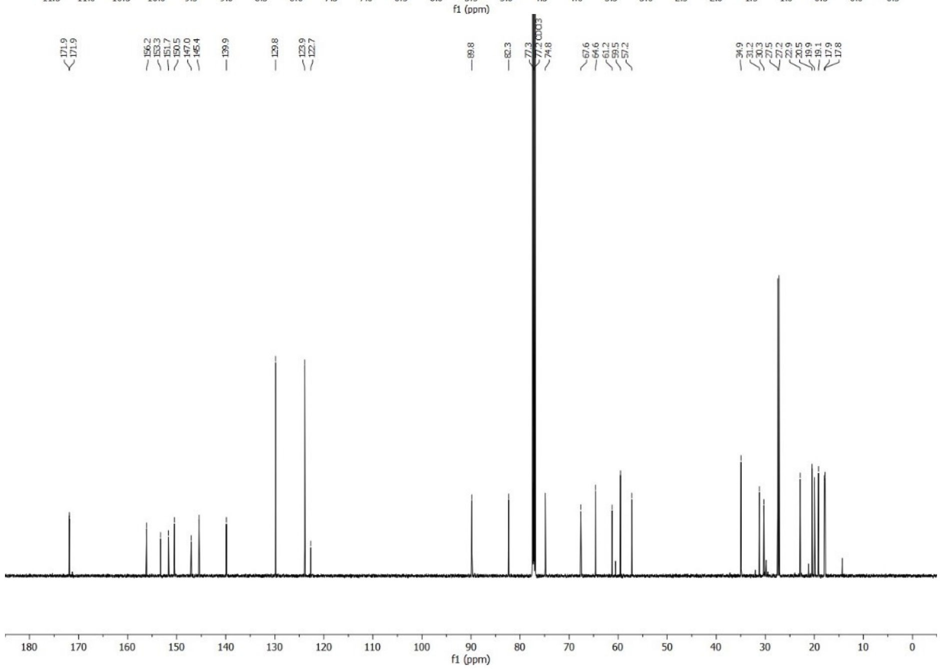

4_{DL}: Yield = 98%. Rf = 0.64 (3:7 iHex/EtOAc). ^{1}H NMR (500 MHz with cryoprobe, $CDCl_3$, 298 K): δ (ppm) = 10.93 (s, 1H); 8.54 (s, 1H); 8.17 – 8.13 (m, 2H); 8.00 (s, 1H); 7.40 – 7.37 (m, 2H); 6.70 (d, *J* = 8.5 Hz, 1H); 6.02 (s, 1H); 4.59 (dd, *J* = 9.7 Hz, *J* = 4.6 Hz, 1H); 4.51 – 4.46 (m, 2H); 4.39 (q, *J* = 6.8 Hz, 2H); 4.35 (dd, *J* = 6.3 Hz, *J* = 2.8 Hz, 1H); 4.24 (d, *J* = 4.6 Hz, 1H); 4.19 (td, *J* = 10.1 Hz, *J* = 5.0 Hz, 1H); 4.01 (dd, *J* = 10.6 Hz, *J* = 9.4 Hz, 1H); 3.98 (s, 3H); 3.68 (s, 3H); 3.07 (td, *J* = 6.6 Hz, *J* = 1.4 Hz, 2H); 2.42 – 2.34 (m, 1H); 2.09 (pd, *J* = 6.9 Hz, *J* = 5.0 Hz, 1H); 1.09 (s, 9H); 1.05 (s, 9H); 1.02 (dd, *J* = 6.8 Hz, *J* = 5.4 Hz, 6H); 0.87 (d, *J* = 6.9 Hz, 3H); 0.81 (d, *J* = 6.9 Hz, 3H). $^{13}C\{^{1}H\}$ NMR (125 MHz with cryoprobe, $CDCl_3$, 298 K): δ (ppm) = 171.9; 171.8; 156.2; 153.3; 151.7; 150.3; 147.1; 145.5; 139.7; 129.9; 123.9; 122.7; 89.8; 82.3; 77.3; 74.8; 67.6; 64.7; 61.1; 59.5; 57.3; 35.0; 35.0; 31.2; 30.3; 27.5; 27.2; 22.9; 20.5; 19.8; 19.1; 18.3; 17.9. FTIR ν_{max} (cm^{-1}): 2934 (w); 2860 (w); 1670 (m); 1578 (m); 1520 (s); 1466 (m); 1345 (m); 1137 (m); 1065 (m); 1013 (m); 828 (m). HRMS (ESI) *m/z*: $[M+Na]^+$ Calcd for $C_{39}H_{58}O_{10}N_8SiNa$ 849.3934; Found 849.3957.

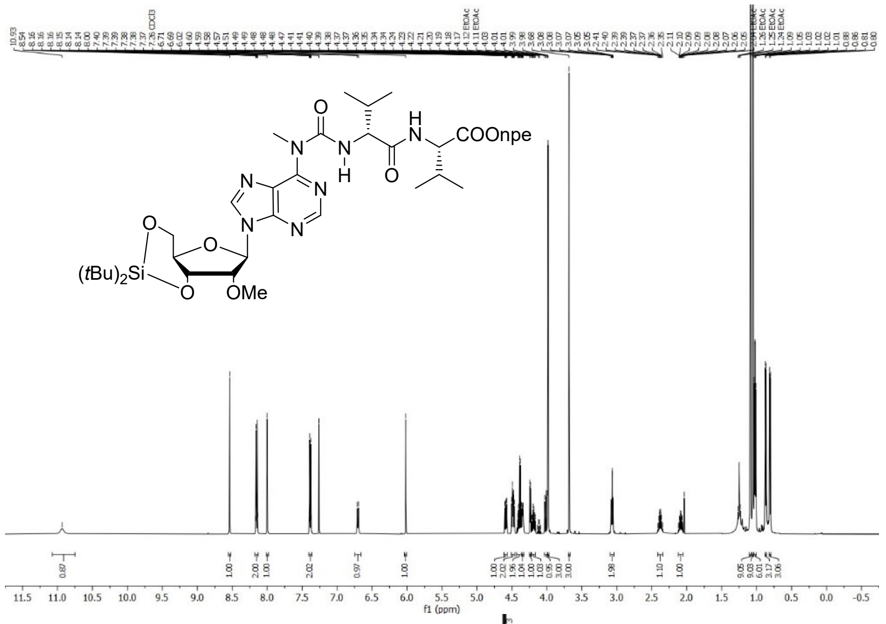

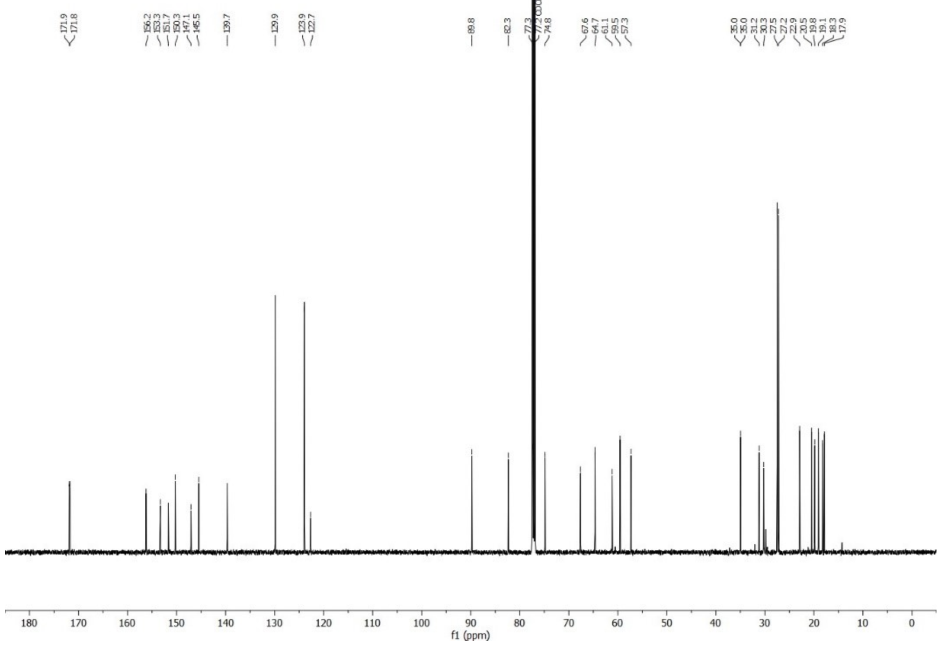

4_{DD}: Yield = 97%. Rf = 0.64 (3:7 iHex/EtOAc). ^{1}H NMR (500 MHz with cryoprobe, $CDCl_3$, 298 K): δ (ppm) = 10.61 (s, 1H); 8.56 (s, 1H); 8.14 – 8.10 (m, 2H); 8.01 (s, 1H); 7.38 – 7.35 (m, 2H); 6.88 (d, J = 8.8 Hz, 1H); 6.02 (s, 1H); 4.60 (dd, J = 9.7 Hz, J = 4.7 Hz, 1H); 4.54 (dd, J = 8.8 Hz, J = 4.9 Hz, 1H); 4.48 (dd, J = 9.2 Hz, J = 5.0 Hz, 1H); 4.37 (t, J = 6.6 Hz, 3H); 4.24 (d, J = 4.6 Hz, 1H); 4.19 (td, J = 10.1 Hz, J = 5.0 Hz, 1H); 4.01 (dd, J = 10.6 Hz, J = 9.2 Hz, 1H); 3.95 (s, 3H); 3.68 (s, 3H); 3.05 (td, J = 6.7 Hz, J = 2.1 Hz, 2H); 2.39 (pd, J = 6.9 Hz, J = 5.1 Hz, 1H); 2.09 (pd, J = 6.9 Hz, J = 4.9 Hz, 1H); 1.08 (s, 9H); 1.05 (s, 9H); 1.01 (dd, J = 15.6 Hz, J = 6.8 Hz, 6H); 0.89 (d, J = 6.8 Hz, 3H); 0.78 (d, J = 6.9 Hz, 3H). ^{13}C{^{1}H} NMR (125 MHz with cryoprobe, $CDCl_3$, 298 K): δ (ppm) = 171.9; 171.9; 156.1; 153.3; 151.7; 150.5; 147.0; 145.4; 139.9; 129.8; 123.9; 122.7; 89.8; 82.3; 77.3; 74.8; 67.6; 64.6; 61.2; 59.5; 57.2; 34.9; 34.9; 31.2; 30.3; 27.5; 27.2; 22.9; 20.5; 19.9; 19.1; 18.0; 17.8. FTIR ν_{max} (cm^{-1}): 2934 (w); 2860 (w); 1663 (m); 1559 (m); 1520 (s); 1466 (m); 1345 (m); 1137 (m); 1065 (m); 1013 (m); 828 (m). HRMS (ESI) m/z: $[M+Na]^+$ Calcd for $C_{39}H_{58}O_{10}N_8SiNa$ 849.3934; Found 849.3960.

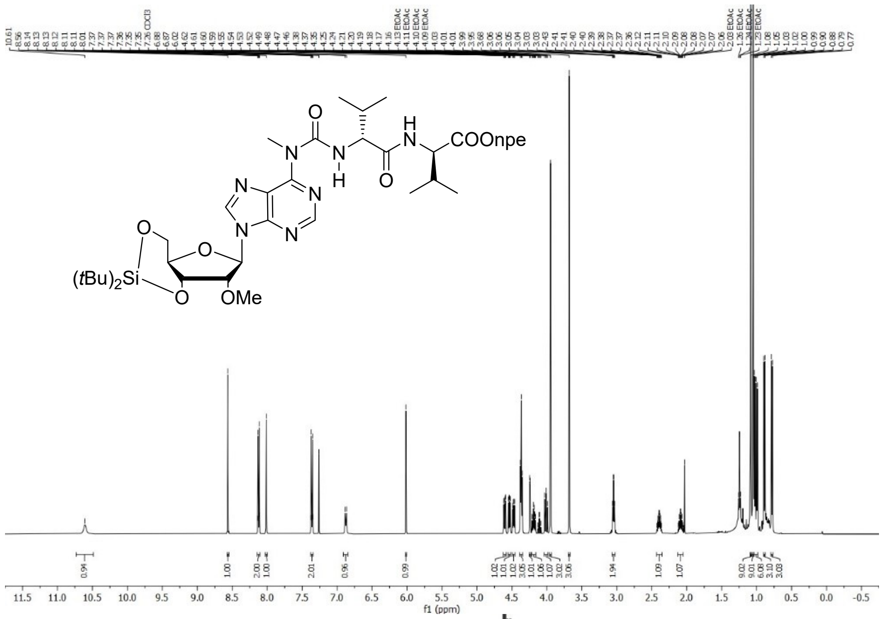

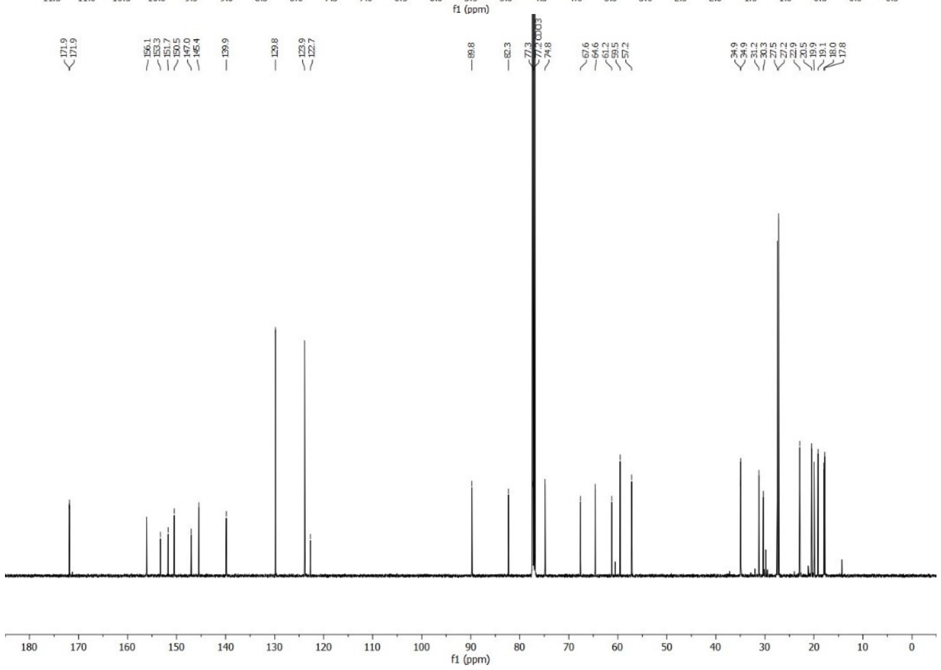

General procedure for the synthesis of compound 5: Compound **4** (1 equiv.) was added to a plastic flask and dissolved in dry 9:1 CH_2Cl_2/pyridine. The solution was stirred at 0°C. Finally, HF•pyridine (from a commercial solution containing 70% HF and 30% pyridine) was added and the reaction was stirred at 0°C for 2 h. After that, the reaction was quenched with aqueous saturated $NaHCO_3$ and CH_2Cl_2 was added. The organic layer was separated and the crude was further extracted with CH_2Cl_2. The combined organic layers were dried (Na_2SO_4), filtered and concentrated. The crude was purified by silica gel column chromatography affording the product as a white foam.

5_{LD}: Yield = 92%. Rf = 0.38 (95:5 CH_2Cl_2:MeOH). ^{1}H NMR (500 MHz with cryoprobe, $CDCl_3$, 298 K): δ (ppm) = 10.41 (s, 1H); 8.58 (s, 1H); 8.18 (s, 1H); 8.14 – 8.09 (m, 2H); 7.39 – 7.34 (m, 2H); 6.90 (d, J = 8.7 Hz, 1H); 5.96 (d, J = 6.9 Hz, 1H); 4.67 (dd, J = 6.9 Hz, J = 4.6 Hz, 1H); 4.60 (d, J = 4.2 Hz, 1H); 4.53 (dd, J = 8.8 Hz, J = 4.9 Hz, 1H); 4.41 – 4.33 (m, 4H); 3.98 – 3.92 (m, 4H); 3.79 (dd, J = 13.0 Hz, J = 1.7 Hz, 1H); 3.38 (s, 3H); 3.07 – 3.02 (m, 2H); 2.41 – 2.33 (m, 1H); 2.08 (pd, J = 6.9 Hz, J = 4.9 Hz, 1H); 2.02 (s, 1H); 1.00 (dd, J = 17.5 Hz, J = 6.8 Hz, 6H); 0.88 (d, J = 6.9 Hz, 3H); 0.77 (d, J = 6.8 Hz, 3H). $^{13}C\{^1H\}$ NMR (125 MHz with cryoprobe, $CDCl_3$, 298 K): δ (ppm) = 171.9; 171.8; 155.9; 153.7; 151.1; 150.0; 147.0; 145.4; 141.7; 129.8; 123.9; 89.5; 88.0; 82.5; 77.4; 70.3; 64.7; 63.1; 61.2; 59.0; 57.2; 35.0; 34.9; 31.2; 30.4; 19.9; 19.1; 17.9; 17.8. FTIR ν_{max} (cm^{-1}): 2926 (w); 1663 (m); 1518 (s); 1464 (m); 1345 (m); 1266 (m); 1016 (m). HRMS (ESI) m/z: $[M+Na]^+$ Calcd for $C_{31}H_{42}O_{10}N_8Na$ 709.2916; Found 709.2918.

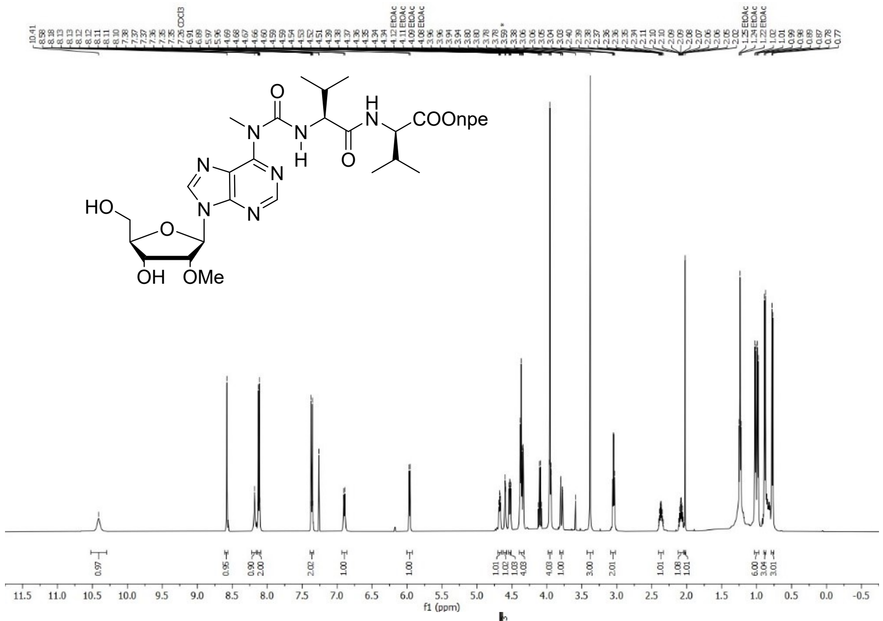

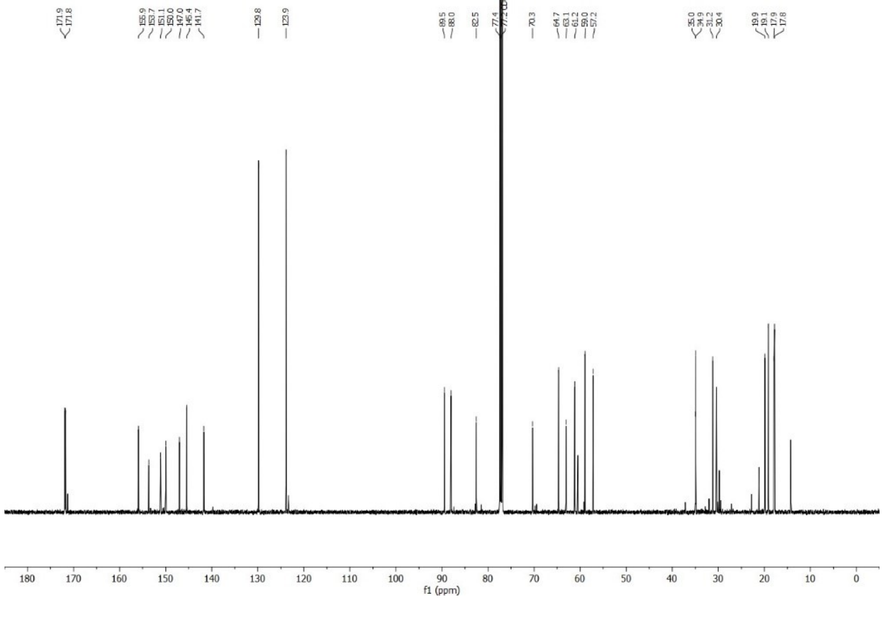

5_{DL}: Yield = 92%. Rf = 0.36 (95:5 CH_2Cl_2:MeOH). ^1H NMR (500 MHz with cryoprobe, $CDCl_3$, 298 K): δ (ppm) = 10.32 (s, 1H); 8.58 (s, 1H); 8.23 (s, 1H); 8.14 – 8.11 (m, 2H); 7.38 – 7.34 (m, 2H); 6.92 (d, J = 8.8 Hz, 1H); 5.97 (d, J = 6.7 Hz, 1H); 4.65 (dd, J = 6.8 Hz, J = 4.6 Hz, 1H); 4.59 (dd, J = 4.7 Hz, J = 1.6 Hz, 1H); 4.52 (dd, J = 8.8 Hz, J = 4.9 Hz, 1H); 4.39 – 4.33 (m, 4H); 3.98 – 3.94 (m, 4H); 3.79 (dd, J = 13.0 Hz, J = 1.7 Hz, 1H); 3.38 (s, 3H); 3.04 (td, J = 6.7 Hz, J = 1.5 Hz, 2H); 2.37 (pd, J = 6.8 Hz, J = 5.2 Hz, 1H); 2.08 (pd, J = 6.9 Hz, J = 5.0 Hz, 1H); 1.02 (d, J = 6.8 Hz, 3H); 0.99 (d, J = 6.8 Hz, 3H); 0.88 (d, J = 6.9 Hz, 3H); 0.78 (d, J = 6.9 Hz, 3H). $^{13}C\{^1H\}$ NMR (125 MHz with cryoprobe, $CDCl_3$, 298 K): δ (ppm) = 171.9; 171.8; 155.9; 153.6; 151.1; 150.1; 147.0; 145.4; 141.7; 129.9; 129.8; 123.9; 89.5; 88.0; 82.6; 77.4; 70.2; 64.7; 63.0; 61.2; 58.9; 57.2; 35.0; 34.9; 31.2; 30.3; 19.9; 19.1; 18.0; 17.8. FTIR ν_{max} (cm^{-1}): 2929 (w); 1663 (m); 1518 (s); 1464 (m); 1345 (m); 1016 (m). HRMS (ESI) m/z: $[M+Na]^+$ Calcd for $C_{31}H_{42}O_{10}N_8Na$ 709.2916; Found 709.2918.

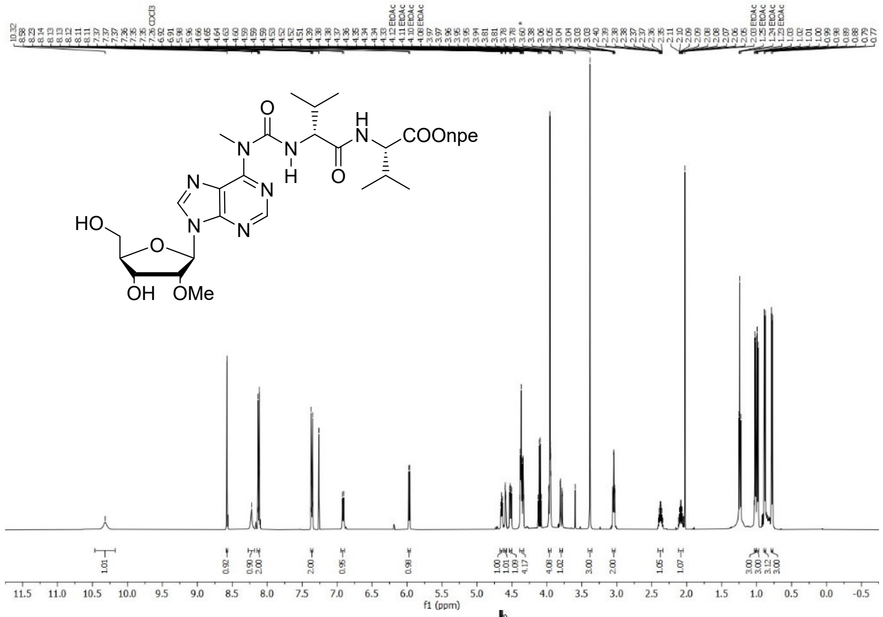

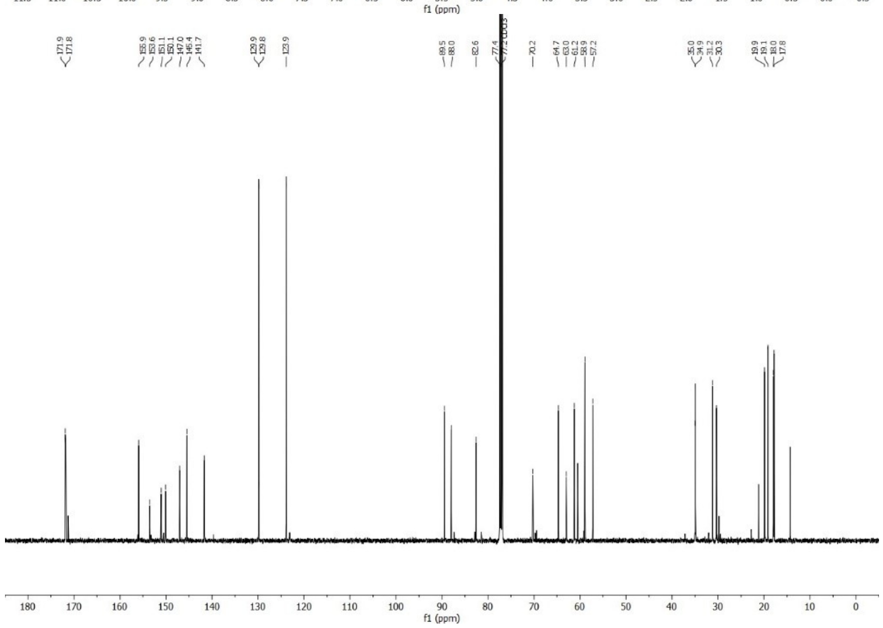

5_{DD}: Yield = 96%. Rf = 0.37 (95:5 CH_2Cl_2:MeOH). ^1H NMR (500 MHz with cryoprobe, $CDCl_3$, 298 K): δ (ppm) = 10.72 (s, 1H); 8.55 (s, 1H); 8.18 (s, 1H); 8.16 – 8.12 (m, 2H); 7.40 – 7.36 (m, 2H); 6.76 (d, J = 8.4 Hz, 1H); 5.96 (d, J = 6.8 Hz, 1H); 4.64 (dd, J = 6.9 Hz, J = 4.6 Hz, 1H); 4.58 (dd, J = 4.7 Hz, J = 1.6 Hz, 1H); 4.48 (dd, J = 8.5 Hz, J = 5.0 Hz, 1H); 4.41 – 4.32 (m, 4H); 3.98 (s, 3H); 3.95 (dd, J = 13.0 Hz, J = 1.9 Hz, 1H); 3.78 (dd, J = 13.0 Hz, J = 1.7 Hz, 1H); 3.38 (s, 3H); 3.06 (td, J = 6.7 Hz, J = 1.9 Hz, 2H); 2.39 – 2.31 (m, 1H); 2.08 (pd, J = 6.9 Hz, J = 5.0 Hz, 1H); 1.01 (dd, J = 6.8 Hz, J = 3.8 Hz, 6H); 0.86 (d, J = 6.8 Hz, 3H); 0.80 (d, J = 6.9 Hz, 3H). $^{13}C\{^1H\}$ NMR (125 MHz with cryoprobe, $CDCl_3$, 298 K): δ (ppm) = 171.8; 171.7; 156.0; 153.6; 151.1; 149.8; 147.0; 145.4; 141.5; 129.9; 123.9; 89.4; 88.0; 82.6; 77.4; 70.2; 64.7; 63.0; 61.2; 58.9; 57.3; 35.0; 34.9; 31.1; 30.3; 19.8; 19.1; 18.3; 17.9. FTIR ν_{max} (cm^{-1}): 2927 (w); 1654 (m); 1518 (s); 1464 (m); 1345 (m); 1016 (m). HRMS (ESI) m/z: $[M+Na]^+$ Calcd for $C_{31}H_{42}O_{10}N_8Na$ 709.2916; Found 709.2917.

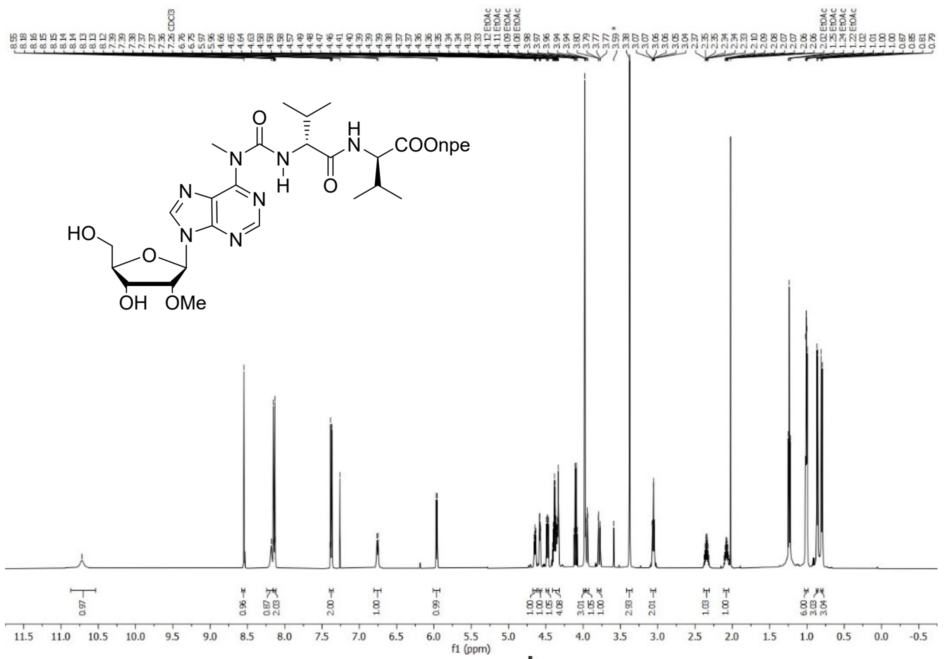

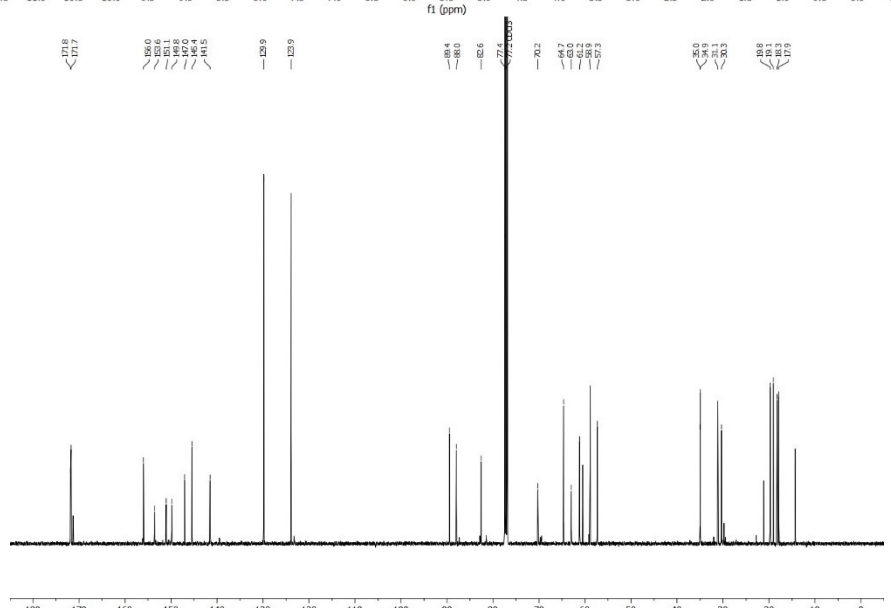

General procedure for the synthesis of compound 6: Compound **5** (1 equiv.) was dissolved in dry pyridine and stirred under nitrogen atmosphere at r.t. 4,4-Dimethoxytrityl chloride (1.5 equiv.) was added in two portions and the reaction was stirred at r.t. overnight. After that, the crude was concentrated and purified by silica gel column chromatography (eluent containing 0.1% pyridine) affording the product as a white foam.

6_{LD}: Yield = 81%. Rf = 0.53 (1:1 iHex/Acetone). ^{1}H NMR (500 MHz with cryoprobe, acetone-d_6, 298 K): δ (ppm) = 10.47 (d, J = 7.8 Hz, 1H); 8.55 (s, 1H); 8.45 (s, 1H); 8.17 – 8.11 (m, 2H); 7.74 – 7.70 (m, 1H); 7.57 – 7.53 (m, 2H); 7.51 – 7.45 (m, 2H); 7.38 – 7.33 (m, 4H); 7.30 – 7.25 (m, 2H); 7.23 – 7.18 (m, 1H); 6.87 – 6.82 (m, 4H); 6.25 (d, J = 4.2 Hz, 1H); 4.66 (dt, J = 6.4 Hz, J = 5.1 Hz, 1H); 4.61 (t, J = 4.6 Hz, 1H); 4.47 (dd, J = 7.8 Hz, J = 4.9 Hz, 1H); 4.44 – 4.33 (m, 3H); 4.25 (q, J = 4.6 Hz, 1H); 4.22 (d, J = 6.4 Hz, 1H); 3.91 (s, 3H); 3.77 (d, J = 1.5 Hz, 6H); 3.51 (s, 3H); 3.48 – 3.45 (m, 2H); 3.10 (t, J = 6.5 Hz, 2H); 2.29 (pd, J = 6.9 Hz, J = 4.9 Hz, 1H); 2.13 – 2.06 (m, 1H); 1.01 (dd, J = 12.5 Hz, J = 6.8 Hz, 6H); 0.89 (d, J = 6.8 Hz, 3H); 0.84 (d, J = 6.8 Hz, 3H). ^{13}C{^{1}H} NMR (125 MHz with cryoprobe, acetone-d_6, 298 K): δ (ppm) = 172.3; 159.6; 159.6; 156.4; 154.1; 153.1; 151.0; 150.7; 147.7; 147.4; 146.1; 141.9; 136.8; 136.7; 131.1; 131.0; 131.0; 129.0; 128.6; 127.6; 124.2; 123.4; 113.9; 87.7; 87.1; 85.0; 83.6; 70.6; 65.2; 64.4; 61.2; 58.8; 58.3; 55.5; 35.3; 35.0; 31.9; 31.4; 20.1; 19.5; 18.3; 18.1. FTIR v_{max} (cm^{-1}): 2960 (w); 2930 (w); 1670 (m); 1576 (m); 1507 (s); 1464 (m); 1345 (m); 1249 (m); 1177 (m); 1033 (m). HRMS (ESI) m/z: [M+H]$^+$ Calcd for $C_{52}H_{61}O_{12}N_8$ 989.4403; Found 989.4419.

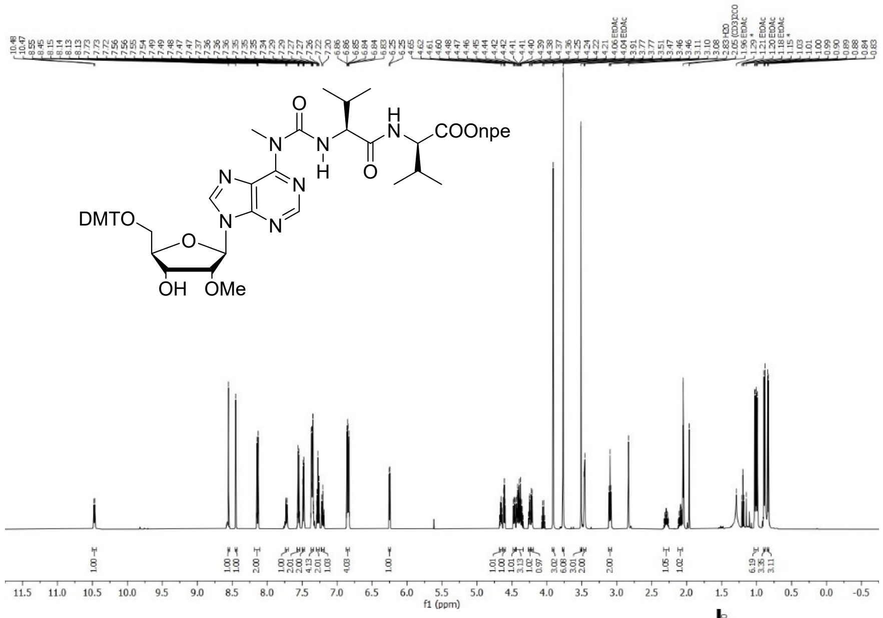

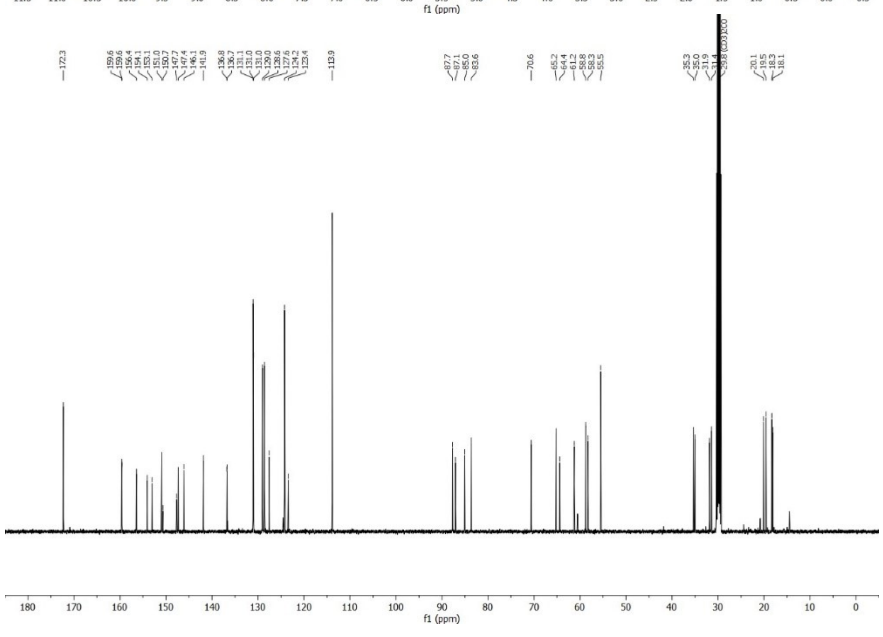

6_{DL}: Yield = 79%. Rf = 0.53 (1:1 iHex/Acetone). ^{1}H NMR (500 MHz with cryoprobe, acetone-d_6, 298 K): δ (ppm) = 10.44 (d, J = 7.7 Hz, 1H); 8.56 (s, 1H); 8.45 (s, 1H); 8.16 – 8.11 (m, 2H); 7.76 – 7.72 (m, 1H); 7.57 – 7.53 (m, 2H); 7.50 – 7.46 (m, 2H); 7.36 (dq, J = 8.5 Hz, J = 3.2 Hz, 4H); 7.30 – 7.25 (m, 2H); 7.23 – 7.19 (m, 1H); 6.87 – 6.82 (m, 4H); 6.26 (d, J = 4.3 Hz, 1H); 4.65 (dt, J = 6.4 Hz, J = 5.1 Hz, 1H); 4.60 (t, J = 4.7 Hz, 1H); 4.46 (dd, J = 7.8 Hz, J = 4.9 Hz, 1H); 4.44 – 4.33 (m, 3H); 4.27 – 4.23 (m, 1H); 4.22 (d, J = 6.4 Hz, 1H); 3.90 (s, 3H); 3.77 (d, J = 1.9 Hz, 6H); 3.51 (s, 3H); 3.46 (dd, J = 4.4 Hz, J = 1.5 Hz, 2H); 3.09 (t, J = 6.4 Hz, 2H); 2.28 (pd, J = 6.9 Hz, J = 4.8 Hz, 1H); 2.10 (qd, J = 7.0 Hz, J = 5.9 Hz, 1H); 1.00 (dd, J = 11.5 Hz, J = 6.9 Hz, 6H); 0.89 (d, J = 6.9 Hz, 3H); 0.84 (d, J = 6.8 Hz, 3H). ^{13}C{^{1}H} NMR (125 MHz with cryoprobe, acetone-d_6, 298 K): δ (ppm) = 172.3; 159.6; 159.6; 156.4; 154.1; 153.1; 151.0; 147.7; 147.4; 146.1; 141.8; 136.8; 136.7; 131.1; 131.0; 131.0; 129.0; 128.6; 127.6; 124.2; 123.5; 113.9; 87.6; 87.1; 85.1; 83.7; 70.6; 65.2; 64.4; 61.2; 58.8; 58.3; 55.5; 35.3; 35.0; 31.9; 31.4; 20.1; 19.5; 18.3; 18.1. FTIR ν_{max} (cm^{-1}): 2960 (w); 2930 (w); 1670 (m); 1576 (m); 1507 (s); 1464 (m); 1345 (m); 1250 (m); 1177 (m); 1033 (m). HRMS (ESI) m/z: $[M+H]^+$ Calcd for $C_{52}H_{61}O_{12}N_8$ 989.4403; Found 989.4421.

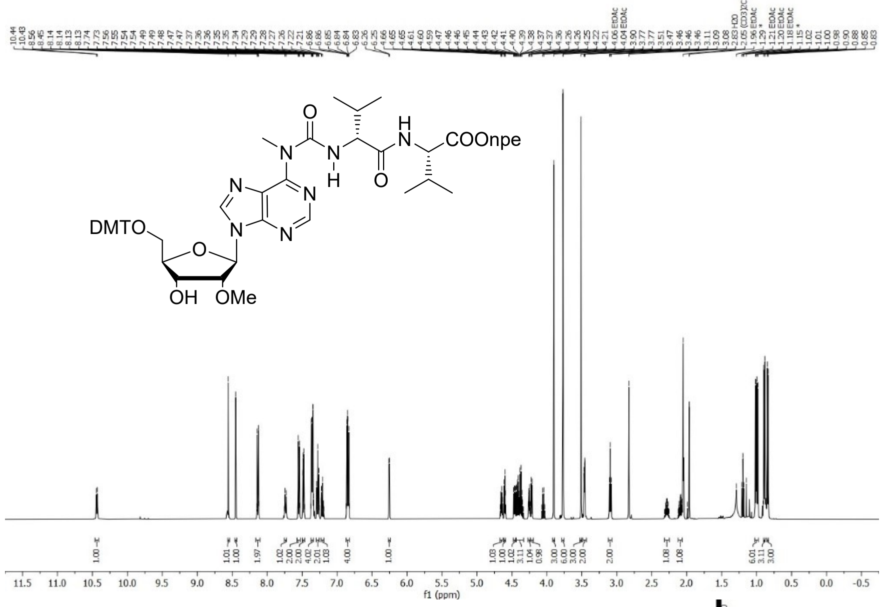

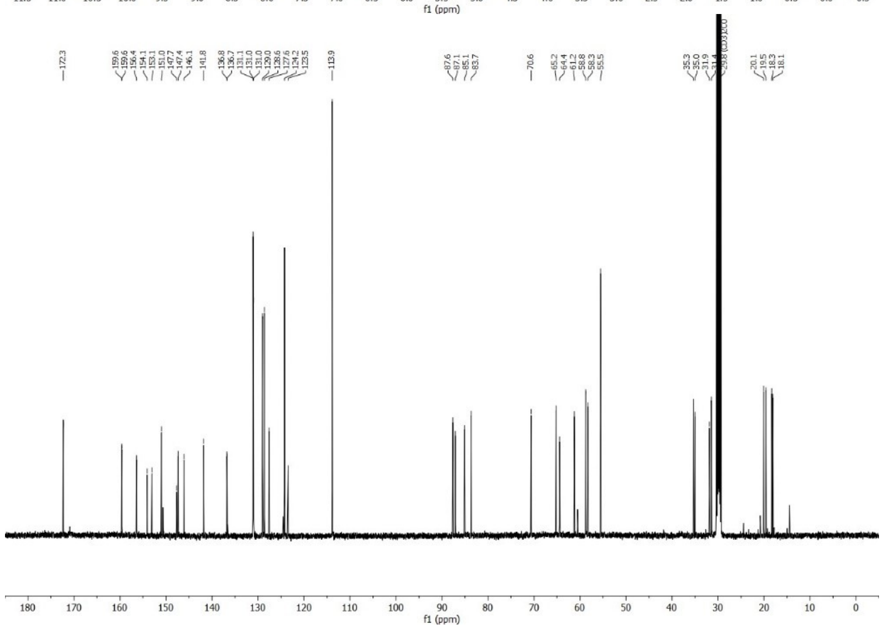

6_{DD}: Yield = 75%. Rf = 0.53 (1:1 iHex/Acetone). ^{1}H NMR (500 MHz with cryoprobe, acetone-d_6, 298 K): δ (ppm) = 10.79 (d, J = 8.0 Hz, 1H); 8.54 (s, 1H); 8.45 (s, 1H); 8.21 – 8.15 (m, 2H); 7.63 – 7.57 (m, 2H); 7.51 – 7.45 (m, 3H); 7.36 (dq, J = 8.6 Hz, J = 3.2 Hz, 4H); 7.31 – 7.24 (m, 2H); 7.24 – 7.17 (m, 1H); 6.89 – 6.81 (m, 4H); 6.26 (d, J = 4.2 Hz, 1H); 4.66 (dt, J = 6.4 Hz, J = 5.2 Hz, 1H); 4.60 (t, J = 4.6 Hz, 1H); 4.50 – 4.44 (m, 1H); 4.43 (q, J = 6.6 Hz, 2H); 4.36 (dd, J = 8.3 Hz, J = 5.8 Hz, 1H); 4.28 – 4.22 (m, 1H); 4.22 (d, J = 6.5 Hz, 1H); 3.93 (s, 3H); 3.77 (d, J = 1.4 Hz, 6H); 3.52 (s, 3H); 3.51 – 3.42 (m, 2H); 3.15 (t, J = 6.5 Hz, 2H); 2.25 (pd, J = 6.9 Hz, J = 5.2 Hz, 1H); 2.13 – 2.06 (m, 1H); 1.00 (d, J = 6.9 Hz, 6H); 0.87 (d, J = 6.9 Hz, 3H); 0.85 (d, J = 6.9 Hz, 3H). ^{13}C{^{1}H} NMR (125 MHz with cryoprobe, acetone-d_6, 298 K): δ (ppm) = 172.3; 172.1; 170.9; 159.6; 159.6; 156.3; 154.0; 153.1; 150.8; 147.7; 147.4; 146.1; 141.7; 136.8; 136.7; 131.1; 131.0; 131.0; 129.0; 128.6; 127.6; 124.3; 123.4; 113.9; 87.6; 87.1; 85.0; 83.7; 70.6; 65.2; 64.4; 60.9; 60.5; 58.8; 58.5; 55.5; 35.3; 35.0; 31.8; 31.3; 20.8; 20.0; 19.4; 18.3; 18.3; 14.5. FTIR ν_{max} (cm^{-1}): 2962 (w); 2932 (w); 1670 (m); 1576 (m); 1509 (s); 1464 (m); 1345 (m); 1250 (m); 1177 (m); 1033 (m). HRMS (ESI) m/z: [M+H]$^+$ Calcd for $C_{52}H_{61}O_{12}N_8$ 989.4403; Found 989.4426.

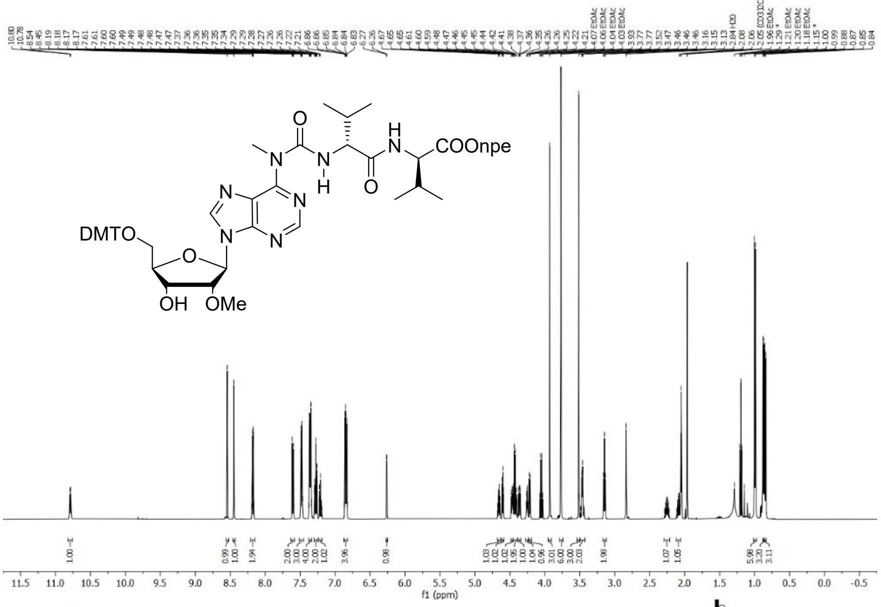

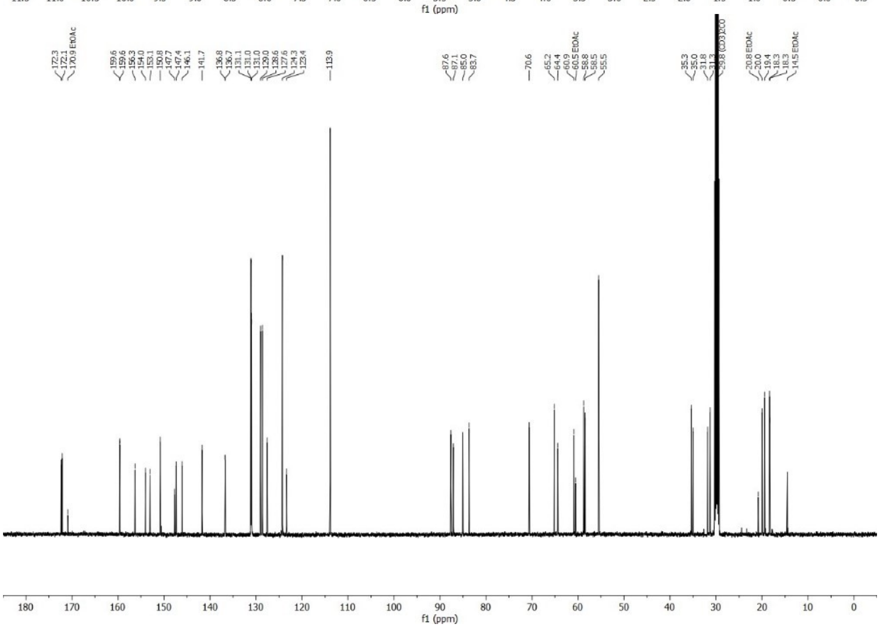

General procedure for the synthesis of compound 7: Compound **6** (1 equiv.) was added to a dry-oven round-bottom flask and dissolved in dry CH_2Cl_2. The solution was stirred under Argon atmosphere at 0°C. DIPEA (4 equiv.) was added dropwise. Finally, 2-cyanoethyl *N*,*N*-diisopropylchlorophosphoramidite (2.5 equiv.) was added dropwise. The reaction was stirred at r.t. for 3 h. After that, the reaction was stopped and diluted with CH_2Cl_2. The crude was washed with aqueous saturated $NaHCO_3$ and the organic layer was separated. The crude was further extracted with CH_2Cl_2. The combined organic layers were dried (Na_2SO_4), filtered and concentrated. The crude was purified by silica gel column chromatography (eluent containing 0.1% pyridine). The products were isolated as a mixture of diastereoisomers as a white foam. Finally, the product was lyophilized from benzene.

$\mathbf{7_{LD}}$**:** Yield = 70%. Rf = 0.34; 0.25 (2:3 iHex/EtOAc). $^{31}P\{^1H\}$ NMR (202 MHz with cryoprobe, acetone-d$_6$, 298 K): δ (ppm) = 150.3; 149.7. HRMS (ESI) *m/z*: $[M+H]^+$ Calcd for $C_{61}H_{78}O_{13}N_{10}P$ 1189.5481; Found 1189.5517.

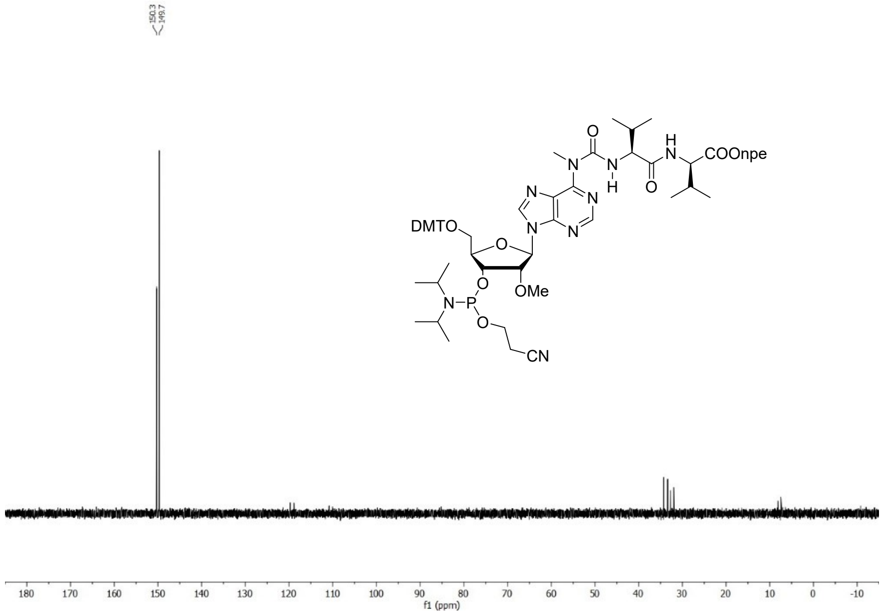

$\mathbf{7_{DL}}$**:** Yield = 51%. Rf = 0.40; 0.30 (2:3 iHex/EtOAc). $^{31}P\{^1H\}$ NMR (202 MHz with cryoprobe, acetone-d$_6$, 298 K): δ (ppm) = 150.3; 149.8. HRMS (ESI) *m/z*: $[M+H]^+$ Calcd for $C_{61}H_{78}O_{13}N_{10}P$ 1189.5481; Found 1189.5523.

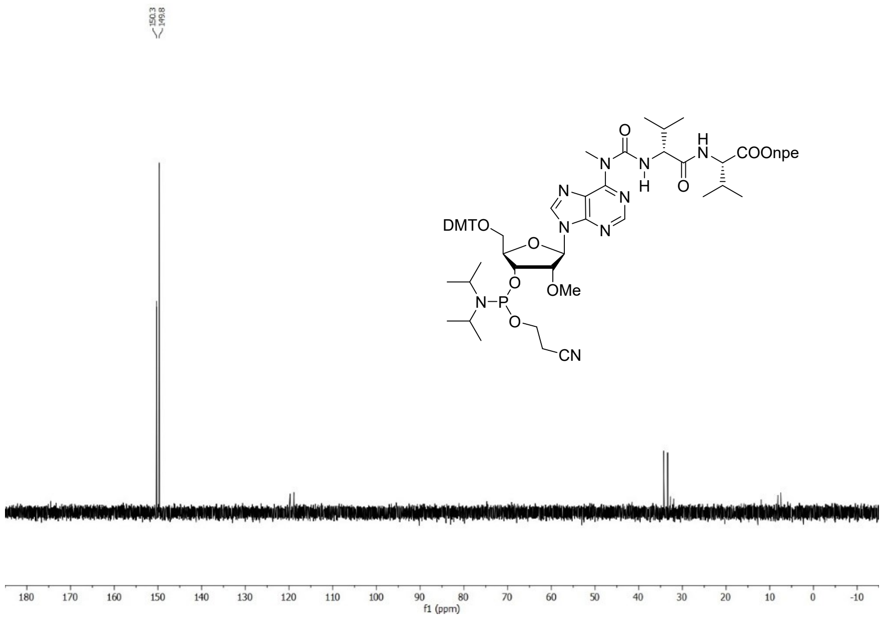

7$_{DD}$: Yield = 56%. Rf = 0.38; 0.28 (2:3 iHex/EtOAc). $^{31}P\{^1H\}$ NMR (202 MHz with cryoprobe, acetone-d$_6$, 298 K): δ (ppm) = 150.3; 149.8. HRMS (ESI) m/z: $[M+H]^+$ Calcd for $C_{61}H_{78}O_{13}N_{10}P$ 1189.5481; Found 1189.5506.

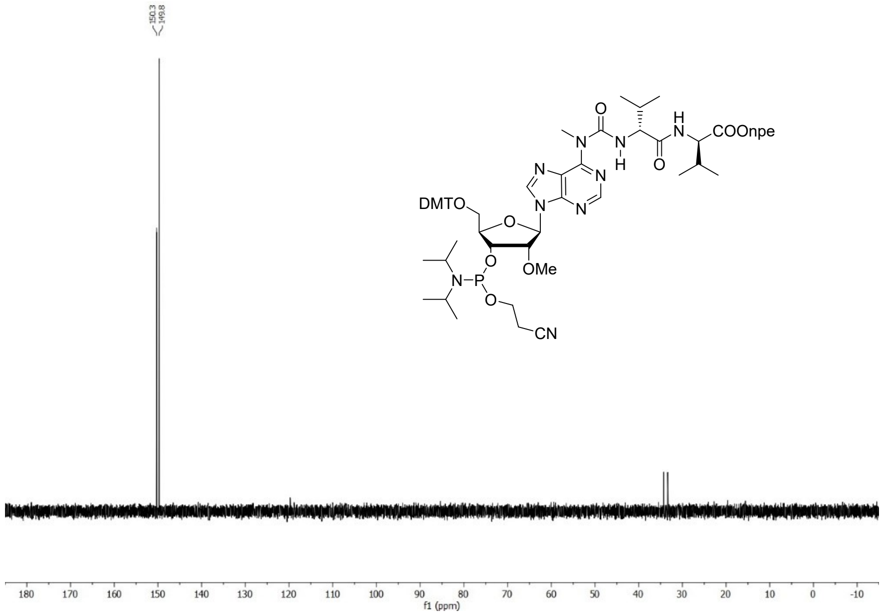

2.2 Amino acid-modified methyl N^6-carbamoyl deoxy adenosine phosphoramidites

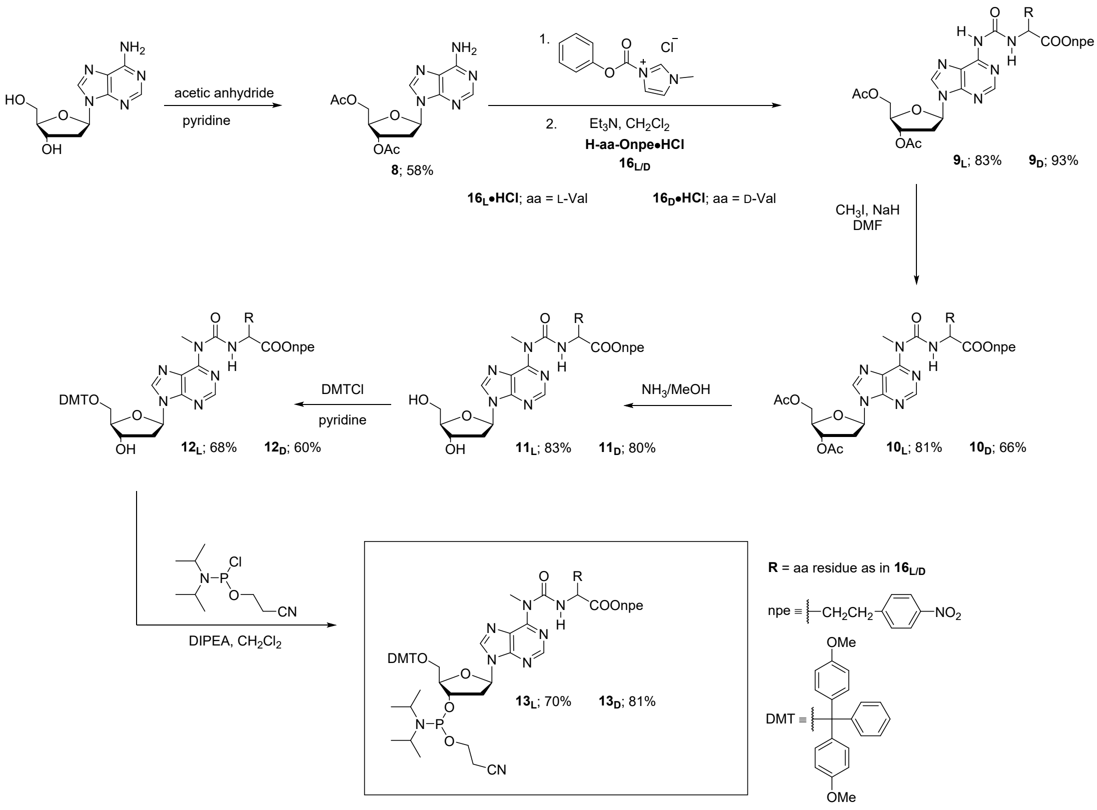

Scheme S2. Synthesis of amino acid-modified methyl N^6-carbamoyl deoxy adenosine phosphoramidites.

General procedure for the synthesis of compound 8: 2'-Deoxyadenosine (1 equiv.) was dissolved in dry pyridine and acetic anhydride (5 equiv.) was added dropwise. The reaction mixture was stirred for 2 h, then cooled down to 0°C and quenched with water. The volatiles were evaporated, and the residue was re-dissolved in CH_2Cl_2 and washed with aqueous saturated $NaHCO_3$. The aqueous phase was extracted twice with CH_2Cl_2, and the combined organic layers were dried (Na_2SO_4), filtered and concentrated. The crude was purified by silica gel column chromatography, followed by recrystallization from EtOH.

8: Yield = 58%. Rf = 0.38 (9:1 EtOAc/MeOH). ^{1}H NMR (500 MHz with cryoprobe, DMSO-d_6, 298 K): δ (ppm) = 8.34 (s, 1H); 8.15 (s, 1H); 7.34 (s, 2H); 6.37 (dd, J = 8.1 Hz, J = 6.2 Hz, 1H); 5.40 (dt, J = 6.4 Hz, J = 2.5 Hz, 1H); 4.32 (dd, J = 10.8 Hz, J = 3.8 Hz, 1H); 4.26 – 4.18 (m, 2H); 3.17 (ddd, J = 14.4 Hz, J = 8.2 Hz, J = 6.5 Hz, 1H); 2.56 – 2.51 (m, 1H); 2.09 (s, 3H); 2.01 (s, 3H). $^{13}C\{^{1}H\}$ NMR (125 MHz with cryoprobe, DMSO-d_6, 298 K): δ (ppm) = 170.4; 170.3; 156.3; 152.9; 149.4; 139.8; 119.5; 83.8; 81.8; 74.6; 63.8; 35.4; 21.0; 20.8. FTIR ν_{max} (cm^{-1}): 3306 (w); 3155 (w); 1733 (s); 1668 (s); 1602 (m); 1573 (m); 1507 (w); 1470 (m); 1362 (m); 1260 (m); 1156 (m); 1070 (m); 1041 (s); 975 (m); 939 (m); 906 (m); 857 (m); 837 (m); 799 (w); 748 (w); 691 (m); 649 (m); 638 (s). HRMS (ESI) m/z: $[M+H]^+$ Calcd for $C_{14}H_{18}O_5N_5$ 336.1302; Found 336.1303.

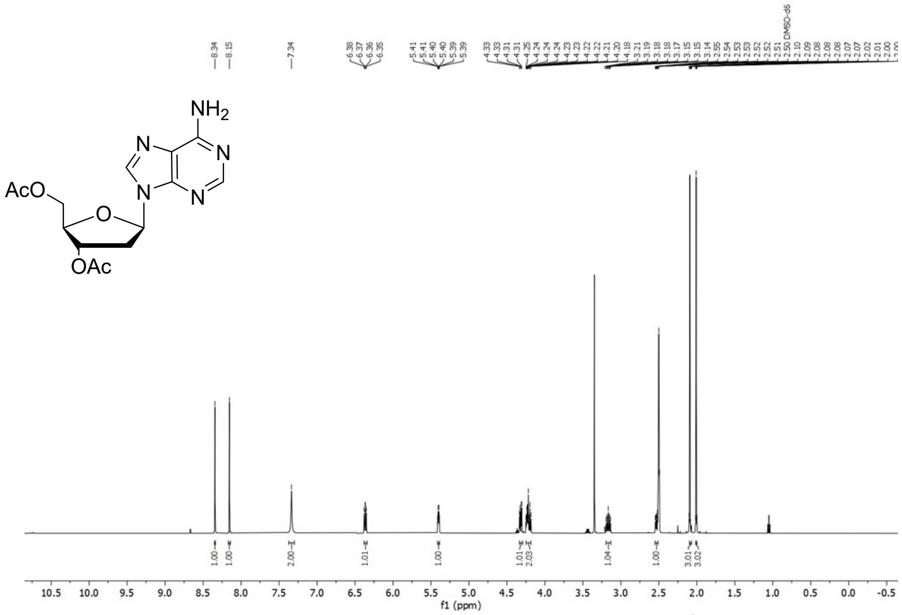

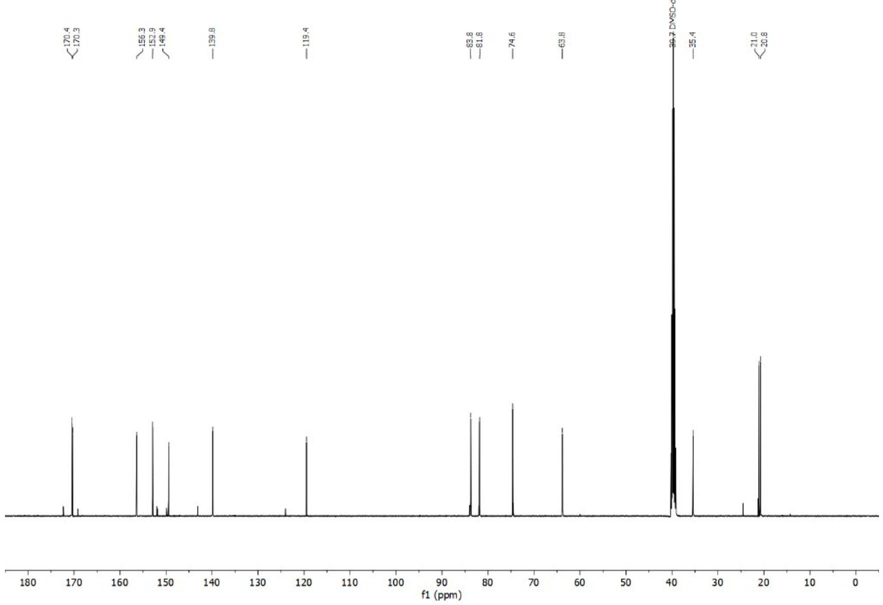

General procedure for the synthesis of compound 9: Step 1. Compound **8** (1 equiv.) and 1-*N*-methyl-3-phenoxycarbonyl-imidazolium chloride (2 equiv.) were added to an oven dried round-bottom flask and kept under vacuum for 15 min. After that, dry CH_2Cl_2 was added under nitrogen atmosphere and the reaction was stirred at r.t. for 5 h. Step 2. Onpe-protected amino acid **16•HCl** (2 equiv.) was added to an oven dried flask and suspended in dry CH_2Cl_2, followed by the addition of Et_3N (2 equiv.). The suspension was added dropwise to the reaction mixture. The reaction was stirred at r.t. under nitrogen atmosphere for 20 h. After that, the reaction was quenched with aqueous saturated $NaHCO_3$. The organic layer was separated and the crude was further extracted with CH_2Cl_2. The combined organic layers were dried (Na_2SO_4), filtered and concentrated. The crude was purified by silica gel column chromatography affording the product as a white foam.

9_L: Yield = 83%. Rf = 0.45 (10:0.5 EtOAc/MeOH). ^{1}H NMR (500 MHz with cryoprobe, DMSO-*d*$_6$, 298 K): δ (ppm) = 10.01 (s, 1H); 9.82 (d, *J* = 8.0 Hz, 1H); 8.65 (s, 1H); 8.56 (s, 1H); 8.18 – 7.92 (m, 2H); 7.64 – 7.44 (m, 2H); 6.47 (dd, *J* = 7.8 Hz, *J* = 6.3 Hz, 1H); 5.43 (dt, *J* = 6.5 Hz, *J* = 2.7 Hz, 1H); 4.44 (dt, *J* = 11.1 Hz, *J* = 6.3 Hz, 1H); 4.39 – 4.19 (m, 4H); 3.19 (ddd, *J* = 14.3 Hz, *J* = 8.0 Hz, *J* = 6.5 Hz, 1H); 3.06 (t, *J* = 6.4 Hz, 2H); 2.60 (ddd, *J* = 14.2 Hz, *J* = 6.3 Hz, *J* = 2.7 Hz, 1H); 2.10 (s, 3H); 2.00 (s, 3H); 0.90 (d, *J* = 6.8 Hz, 3H); 0.85 (d, *J* = 6.8 Hz, 3H). ^{13}C{^{1}H} NMR (125 MHz with cryoprobe, DMSO-*d*$_6$, 298 K): δ (ppm) = 171.5; 170.3; 170.2; 153.6; 150.9; 150.4; 150.4; 146.6; 146.3; 142.7; 130.3; 123.4; 120.7; 84.1; 82.1; 74.4; 64.4; 63.7; 58.4; 35.7; 34.2; 30.3; 20.9; 20.7; 19.2; 17.9. FTIR v_{max} (cm^{-1}): 2964 (w); 1737 (m); 1697 (m); 1609 (w); 1585 (m); 1517 (s); 1467 (m); 1345 (s); 1222 (s); 1107 (m); 1018 (m); 941 (m); 856 (m); 797 (w); 747 (w); 696 (m); 646 (m). HRMS (ESI) *m/z*: [M+H]$^+$ Calcd for $C_{28}H_{34}O_{10}N_7$ 628.2362; Found 628.2357.

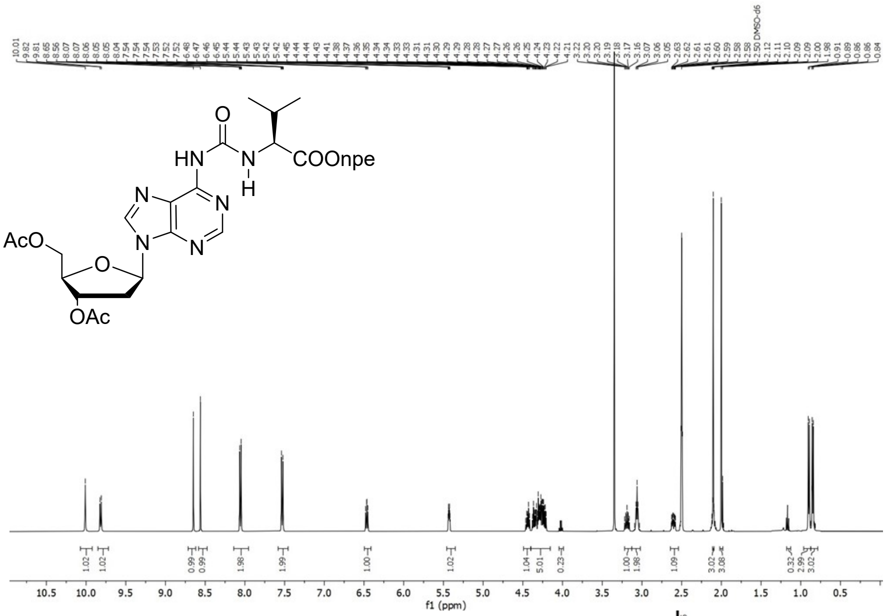

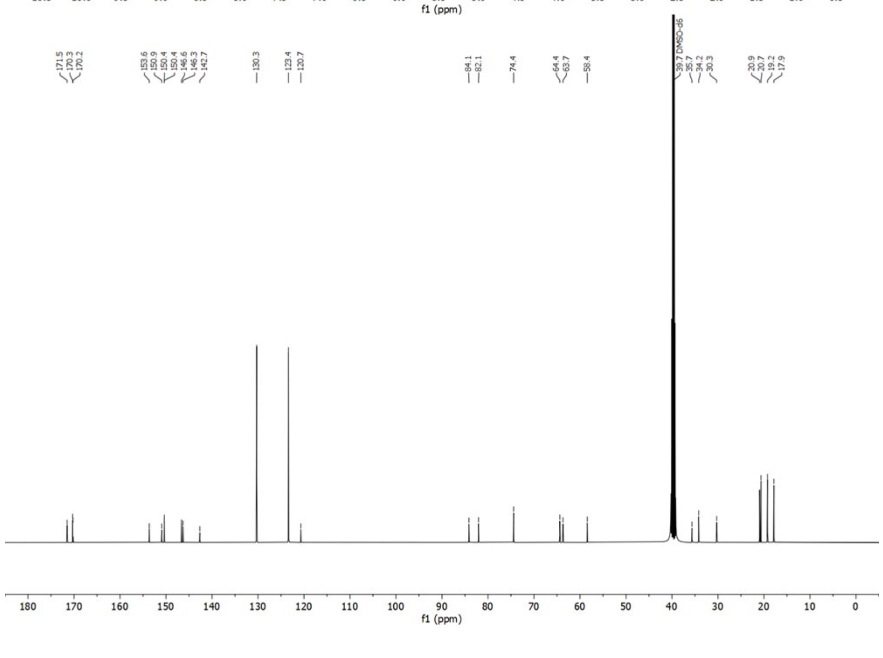

9_D: Yield = 93%. Rf = 0.45 (10:0.5 EtOAc/MeOH). ^{1}H NMR (500 MHz with cryoprobe, DMSO-d_6, 298 K): δ (ppm) = 10.01 (s, 1H); 9.83 (d, J = 8.1 Hz, 1H); 8.67 (s, 1H); 8.55 (s, 1H); 8.07 – 7.94 (m, 2H); 7.63 – 7.41 (m, 2H); 6.47 (dd, J = 7.9 Hz, J = 6.3 Hz, 1H); 5.43 (dt, J = 6.5 Hz, J = 2.5 Hz, 1H); 4.44 (dt, J = 11.0 Hz, J = 6.2 Hz, 1H); 4.40 – 4.20 (m, 5H); 3.16 (ddd, J = 14.3 Hz, J = 8.0 Hz, J = 6.5 Hz, 1H); 3.09 – 3.03 (m, 2H); 2.61 (ddd, J = 14.2 Hz, J = 6.3 Hz, J = 2.7 Hz, 1H); 2.10 (s, 3H); 2.00 (s, 3H); 0.90 (d, J = 6.8 Hz, 3H); 0.87 (s, 3H). ^{13}C{^{1}H} NMR (125 MHz with cryoprobe, DMSO-d_6, 298 K): δ (ppm) = 171.5; 170.3; 170.2; 153.6; 150.9; 150.4; 150.3; 146.6; 146.3; 142.4; 130.3; 123.3; 120.6; 83.8; 82.0; 74.4; 64.3; 63.7; 58.4; 35.8; 34.2; 30.3; 20.9; 20.7; 19.2; 17.9. FTIR ν_{max} (cm^{-1}): 2964 (w); 1737 (m); 1697 (m); 1609 (w); 1585 (m); 1517 (s); 1467 (m); 1345 (s); 1222 (s); 1107 (m); 1018 (m); 941 (m); 856 (m); 797 (w); 747 (w); 696 (m); 646 (m). HRMS (ESI) m/z: [M+H]$^+$ Calcd for $C_{28}H_{34}O_{10}N_7$ 628.2362; Found 628.2358.

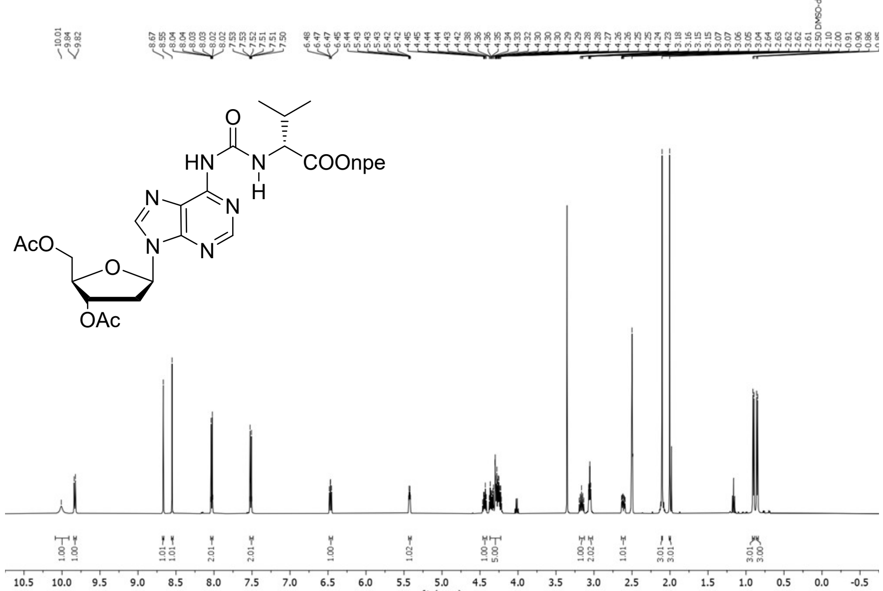

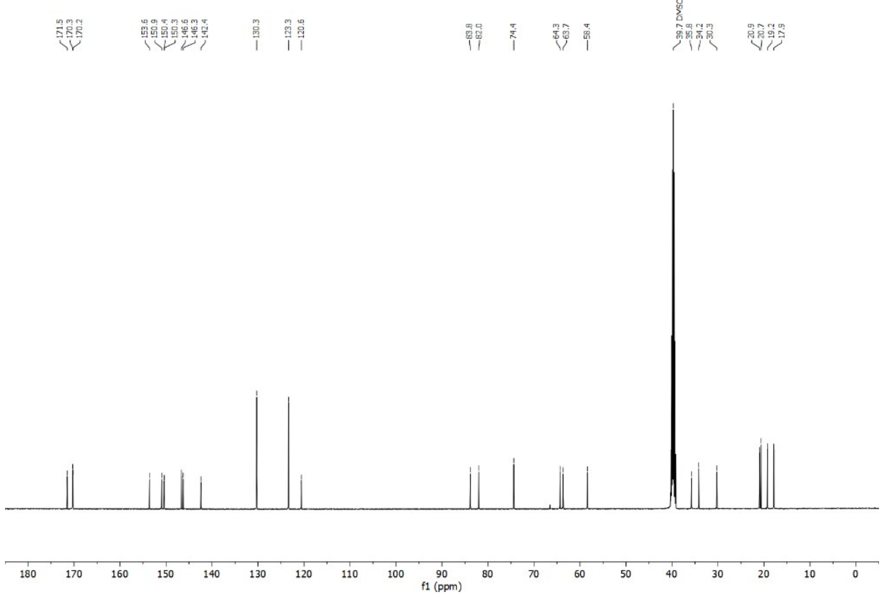

General procedure for the synthesis of compound 10: Compound **9** (1 equiv.) was dissolved in dry DMF under N_2 atmosphere. The solution was cooled to 0°C and NaH (1.05 equiv.) was added. The mixture was stirred at r.t. for 1 h. Then, MeI (1.05 equiv.) was added dropwise and the reaction was stirred for 1 h. The reaction mixture was diluted with EtOAc and washed with water. The combined organic layers were dried (Na_2SO_4), filtered and concentrated. The crude was purified by silica gel column chromatography affording the product as a white foam.

10_L: Yield = 81%. Rf = 0.50 (5:1 EtOAc/iHex). ^{1}H NMR (500 MHz with cryoprobe, DMSO-d_6, 298 K): δ (ppm) = 10.76 (d, J = 7.5 Hz, 1H); 8.68 (s, 1H); 8.59 (s, 1H); 8.08 – 8.01 (m, 2H); 7.53 – 7.48 (m, 2H); 6.50 (dd, J = 7.9 Hz, J = 6.3 Hz, 1H); 5.42 (dq, J = 7.0 Hz, J = 2.3 Hz, 1H); 4.38 – 4.27 (m, 4H); 4.26 – 4.20 (m, 2H); 3.79 (s, 3H); 3.18 – 3.10 (m, 1H); 3.05 (t, J = 6.3 Hz, 2H); 2.61 (dddd, J = 14.2 Hz, J = 6.4 Hz, J = 2.8 Hz, J = 1.3 Hz, 2H); 2.11 (s, 3H); 2.01 (s, 3H); 0.89 (d, J = 6.8 Hz, 3H); 0.85 (d, J = 6.9 Hz, 3H). ^{13}C{^{1}H} NMR (125 MHz with cryoprobe, DMSO-d_6, 298 K): δ (ppm) = 171.6; 170.4; 170.3; 155.3; 152.5; 152.0; 150.0; 141.7; 130.3; 123.4; 122.1; 83.9; 82.1; 74.5; 63.8; 59.5; 52.1; 35.7; 34.4; 34.2; 30.2; 21.0; 20.7; 19.4; 18.1. FTIR ν_{max} (cm^{-1}): 1735 (m); 1682 (m); 1572 (m); 1517 (s); 1465 (m); 1345 (s); 1218 (s); 1182 (s); 1106 (m); 1049 (m); 1018 (m); 942 (m); 856 (m); 797 (m); 747 (m); 698 (m); 646 (m). HRMS (ESI) m/z: [M+H]$^+$ Calcd for $C_{29}H_{36}O_{10}N_7$ 642.2518; Found 642.2515.

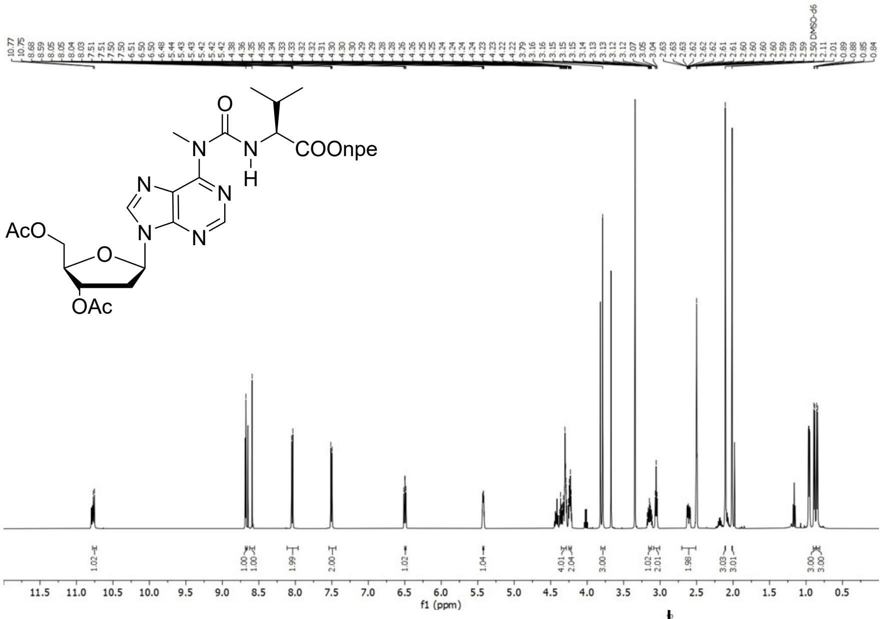

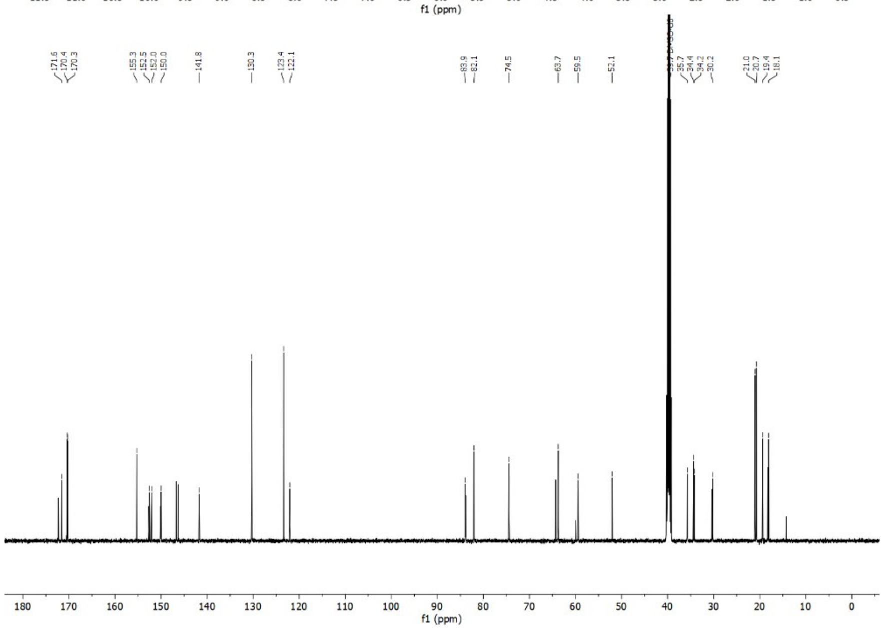

10_D: Yield = 66%. Rf = 0.53 (5:1 EtOAc/iHex). ^{1}H NMR (500 MHz with cryoprobe, DMSO-d_6, 298 K): δ (ppm) = 10.75 (d, J = 7.7 Hz, 1H); 8.68 (s, 1H); 8.58 (s, 1H); 8.06 – 7.98 (m, 2H); 7.53 – 7.45 (m, 2H); 6.50 (dd, J = 7.9 Hz, J = 6.2 Hz, 1H); 5.42 (dt, J = 6.5 Hz, J = 2.6 Hz, 1H); 4.46 – 4.27 (m, 5H); 4.27 – 4.20 (m, 2H); 3.79 (s, 3H); 3.13 (ddd, J = 14.4 Hz, J = 8.1 Hz, J = 6.5 Hz, 1H); 3.05 (t, J = 6.3 Hz, 2H); 2.62 (ddd, J = 14.2 Hz, J = 6.3 Hz, J = 2.7 Hz, 1H); 2.11 (s, 3H); 2.01 (s, 3H); 0.90 (d, J = 6.8 Hz, 3H); 0.86 (d, J = 6.8 Hz, 3H). ^{13}C{^{1}H} NMR (125 MHz with cryoprobe, DMSO-d_6, 298 K): δ (ppm) = 171.5; 170.3; 170.3; 155.3; 152.5; 152.1; 150.0; 146.7; 146.3; 141.6; 130.3; 123.3; 122.0; 83.7; 82.0; 74.4; 64.2; 63.7; 59.5; 35.8; 34.4; 34.2; 30.2; 20.9; 20.7; 19.3; 18.1. FTIR ν_{max} (cm^{-1}): 2964 (w); 1737 (m); 1685 (m); 1517 (s); 1467 (m); 1345 (s); 1221 (s); 1184 (m); 1107 (m); 1018 (m); 941 (m); 856 (m); 797 (w); 747 (m); 696 (m); 646 (m). HRMS (ESI) m/z: $[M+H]^+$ Calcd for $C_{29}H_{36}O_{10}N_7$ 642.2518; Found 642.2515.

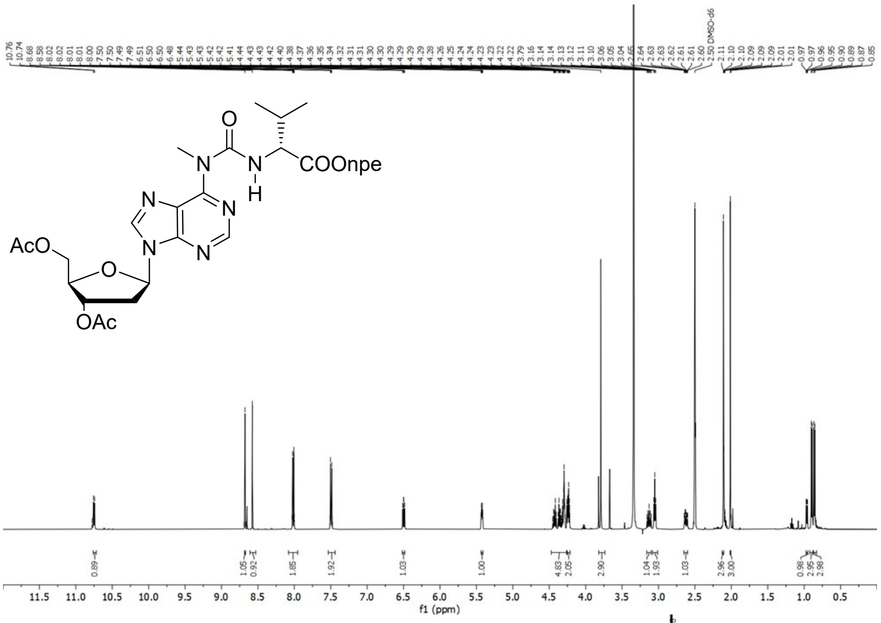

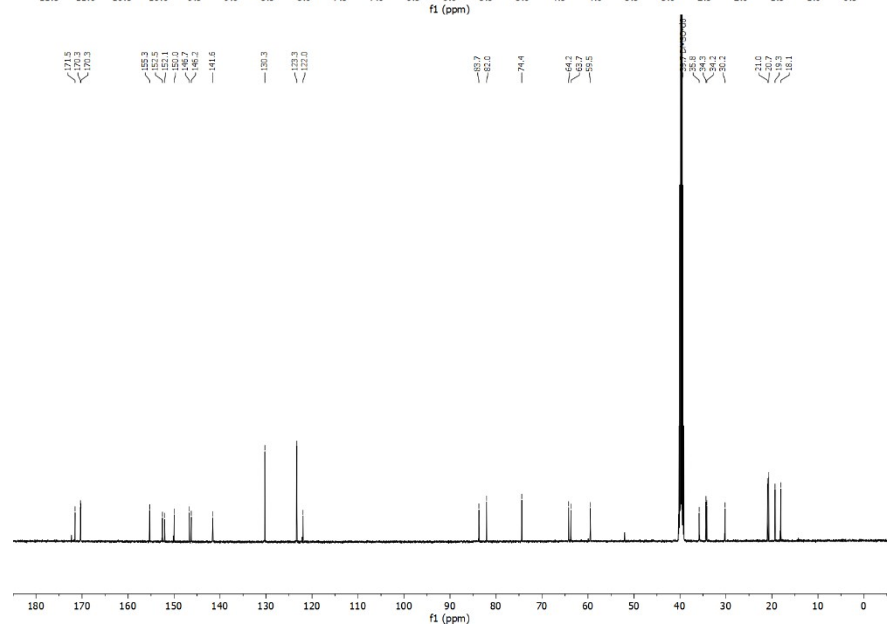

General procedure for the synthesis of compound 11: Compound **10** (1 equiv.) was diluted with 7 N NH_3/MeOH and stirred at r.t. for 2 h. Then, the reaction mixture was concentrated and the crude was purified by silica gel column chromatography.

11_L: Yield = 83%. Rf = 0.46 (10:0.75 CH_2Cl_2/MeOH). ^{1}H NMR (500 MHz with cryoprobe, DMSO-d_6, 298 K): δ (ppm) = 9.92 (s, 1H); 8.63 (s, 1H); 8.56 (s, 1H); 8.11 – 8.01 (m, 2H); 7.56 – 7.45 (m, 2H); 6.47 (dd, J = 7.8 Hz, J = 6.3 Hz, 1H); 5.49 – 5.39 (m, 1H); 4.44 (dt, J = 11.0 Hz, J = 6.3 Hz, 1H); 4.39 – 4.20 (m, 5H); 3.19 (ddd, J = 14.3 Hz, J = 7.8 Hz, J = 6.5 Hz, 1H); 3.07 (t, J = 6.4 Hz, 2H); 2.65 – 2.56 (m, 1H); 2.10 (s, 4H); 2.00 (s, 3H); 0.90 (d, J = 6.8 Hz, 3H); 0.86 (d, J = 6.8 Hz, 3H). ^{13}C{^{1}H} NMR (125 MHz with cryoprobe, DMSO-d_6, 298 K): δ (ppm) = 171.5; 170.3; 170.2; 153.6; 150.9; 150.4; 146.6; 146.3; 142.6; 130.3; 123.4; 120.7; 84.1; 82.0; 74.4; 64.4; 63.7; 58.4; 35.6; 34.2; 30.2; 20.9; 20.7; 19.3; 17.9. FTIR ν_{max} (cm^{-1}): 1735 (w); 1678 (w); 1572 (m); 1516 (s); 1465 (m); 1343 (s); 1261 (m); 1182 (m); 1050 (m); 1017 (m); 941 (m); 856 (m); 796 (m); 747 (m); 696 (m); 646 (m). HRMS (ESI) m/z: [M+H]$^+$ Calcd for $C_{25}H_{32}O_8N_7$ 558.2307; Found 558.2321.

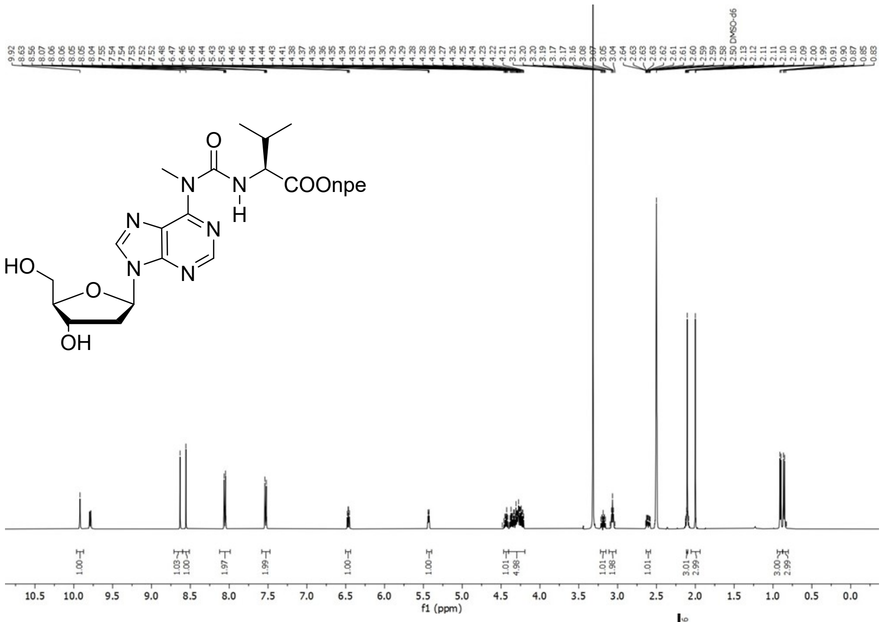

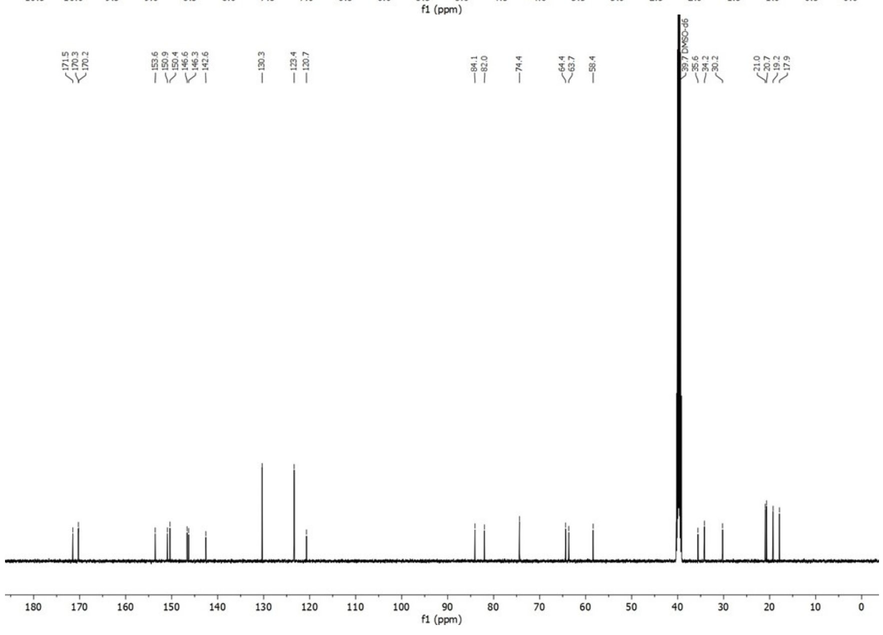

11_D: Yield = 80%. Rf = 0.47 (10:0.75 CH_2Cl_2/MeOH). ^{1}H NMR (500 MHz with cryoprobe, DMSO-d_6, 298 K): δ (ppm) = 10.80 (d, J = 7.5 Hz, 1H); 8.70 (s, 1H); 8.56 (s, 1H); 8.04 – 7.96 (m, 2H); 7.54 – 7.45 (m, 2H); 6.47 (t, J = 6.7 Hz, 1H); 5.39 (d, J = 4.2 Hz, 1H); 5.03 (t, J = 5.5 Hz, 1H); 4.47 – 4.30 (m, 3H); 4.23 (dd, J = 7.6 Hz, J = 4.9 Hz, 1H); 3.90 (td, J = 4.4 Hz, J = 3.0 Hz, 1H); 3.79 (s, 3H); 3.62 (dt, J = 11.7 Hz, J = 5.0 Hz, 1H); 3.54 (ddd, J = 11.7 Hz, J = 5.6 Hz, J = 4.4 Hz, 1H); 3.05 (t, J = 6.3 Hz, 2H); 2.71 (ddd, J = 13.1 Hz, J = 7.2 Hz, J = 5.9 Hz, 1H); 2.38 (ddd, J = 13.3 Hz, J = 6.3 Hz, J = 3.6 Hz, 1H); 2.09 (pd, J = 6.8 Hz, J = 5.0 Hz, 1H); 0.89 (d, J = 6.9 Hz, 3H); 0.85 (d, J = 6.9 Hz, 3H). ^{13}C{^{1}H} NMR (125 MHz with cryoprobe, DMSO-d_6, 298 K): δ (ppm) = 171.8; 155.4; 152.6; 150.1; 147.0; 146.5; 141.8; 130.6; 123.6; 122.1; 88.4; 84.0; 71.0; 64.5; 61.9; 59.8; 34.6; 34.4; 30.5; 19.7; 18.4. FTIR ν_{max} (cm^{-1}): 1735 (w); 1678 (w); 1572 (m); 1516 (s); 1465 (m); 1421 (m); 1343 (s); 1263 (m); 1184 (m); 1050 (m); 1017 (m); 941 (m); 856 (m); 796 (m); 747 (m); 696 (m); 646 (m). HRMS (ESI) m/z: [M+H]$^+$ Calcd for $C_{25}H_{32}O_8N_7$ 558.2307; Found 558.2321.

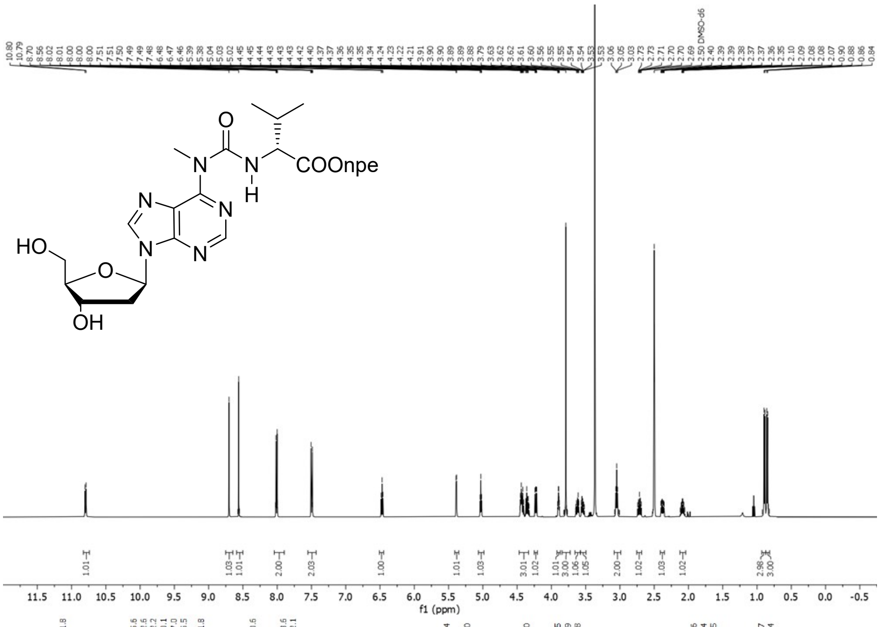

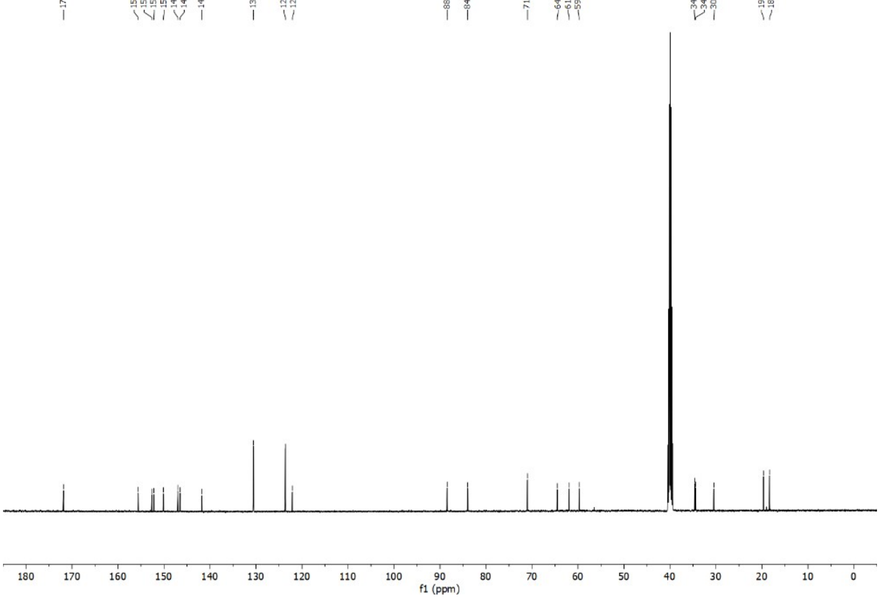

General procedure for the synthesis of compound 12: Compound **11** (1 equiv.) was dissolved in dry pyridine and stirred under nitrogen atmosphere at r.t. 4,4-Dimethoxytrityl chloride (1.5 equiv.) was added in two portions and the reaction was stirred at r.t. overnight. After that, the crude was concentrated and purified by silica gel column chromatography (eluent containing 0.1% pyridine) affording the product as a colourless foam.

12_L: Yield = 68%. Rf = 0.39 (10:0.5 CH_2Cl_2/MeOH). ^{1}H NMR (500 MHz with cryoprobe, DMSO-d_6, 298 K): δ (ppm) = 10.80 (d, J = 7.6 Hz, 1H); 8.52 (d, J = 36.5 Hz, 2H); 8.04 (d, J = 8.3 Hz, 2H); 7.50 (d, J = 8.2 Hz, 2H); 7.32 (d, J = 7.6 Hz, 2H); 7.26 – 7.12 (m, 7H); 6.78 (dd, J = 19.0 Hz, J = 8.4 Hz, 4H); 6.49 (t, J = 6.4 Hz, 1H); 5.42 (s, 1H); 4.56 – 4.20 (m, 4H); 4.06 (t, J = 4.6 Hz, 1H); 3.80 (s, 3H); 3.69 (d, J = 6.6 Hz, 6H); 3.26 – 3.14 (m, 2H); 3.05 (t, J = 6.4 Hz, 2H); 2.91 (dt, J = 13.0 Hz, J = 6.1 Hz, 1H); 2.41 (dt, J = 12.7 Hz, J = 5.9 Hz, 1H); 2.10 (dq, J = 13.6 Hz, J = 6.6 Hz, 1H); 0.90 (d, J = 6.8 Hz, 3H); 0.86 (d, J = 7.2 Hz, 3H). ^{13}C{^{1}H} NMR (125 MHz with cryoprobe, DMSO-d_6, 298 K): δ (ppm) = 171.5; 158.2; 155.3; 152.4; 151.9; 149.6; 146.6; 146.3; 145.1; 141.9; 135.7; 130.2; 129.8; 127.8; 126.7; 123.3; 122.1; 113.2; 86.3; 85.6; 84.1; 70.8; 64.3; 59.4; 55.1; 55.0; 34.3; 34.2; 30.2; 19.3; 18.0. FTIR v_{max} (cm^{-1}): 2966 (w); 1731 (w); 1685 (w); 1606 (w); 1575 (s); 1518 (s); 1510 (s); 1464 (m); 1421 (w); 1391 (w); 1345 (s); 1299 (m); 1250 (s); 1218 (m); 1178 (s); 1151 (m); 1109 (m); 1073 (m); 1060 (m); 1033 (s); 1005 (m); 977 (m); 952 (m); 902 (w); 852 (w); 827 (m); 797 (m); 768 (m); 748 (m); 728 (m); 699 (m); 649 (m). HRMS (ESI) m/z: $[M+H]^+$ Calcd for $C_{46}H_{50}O_{10}N_7$ 860.3614; Found 860.3643.

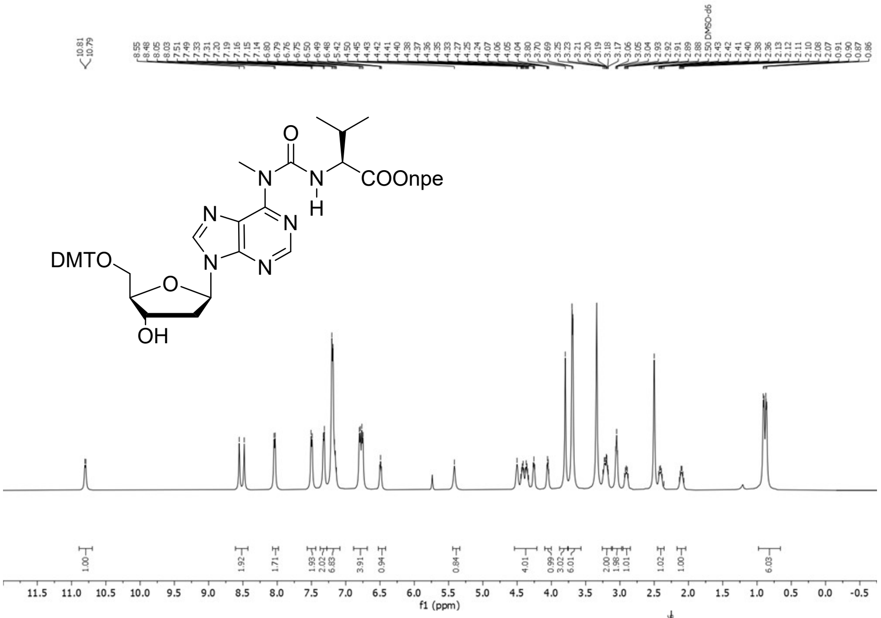

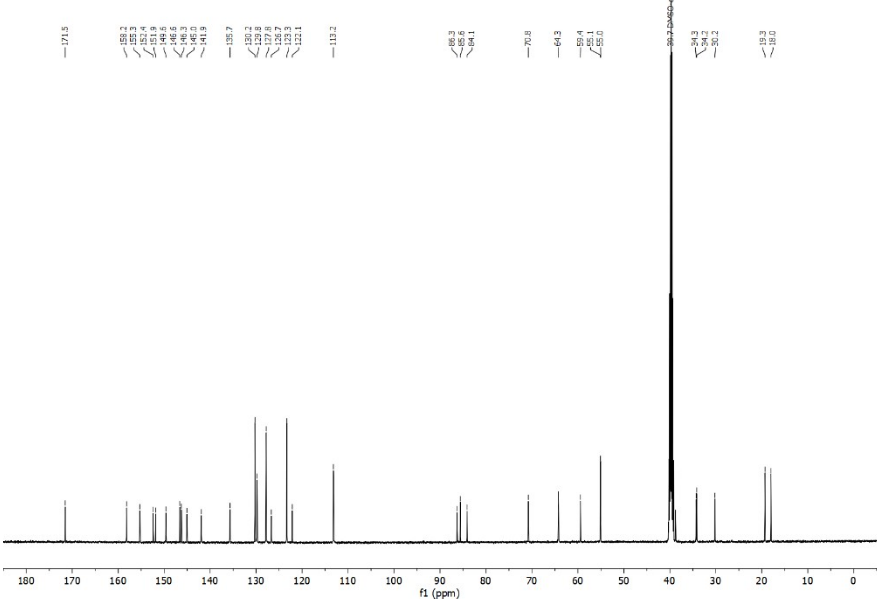

12_D: Yield = 60%. Rf = 0.46 (10:0.75 CH_2Cl_2/MeOH). ^{1}H NMR (500 MHz with cryoprobe, DMSO-d_6, 298 K): δ (ppm) = 10.80 (d, J = 7.5 Hz, 1H); 8.58 (s, 1H); 8.51 (s, 1H); 8.07 – 8.00 (m, 2H); 7.54 – 7.46 (m, 2H); 7.34 – 7.27 (m, 2H); 7.27 – 7.11 (m, 11H); 6.83 – 6.71 (m, 4H); 6.49 (t, J = 6.2 Hz, 1H); 5.44 (d, J = 4.6 Hz, 1H); 4.52 – 4.39 (m, 2H); 4.35 (dt, J = 11.0 Hz, J = 6.1 Hz, 1H); 4.23 (dd, J = 7.6 Hz, J = 4.9 Hz, 1H); 4.02 (dt, J = 6.0 Hz, J = 4.1 Hz, 1H); 3.78 (s, 3H); 3.69 (d, J = 5.9 Hz, 6H); 3.17 (h, J = 6.1 Hz, 2H); 3.05 (t, J = 6.3 Hz, 2H); 2.92 – 2.83 (m, 1H); 2.41 (ddd, J = 13.4 Hz, J = 6.9 Hz, J = 5.1 Hz, 1H); 2.29 (s, 2H); 2.09 (pd, J = 6.9 Hz, J = 4.9 Hz, 1H); 0.87 (dd, J = 17.3 Hz, J = 6.8 Hz, 6H). ^{13}C{^{1}H} NMR (125 MHz with cryoprobe, DMSO-d_6, 298 K): δ (ppm) = 171.6; 158.2; 155.3; 152.4; 149.8; 146.8; 145.11; 141.8; 137.6; 135.7; 130.3; 129.8; 129.1; 128.4; 127.8; 126.8; 125.6; 123.4; 122.1; 113.2; 86.2; 85.6; 83.8; 70.7; 64.3; 59.5; 55.2; 34.4; 30.2; 21.3; 19.4; 18.1. FTIR ν_{max} (cm^{-1}): 1735 (m); 1606 (m); 1465 (m); 1345 (s); 1300 (m); 1248 (s); 1217 (s); 1175 (s); 1028 (s); 1028 (m); 944 (m); 856 (m); 827 (m); 797 (m); 748 (m); 698 (m); 648 (m). HRMS (ESI) m/z: [M+H]$^+$ Calcd for $C_{46}H_{50}O_{10}N_7$ 860.3614; Found 860.3637.

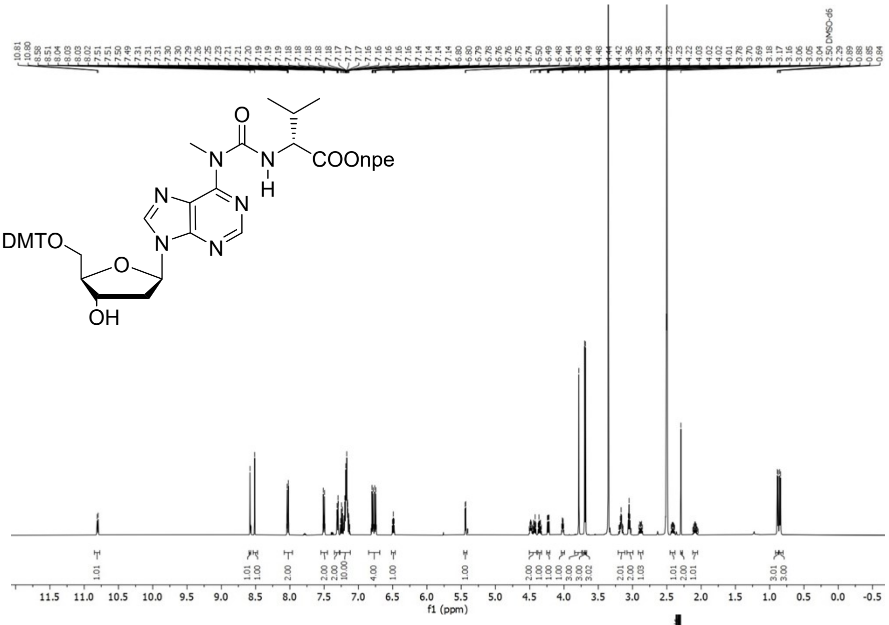

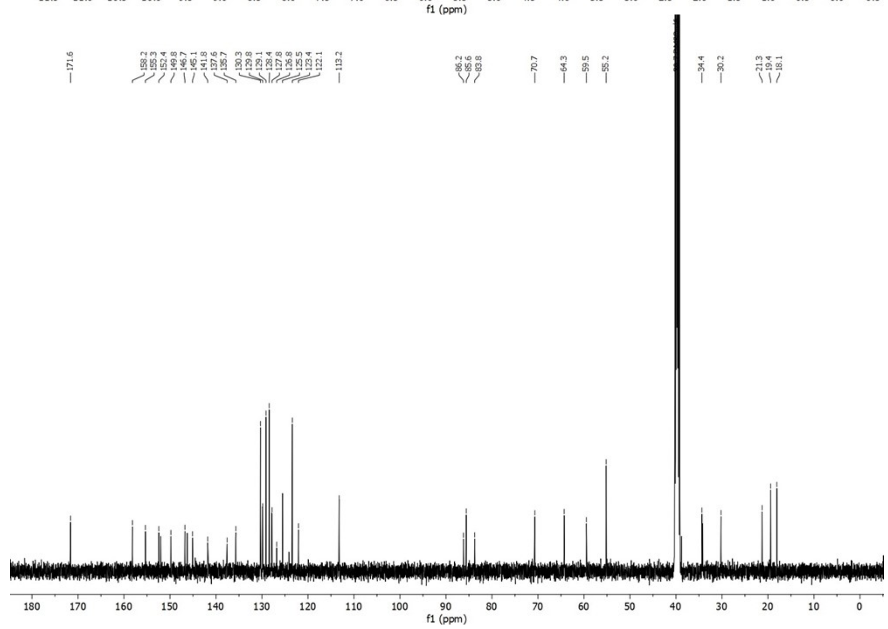

General procedure for the synthesis of compound 13: Compound **12** (1 equiv.) was added to a dry-oven flask and dissolved in dry CH_2Cl_2. The solution was stirred under Argon atmosphere at 0°C. DIPEA (4 equiv.) was added dropwise. Finally, 2-cyanoethyl *N*,*N*-diisopropylchlorophosphoramidite (2.5 equiv.) was added dropwise. The reaction was stirred at r.t. for 3 h. After that, the reaction was stopped and diluted with CH_2Cl_2. The crude was washed with aqueous saturated $NaHCO_3$ and the organic layer was separated. The crude was further extracted with CH_2Cl_2. The combined organic layers were dried (Na_2SO_4), filtered and concentrated. The crude was purified by silica gel column chromatography (eluent containing 0.1% pyridine). The products were isolated as a mixture of diastereoisomers as a white foam. Finally, the product was lyophilized from benzene.

13_L: Yield = 70%. Rf = 0.70 (1:5 iHex/EtOAc). $^{31}P\{^1H\}$ NMR (202 MHz with cryoprobe, acetone-d_6, 298 K): δ (ppm) = 148.3; 148.2. HRMS (ESI) *m/z*: $[M+H]^+$ Calcd for $C_{55}H_{67}N_9O_{11}P$ 1060.4692; Found 1060.4742.

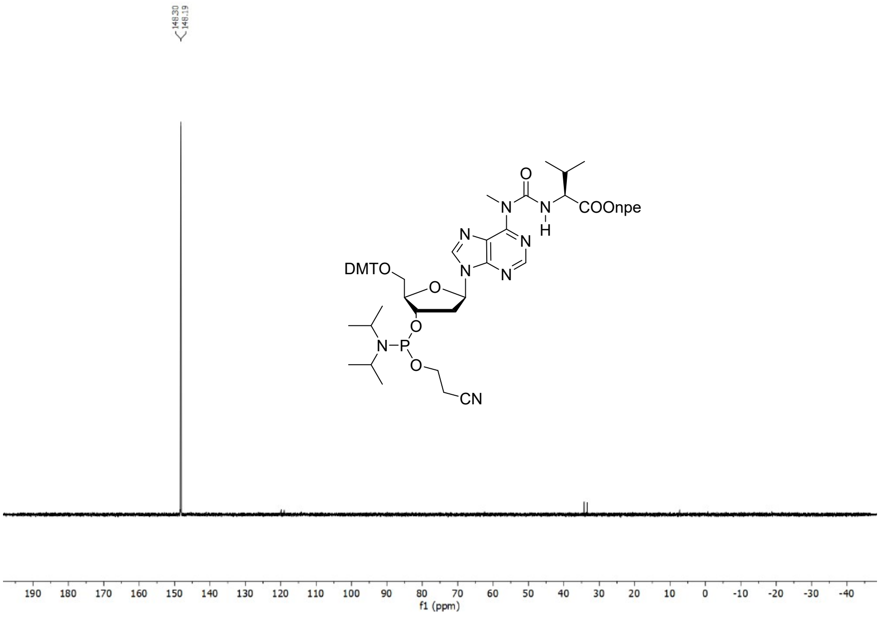

13_D: Yield = 81%. Rf = 0.70 (1:5 iHex/EtOAc). $^{31}P\{^1H\}$ NMR (202 MHz with cryoprobe, acetone-d_6, 298 K): δ (ppm) = 150.3; 149.8. HRMS (ESI) *m/z*: $[M+H]^+$ Calcd for $C_{55}H_{67}N_9O_{11}P$ 1060.4692; Found 1060.4751.

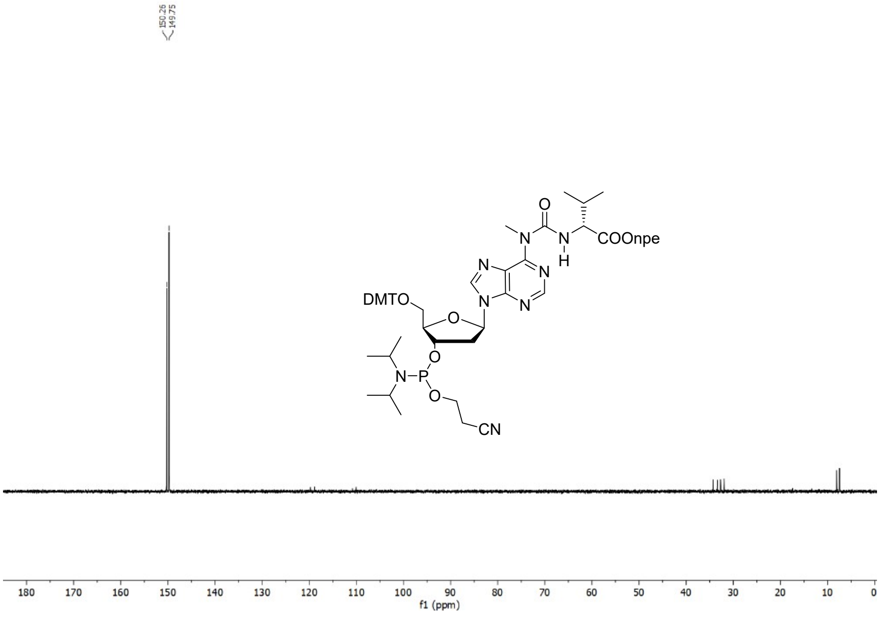

2.3 Npe-protected amino acids

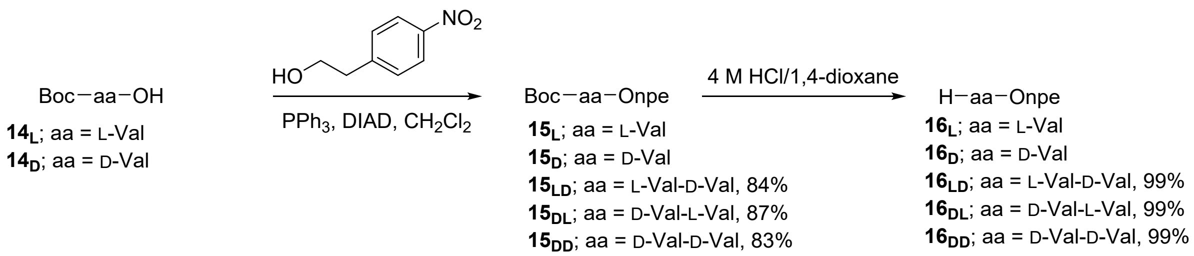

Scheme S3. Synthesis of Onpe-protected amino acids **16•HCl**.

Compounds $\mathbf{14_L}$, $\mathbf{14_D}$ are commercially available.

The Boc- and Onpe-protected amino acids $\mathbf{15_L}$ and $\mathbf{15_D}$ and their corresponding Onpe-deprotected derivatives $\mathbf{16_L}$**•HCl** and $\mathbf{16_D}$**•HCl** were synthesized following a procedure previously reported in the literature.[2,3]

General procedure for the synthesis of peptides: Compound $\mathbf{16_L}$ or $\mathbf{16_D}$ were dissolved in dry CH_2Cl_2 and compound $\mathbf{14_L}$ or $\mathbf{14_D}$ was added. The mixture was stirred at 0°C in an ice bath. 2-(1*H*-Benzotriazol-1-yl)-1,1,3,3-tetramethyluronium-hexafluorophosphat (HBTU) and diisopropylethylamine (DIPEA) were added and the reaction was stirred at r.t. for another 2 h. The reaction was quenched with aqueous saturated NH_4Cl and extracted with CH_2Cl_2. The combined organic layers were dried ($MgSO_4$), filtered and concentrated. The crude was purified by silica gel column chromatography affording the product as a white solid.

$\mathbf{15_{LD}}$**:** Yield = 84%. Rf = 0.30 (7:3 iHex/EtOAc). ^{1}H NMR (500 MHz with cryoprobe, $CDCl_3$, 298 K): δ (ppm) = 8.19 – 8.14 (m, 2H); 7.41 – 7.36 (m, 2H); 6.47 (d, J = 8.7 Hz, 1H); 5.03 – 4.98 (m, 1H); 4.48 (dd, J = 8.7 Hz, J = 4.9 Hz, 1H); 4.41 – 4.36 (m, 2H); 3.96 (t, J = 7.1 Hz, 1H); 3.06 (t, J = 6.9 Hz, 2H); 2.19 (pd, J = 6.9 Hz, J = 5.4 Hz, 1H); 2.08 (pd, J = 6.9 Hz, J = 4.9 Hz, 1H); 1.43 (s, 9H); 0.96 (d, J = 6.8 Hz, 3H); 0.88 (dd, J = 6.9 Hz, J = 6.0 Hz, 6H); 0.79 (d, J = 6.9 Hz, 3H). ^{13}C{^{1}H} NMR (125 MHz with cryoprobe, $CDCl_3$, 298 K): δ (ppm) = 171.8; 171.7; 147.1; 145.4; 129.9; 123.9; 64.7; 57.1; 34.9; 31.1; 30.7; 28.4; 19.5; 19.1; 17.7. FTIR ν_{max} (cm^{-1}): 3314 (w); 2972 (w); 1729 (m); 1656 (m); 1519 (s); 1347 (s); 1246 (m); 1163 (m). HRMS (ESI) m/z: [M+Na]$^+$ Calcd for $C_{23}H_{35}O_7N_3Na$ 488.2367; Found 488.2377.

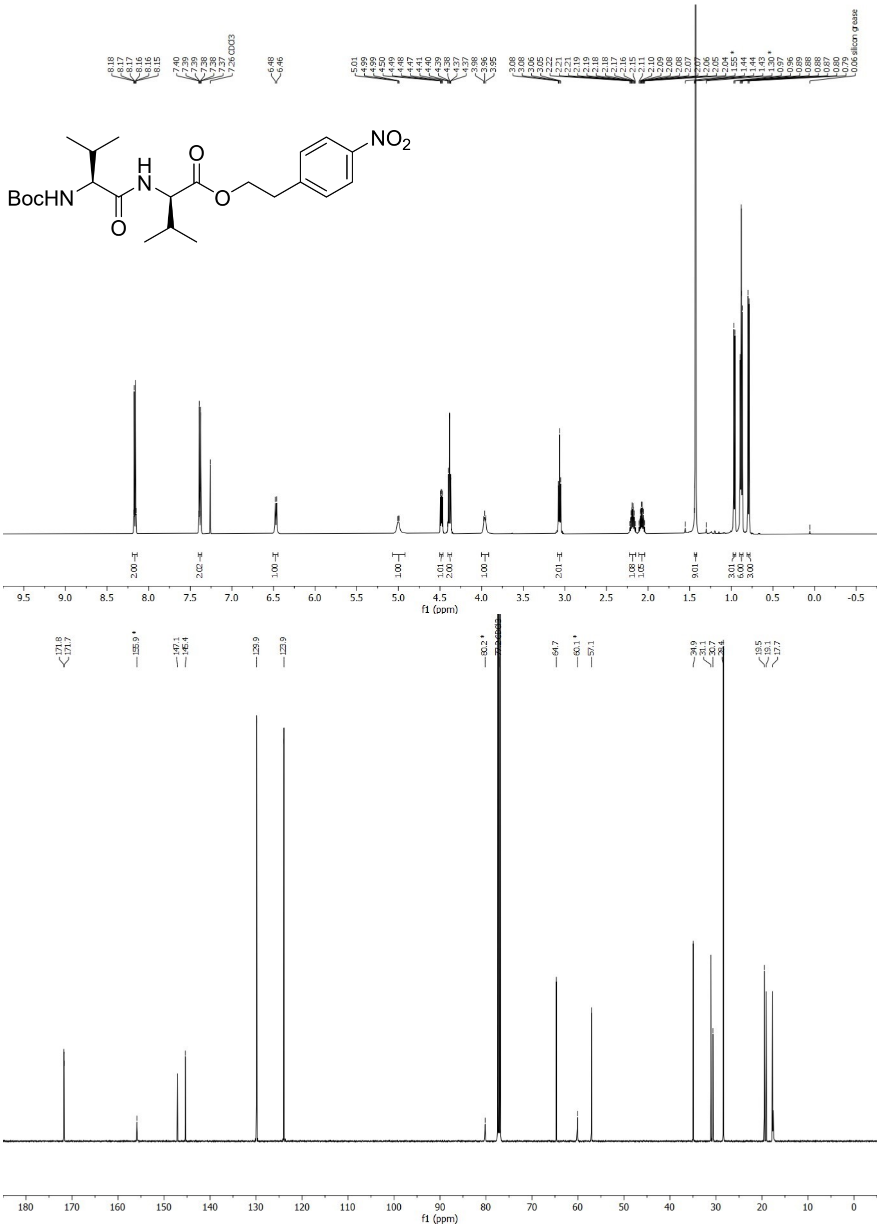

15_{DL}: Yield = 87%. Rf = 0.31 (7:3 iHex/EtOAc). ^{1}H NMR (500 MHz with cryoprobe, $CDCl_3$, 298 K): *δ* (ppm) = 8.17 – 8.14 (m, 2H); 7.39 – 7.36 (m, 2H); 6.42 (d, *J* = 8.6 Hz, 1H); 5.05 (d, *J* = 8.8 Hz, 1H); 4.46 (dd, *J* = 8.6 Hz, *J* = 5.0 Hz, 1H); 4.38 (ddt, *J* = 11.0 Hz, *J* = 6.8 Hz, *J* = 4.3 Hz, 2H); 3.88 (dd, *J* = 8.8 Hz, *J* = 6.5 Hz, 1H); 3.06 (t, *J* = 6.9 Hz, 2H); 2.14 – 2.04 (m, 2H); 1.42 (s, 9H); 0.91 (dd, *J* = 11.2 Hz, *J* = 6.8 Hz, 6H); 0.85 (d, *J* = 6.9 Hz, 3H); 0.79 (d, *J* = 6.9 Hz, 3H). $^{13}C\{^1H\}$ NMR (125 MHz with cryoprobe, $CDCl_3$, 298 K): *δ* (ppm) = 171.8; 171.7; 147.0; 145.4; 129.8; 123.9; 64.7; 57.1; 34.9; 31.1; 30.6; 28.4; 19.4; 19.0; 17.7. FTIR ν_{max} (cm^{-1}): 3318 (w); 2972 (w); 1727 (m); 1656 (m); 1518 (s); 1347 (s); 1246 (m). HRMS (ESI) *m/z*: $[M+Na]^+$ Calcd for $C_{23}H_{35}O_7N_3Na$ 488.2367; Found 488.2377.

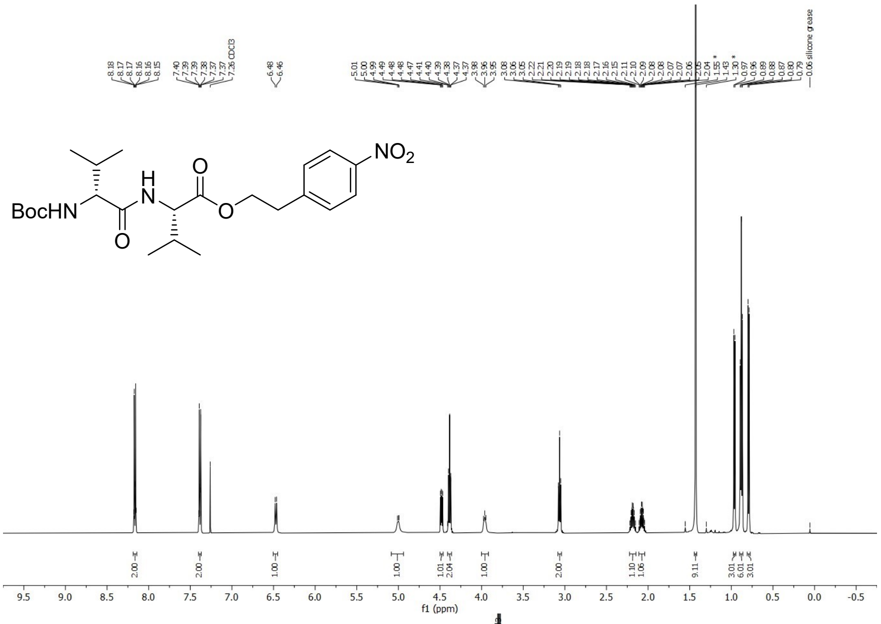

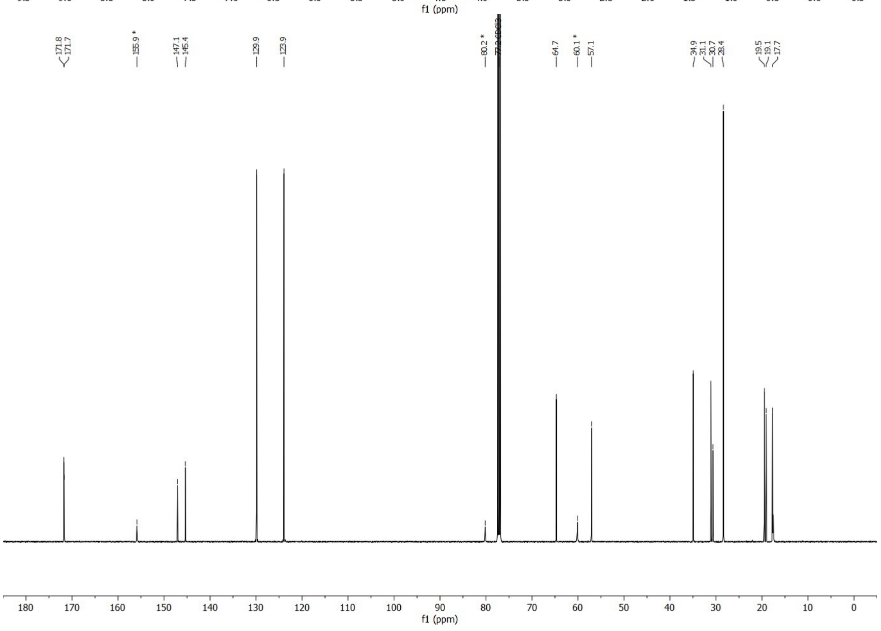

15$_{DD}$: Yield = 83%. Rf = 0.30 (7:3 iHex/EtOAc). ^{1}H NMR (500 MHz with cryoprobe, $CDCl_3$, 298 K): δ (ppm) = 8.17 – 8.14 (m, 2H); 7.39 – 7.36 (m, 2H); 6.42 (d, J = 8.6 Hz, 1H); 5.05 (d, J = 8.8 Hz, 1H); 4.46 (dd, J = 8.6 Hz, J = 5.0 Hz, 1H); 4.38 (ddt, J = 11.0 Hz, J = 6.8 Hz, J = 4.3 Hz, 2H); 3.88 (dd, J = 8.8 Hz, J = 6.5 Hz, 1H); 3.09 – 3.02 (m, 2H); 2.14 – 2.04 (m, 2H); 1.42 (s, 9H); 0.91 (dd, J = 11.2 Hz, J = 6.8 Hz, 6H); 0.85 (d, J = 6.9 Hz, 3H); 0.79 (d, J = 6.9 Hz, 3H). $^{13}C\{^{1}H\}$ NMR (125 MHz with cryoprobe, $CDCl_3$, 298 K): δ (ppm) = 171.8; 171.7; 147.0; 145.4; 129.8; 123.9; 64.7; 57.1; 34.9; 31.1; 30.6; 28.4; 19.4; 19.0; 17.7. FTIR ν_{max} (cm^{-1}): 3305 (w); 2966 (w); 1743 (m); 1651 (m); 1518 (s); 1345 (s); 1247 (m); 1161 (s). HRMS (ESI) m/z: $[M+Na]^{+}$ Calcd for $C_{23}H_{35}O_7N_3Na$ 488.2367; Found 488.2377.

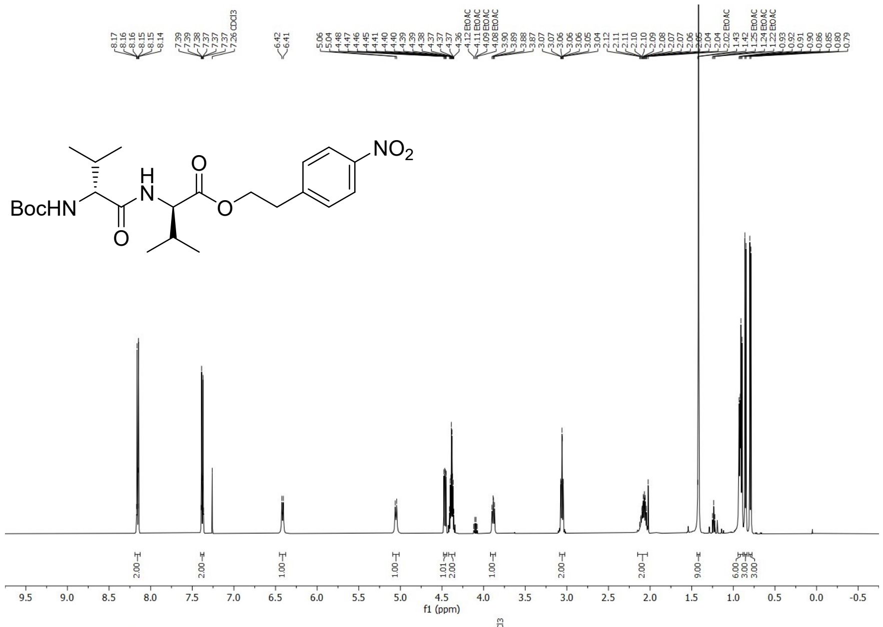

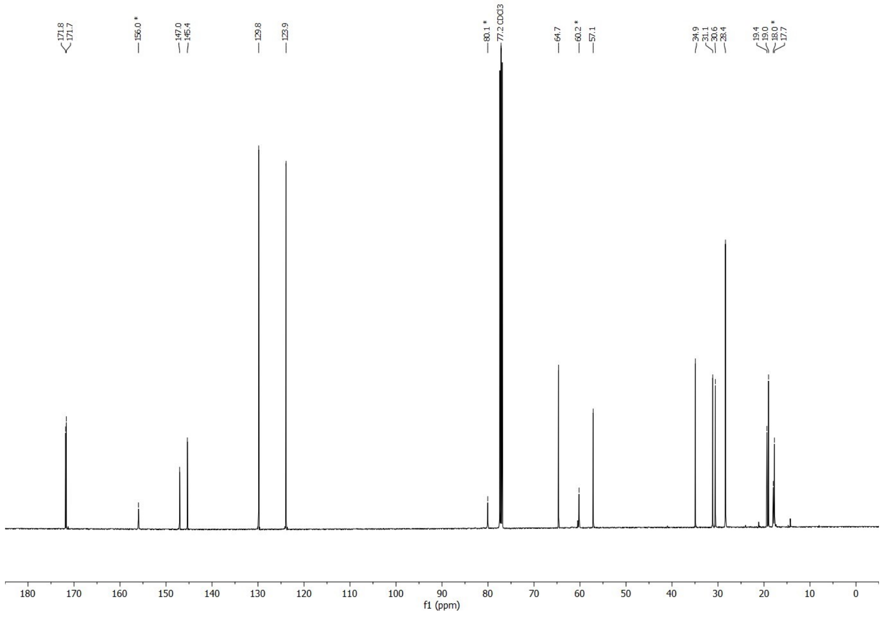

General procedure for the synthesis of compound 16: Compound **10** (1 equiv.) was dissolved in 4 M HCl/1,4-dioxane at 0°C. The reaction was stirred at 0°C for 5 min and at r.t. for 1 h. After that, the crude was concentrated under reduced pressure. The crude was triturated with Et_2O and filtered. The white precipitate was washed with additional Et_2O. Finally, the product was dried under high vacuum.

16_{LD}: Yield = 99%. ^1H NMR (500 MHz with cryoprobe, DMSO-d_6, 298 K): δ (ppm) = 8.80 (d, *J* = 8.3 Hz, 1H); 8.28 (s, 3H); 8.21 – 8.13 (m, 2H); 7.63 – 7.55 (m, 2H); 4.44 – 4.30 (m, 2H); 4.18 (dd, *J* = 8.3 Hz, *J* = 6.1 Hz, 1H); 3.78 (d, *J* = 5.2 Hz, 1H); 3.09 (t, *J* = 6.4 Hz, 2H); 2.14 (pd, *J* = 6.9 Hz, *J* = 5.2 Hz, 1H); 1.97 (h, *J* = 6.8 Hz, 1H); 0.95 (d, *J* = 6.9 Hz, 3H); 0.92 (d, *J* = 6.9 Hz, 3H); 0.78 (dd, *J* = 6.8 Hz, *J* = 1.5 Hz, 6H). $^{13}C\{^1H\}$ NMR (125 MHz with cryoprobe, DMSO-d_6, 298 K): δ (ppm) = 171.0; 168.4; 146.5; 146.3; 130.3; 123.4; 64.4; 57.6; 57.1; 34.0; 29.9; 29.9; 18.9; 18.5; 18.0; 17.4. FTIR ν_{max} (cm^{-1}): 3206 (w); 2968 (w); 1720 (w); 1680 (m); 1519 (s); 1464 (m); 1347 (s); 1223 (m); 856 (m). HRMS (ESI) *m/z*: $[M+H]^+$ Calcd for $C_{18}H_{28}O_5N_3$ 366.2023; Found 366.2029.

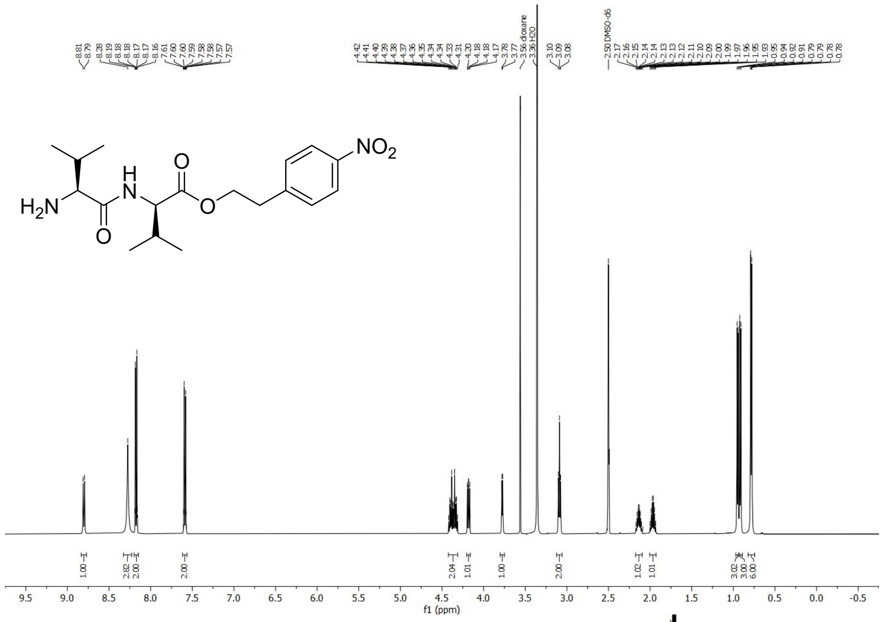

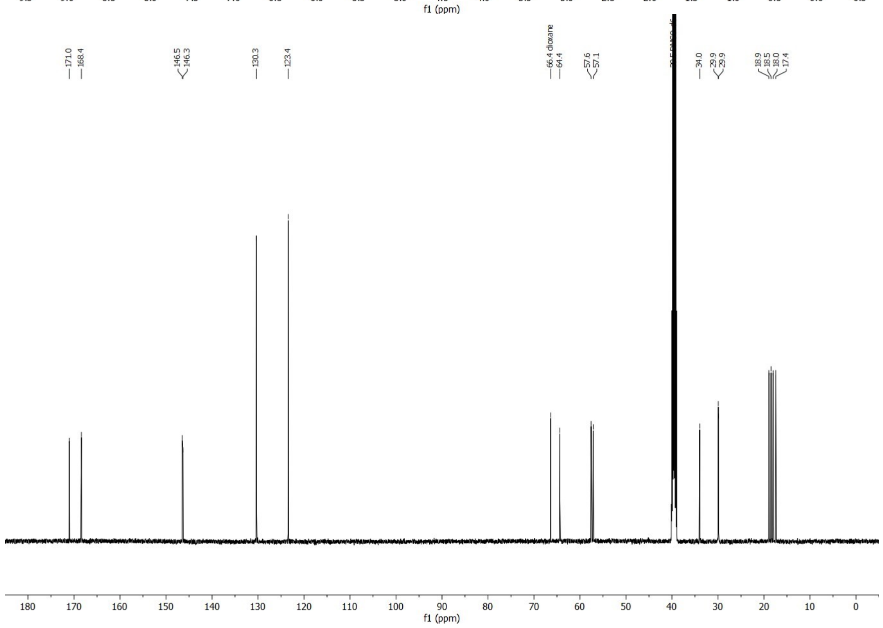

16_{DL}: Yield = 99%. ^{1}H NMR (500 MHz with cryoprobe, DMSO-d_6, 298 K): δ (ppm) = 8.79 (d, J = 8.3 Hz, 1H); 8.25 (s, 3H); 8.20 – 8.15 (m, 2H); 7.61 – 7.56 (m, 2H); 4.43 – 4.30 (m, 2H); 4.18 (dd, J = 8.2 Hz, J = 6.1 Hz, 1H); 3.77 (s, 1H); 3.09 (t, J = 6.4 Hz, 2H); 2.13 (pd, J = 6.9 Hz, J = 5.1 Hz, 1H); 1.96 (dq, J = 13.5 Hz, J = 6.8 Hz, 1H); 0.95 (d, J = 6.9 Hz, 3H); 0.91 (d, J = 6.9 Hz, 3H); 0.78 (d, J = 6.8 Hz, 6H). ^{13}C{^{1}H} NMR (125 MHz with cryoprobe, DMSO-d_6, 298 K): δ (ppm) = 171.0; 168.4; 146.5; 146.3; 130.4; 123.5; 64.4; 57.6; 57.2; 34.0; 30.0; 29.9; 18.9; 18.5; 18.0; 17.4. FTIR ν_{max} (cm^{-1}): 3206 (w); 2966 (w); 1733 (m); 1677 (m); 1518 (s); 1464 (m); 1344 (s); 1275 (m); 856 (m). HRMS (ESI) m/z: [M+H]$^+$ Calcd for $C_{18}H_{28}O_5N_3$ 366.2023; Found 366.2029.

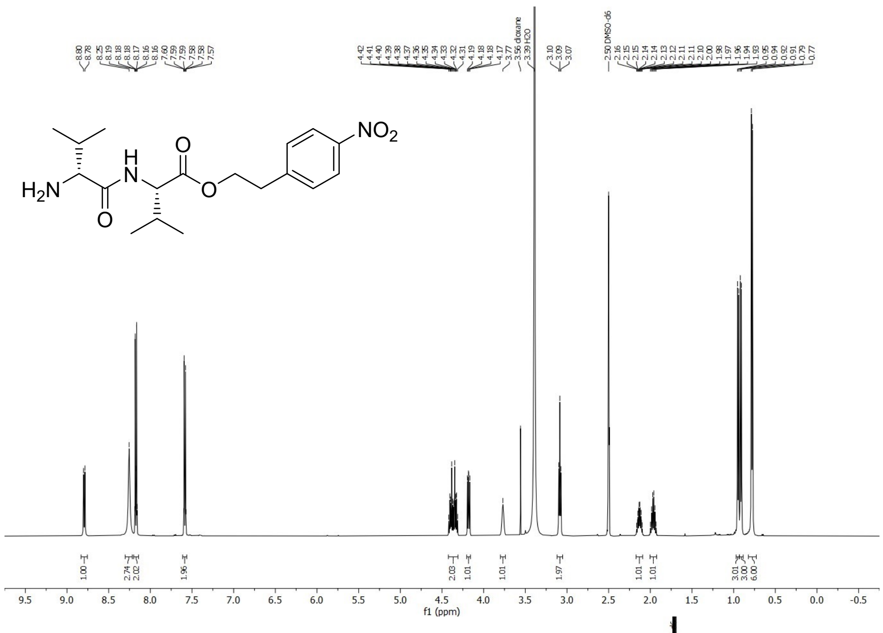

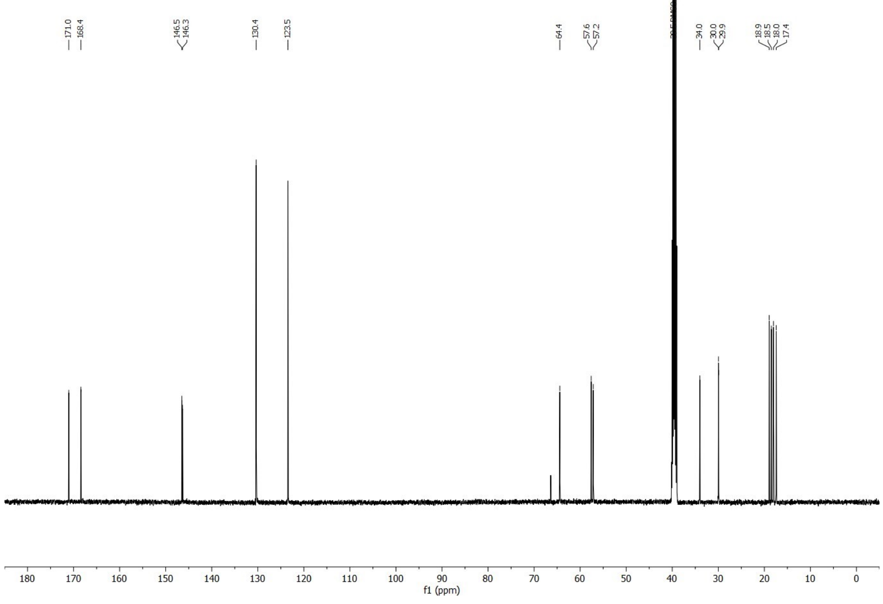

16_{DD}: Yield = 99%. ^{1}H NMR (500 MHz with cryoprobe, DMSO-d$_6$, 298 K): δ (ppm) = 8.66 (d, J = 7.4 Hz, 1H); 8.26 (s, 3H); 8.18 – 8.14 (m, 2H); 7.59 – 7.55 (m, 2H); 4.41 – 4.31 (m, 2H); 4.13 (dd, J = 7.4 Hz, J = 5.7 Hz, 1H); 3.78 (d, J = 5.6 Hz, 1H); 3.06 (t, J = 6.4 Hz, 2H); 2.09 – 1.95 (m, 2H); 0.91 (d, J = 2.0 Hz, 3H); 0.90 (d, J = 2.0 Hz, 3H); 0.82 (t, J = 6.5 Hz, 6H). ^{13}C{^{1}H} NMR (125 MHz with cryoprobe, DMSO-d$_6$, 298 K): δ (ppm) = 170.8; 168.4; 146.5; 146.3; 130.3; 123.4; 64.2; 57.8; 56.7; 34.0; 29.9; 29.6; 18.8; 18.2; 18.0; 17.7. FTIR ν_{max} (cm^{-1}): 3185 (w); 2966 (w); 1746 (m); 1660 (m); 1520 (s); 1447 (m); 1345 (s); 1187 (m); 1142 (m); 852 (m). HRMS (ESI) m/z: [M+H]$^+$ Calcd for $C_{18}H_{28}O_5N_3$ 366.2023; Found 366.2033.

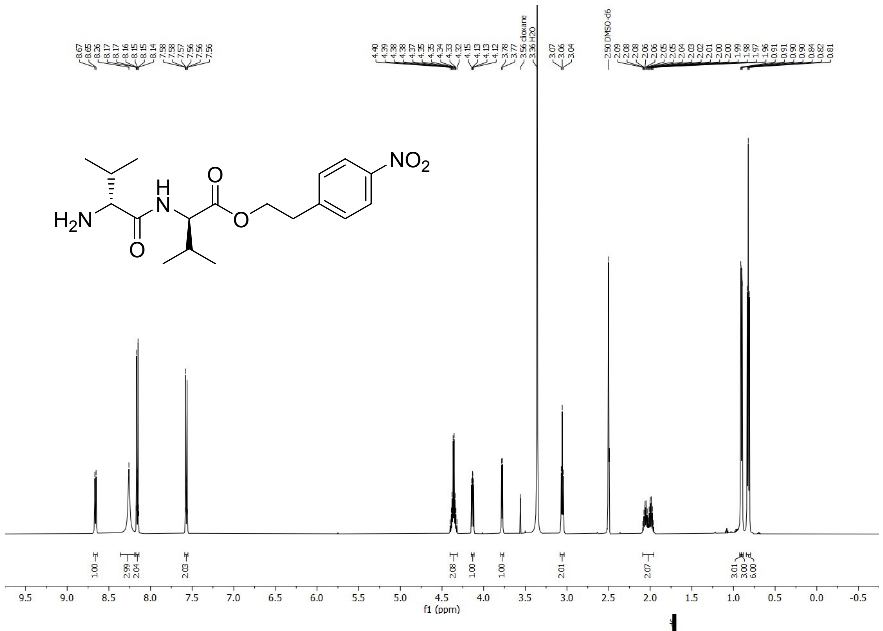

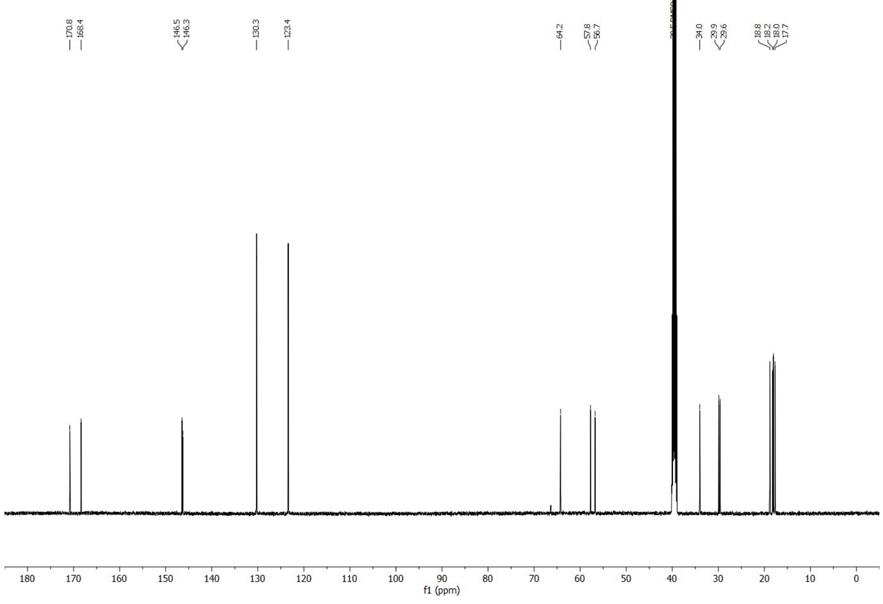

2.4 5-Methylaminomethyl uridine phosphoramidite

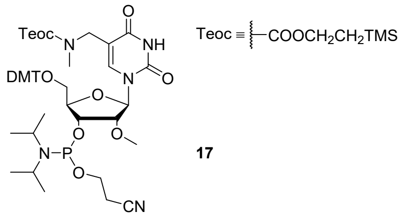

17

Figure S1. 5-methylaminomethyl uridine phosphoramidite **17**.

Compound **17** was synthesized following a procedure previously reported in the literature.[2]

2.5 5-Aminomethyl thymidine phosphoramidite

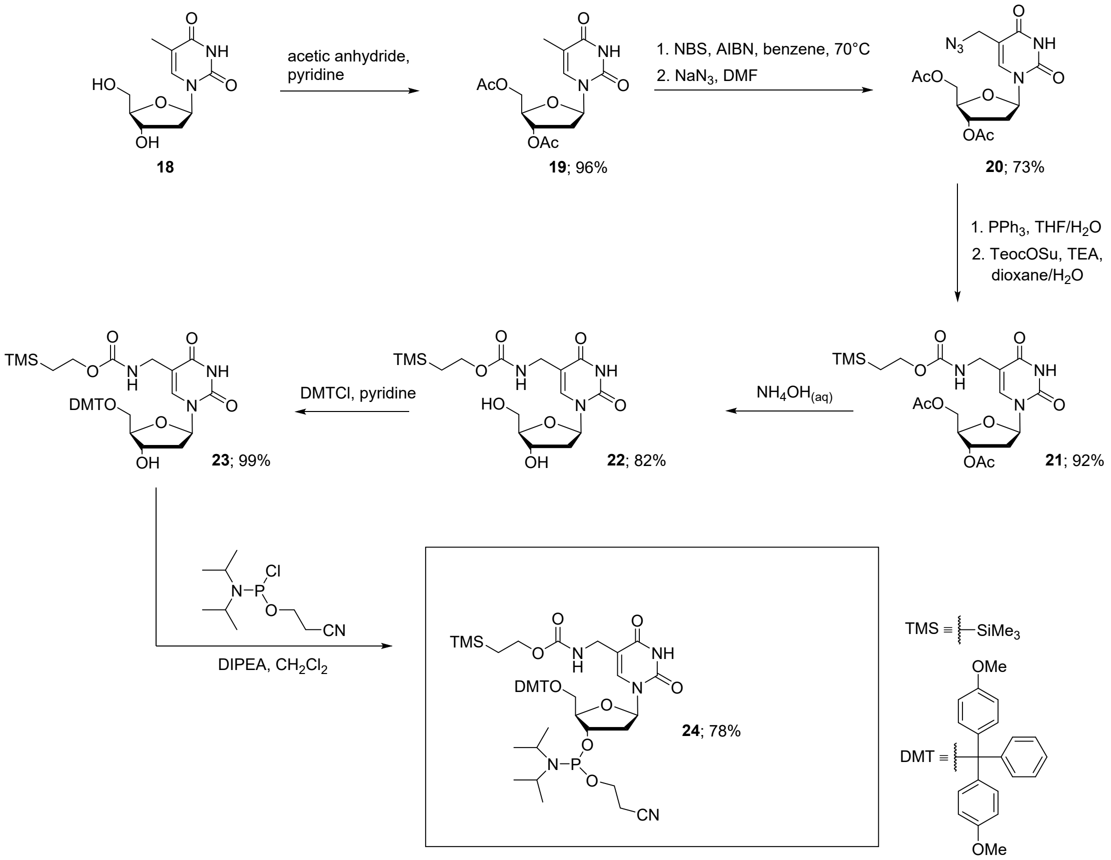

Scheme S4. Synthesis of 5-aminomethyl thymidine phosphoramidite.

General procedure for the synthesis of compound 19: Thymidine **18** (1 equiv.) was dissolved in dry pyridine, then acetic anhydride (3 equiv.) was added slowly. The mixture was stirred at r.t. for 2 h, then quenched with CH_2Cl_2, diluted with EtOAc and washed with water. The aqueous phase was extracted twice with EtOAc, then the combined organic layers were dried (Na_2SO_4), filtered and concentrated. The crude was purified by silica gel column chromatography.

19: Yield = 96%. Rf = 0.70 (10:0.5 CH_2Cl_2/MeOH). ^1H NMR (500 MHz with cryoprobe, $CDCl_3$, 298 K): δ (ppm) = 9.83 (d, J = 30.5 Hz, 1H); 7.25 (d, J = 1.6 Hz, 1H); 6.30 (ddd, J = 8.1 Hz, J = 5.6 Hz, J = 2.1 Hz, 1H); 5.18 (dq, J = 6.8 Hz, J = 2.2 Hz, 1H); 4.36 – 4.27 (m, 2H); 4.21 (dt, J = 4.0 Hz, J = 2.8 Hz, 1H); 2.46 – 2.40 (m, 1H); 2.18 – 2.10 (m, 1H); 2.09 (d, J = 2.8 Hz, 3H); 2.07 (d, J = 2.9 Hz, 3H); 1.92 – 1.87 (m, 3H). $^{13}C\{^1H\}$ NMR (125 MHz with cryoprobe, $CDCl_3$, 298 K): δ (ppm) = 170.5; 170.3; 163.9; 150.6; 134.6; 111.6; 84.8; 82.1; 74.2; 63.9; 37.5; 20.9; 20.9; 12.7. FTIR ν_{max} (cm^{-1}): 1747 (m); 1731 (m); 1700 (m); 1662 (s); 1474 (w); 1376 (m); 1243 (s); 1120 (s); 1093 (m); 1060 (m); 1027 (s); 951 (w); 883 (w); 865 (m); 763 (w); 629 (w). HRMS (ESI) m/z: $[M+H]^+$ Calcd for $C_{14}H_{19}N_2O_7$ 327.1187; Found 327.1191.

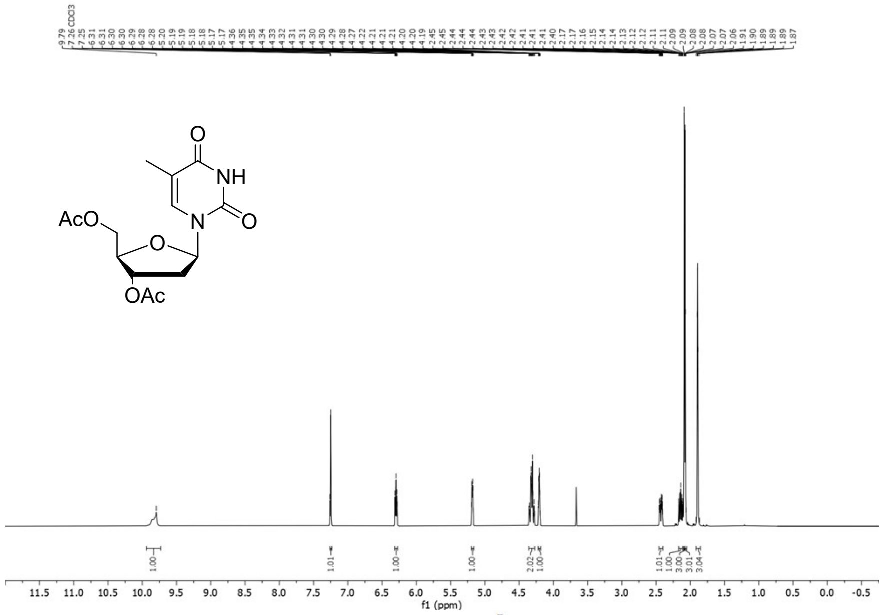

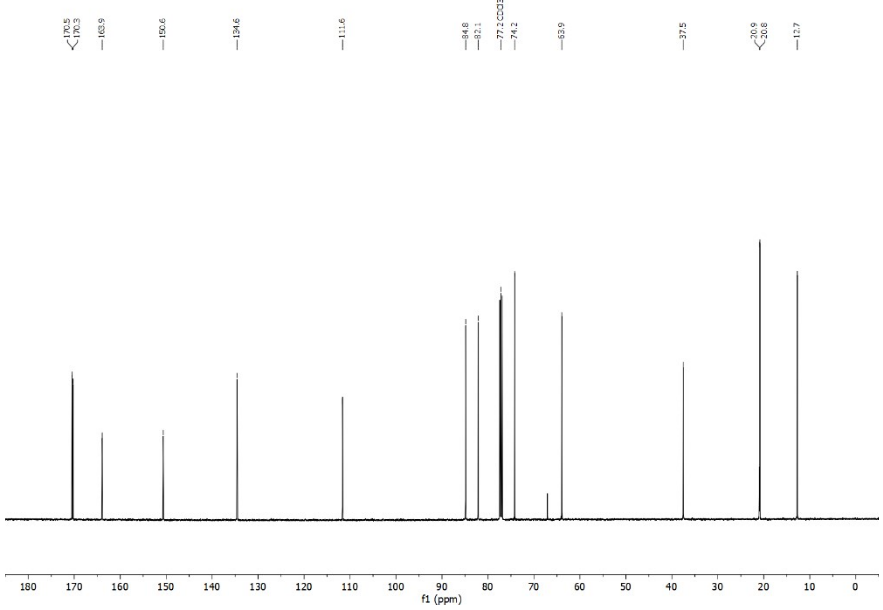

General procedure for the synthesis of compound 20: Compound **19** (1 equiv.) was suspended in dry benzene and degassed. *N*-Bromosuccinimide (1.2 equiv.) was added, the mixture was degassed again and heated to 70°C. Then, AIBN (0.5 equiv.) was added. The reaction mixture was stirred for 2 h, then another portion of AIBN (0.25 equiv.) was added. The reaction mixture was concentrated, the crude was dissolved in dry DMF under N_2 and NaN_3 (1 equiv.) was added. The reaction was stirred at r.t. for 20 h, then the reaction mixture was diluted with water. The aqueous phase was extracted with EtOAc, then the combined organic layers were dried (Na_2SO_4), filtered and concentrated. The crude was purified by silica gel column chromatography.

20: Yield = 73%. Rf = 0.35 (1:3 iHex/EtOAc). ^{1}H NMR (500 MHz with cryoprobe, DMSO-d_6, 298 K): δ (ppm) = 11.63 (s, 1H); 7.82 (s, 1H); 6.16 (dd, *J* = 8.0 Hz, *J* = 6.2 Hz, 1H); 5.20 (dt, *J* = 6.4 Hz, *J* = 2.9 Hz, 1H); 4.24 (dd, *J* = 4.8 Hz, *J* = 3.4 Hz, 2H); 4.18 (td, *J* = 4.8 Hz, *J* = 2.9 Hz, 1H); 4.10 (s, 2H); 2.43 (ddd, *J* = 14.6 Hz, *J* = 8.1 Hz, *J* = 6.8 Hz, 1H); 2.34 (ddd, *J* = 14.4 Hz, *J* = 6.3 Hz, *J* = 2.8 Hz, 1H); 2.05 (d, *J* = 9.1 Hz, 6H). ^{13}C{^{1}H} NMR (125 MHz with cryoprobe, DMSO-d_6, 298 K): δ (ppm) = 170.4; 170.3; 163.0; 150.4; 139.8; 109.1; 84.8; 81.5; 74.1; 63.9; 46.9; 36.0; 20.9; 20.7. FTIR ν_{max} (cm^{-1}): 2106 (w); 1681 (s); 1602 (w); 1464 (m); 1366 (m); 1224 (s); 1100 (m); 685 (w); 603 (w). HRMS (ESI) *m/z*: $[M-H]^-$ Calcd for $C_{14}H_{16}N_5O_7$ 366.1055; Found 366.1056.

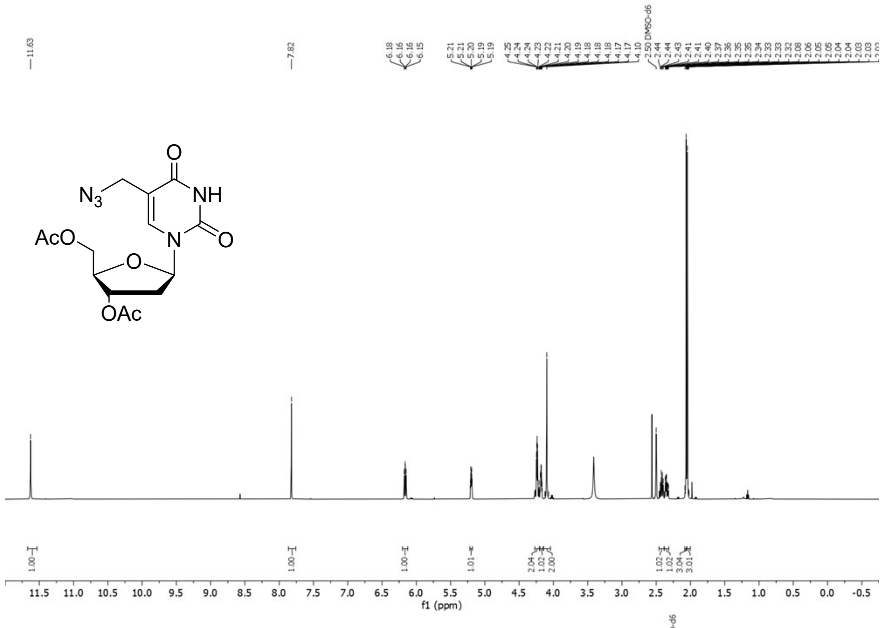

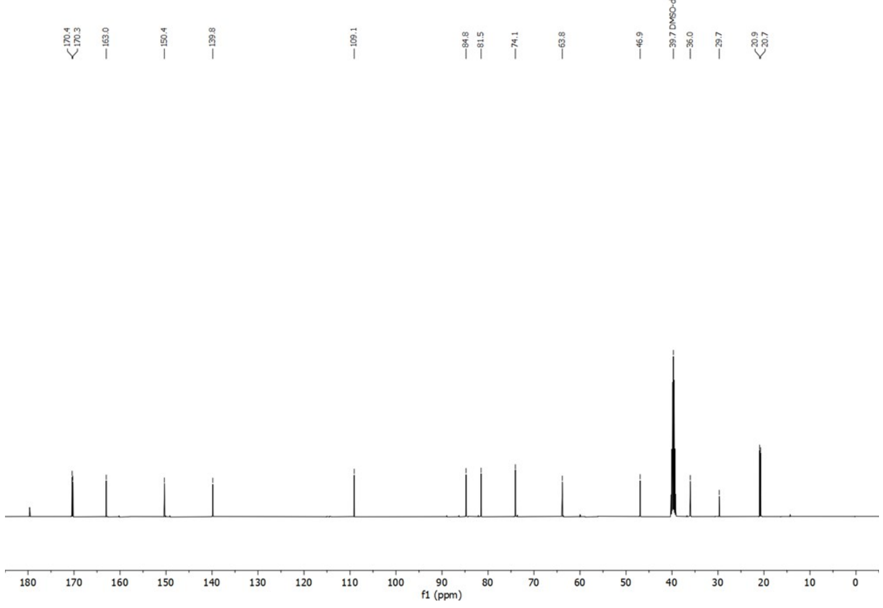

General procedure for the synthesis of compound 21: Step 1. Compound **20** (1 equiv.) was dissolved in THF/H_2O mixture (10:1) and PPh_3 (1.5 equiv.). was added. The reaction was stirred for 3 h, then the volatiles were evaporated and the crude was used for the next step without further purification. Step 2. The crude (1 equiv.) was dissolved in dioxane/water mixture (1:1) and TeocOSu (1.1 equiv.) was added, followed by TEA (1.5 equiv.). The reaction mixture was stirred at r.t. for 20 h. After that, the crude was diluted with EtOAc, washed with water, dried (Na_2SO_4), filtered and concentrated. The crude was purified by silica gel column chromatography.

21: Yield = 92%. Rf = 0.25 (3:7 iHex/EtOAc). ^1H NMR (500 MHz with cryoprobe, DMSO-d_6, 298 K): δ (ppm) = 11.47 (s, 1H); 7.51 (s, 1H); 7.28 – 7.09 (m, 1H); 6.17 (t, J = 7.1 Hz, 1H); 5.19 (dt, J = 5.9 Hz, J = 2.9 Hz, 1H); 4.25 – 4.15 (m, 3H); 4.02 (dt, J = 9.0 Hz, J = 3.8 Hz, 2H); 3.82 (d, J = 5.6 Hz, 2H); 2.37 – 2.28 (m, 2H); 2.07 (d, J = 5.2 Hz, 6H); 0.94 – 0.85 (m, 2H); 0.00 (s, 9H). $^{13}C\{^1H\}$ NMR (125 MHz with cryoprobe, DMSO-d_6, 298 K): δ (ppm) = 170.4; 170.2; 162.9; 156.6; 150.4; 137.3; 111.7; 84.6; 81.5; 74.2; 63.9; 61.9; 36.9; 36.2; 20.9; 20.8; 17.6; -1.3. FTIR ν_{max} (cm^{-1}): 1672 (s); 1467 (w); 1376 (w); 1221 (s); 1135 (w); 1100 (m); 1059 (m); 1026 (m); 951 (w); 860 (m); 834 (m); 763 (w); 694 (w); 603 (w). HRMS (ESI) m/z: [M-H]$^-$ Calcd for $C_{20}H_{30}N_3O_9Si$ 484.1757; Found 484.1760.

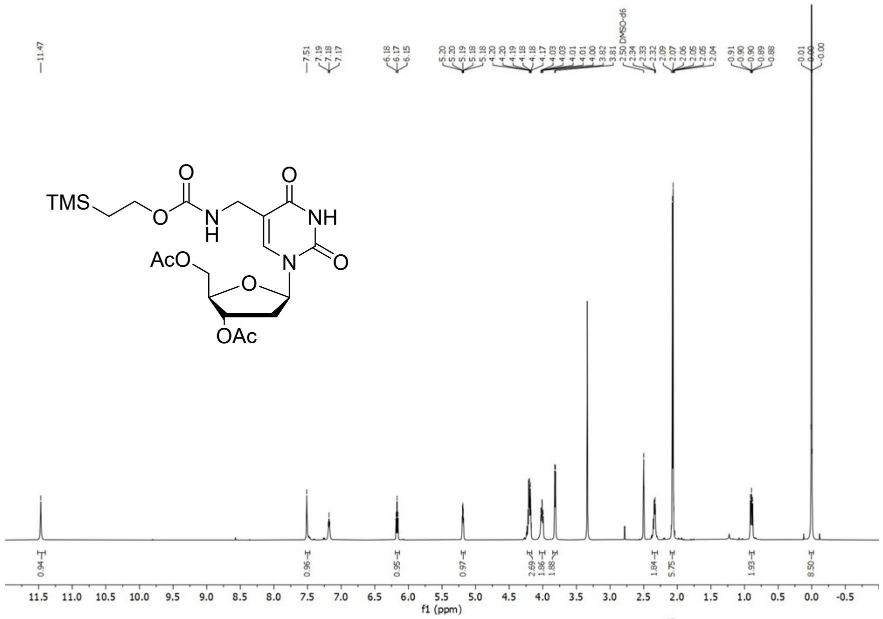

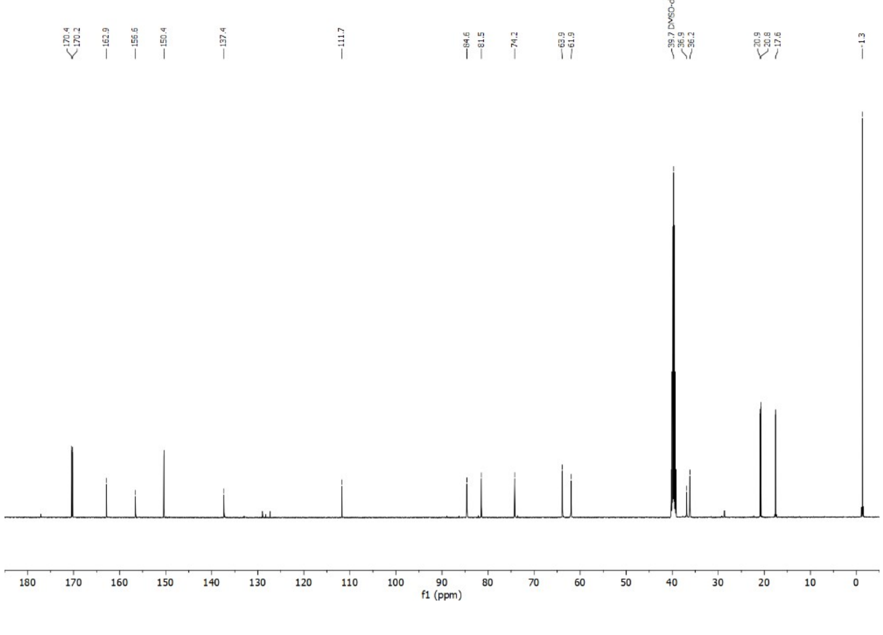

General procedure for the synthesis of compound 22: Compound **21** was dissolved in aq. NH_4OH and stirred at r.t. for 3 h. The reaction mixture was extracted with EtOAc, the organic layers were washed with water, then with aq. NH_4Cl solution, then dried (Na_2SO_4), filtered and concentrated. The crude was purified by silica gel column chromatography to afford the product as a white solid.

22: Yield = 82%. Rf = 0.38 (10:1 CH_2Cl_2/MeOH). ^{1}H NMR (500 MHz with cryoprobe, DMSO-*d*$_6$, 298 K): δ (ppm) = 11.36 (s, 1H); 7.65 (s, 1H); 7.13 (t, *J* = 5.6 Hz, 1H); 6.16 (dd, *J* = 7.5 Hz, *J* = 6.1 Hz, 1H); 5.25 (d, *J* = 4.2 Hz, 1H); 4.90 (t, *J* = 5.3 Hz, 1H); 4.22 (dq, *J* = 6.5 Hz, *J* = 3.1 Hz, 1H); 4.09 – 3.95 (m, 2H); 3.78 (dd, *J* = 8.2 Hz, *J* = 4.4 Hz, 3H); 3.57 – 3.49 (m, 2H); 2.14 – 1.96 (m, 2H); 0.91 (dd, *J* = 9.4 Hz, *J* = 7.4 Hz, 2H); 0.01 (s, 9H). $^{13}C\{^{1}H\}$ NMR (125 MHz with cryoprobe, DMSO-*d*$_6$, 298 K): δ (ppm) = 162.9; 156.5; 150.5; 137.7; 111.2; 87.6; 84.3; 70.8; 61.9; 61.8; 37.0; 17.6; -1.2. FTIR ν_{max} (cm^{-1}): 2953 (w); 1673 (s); 1468 (m); 1247 (s); 1092 (m); 1049 (m); 941 (m); 833 (s); 761 (m); 694 (m). HRMS (ESI) *m/z*: $[M-H]^-$ Calcd for $C_{16}H_{26}N_3O_7Si$ 400.1546; Found 400.1548.

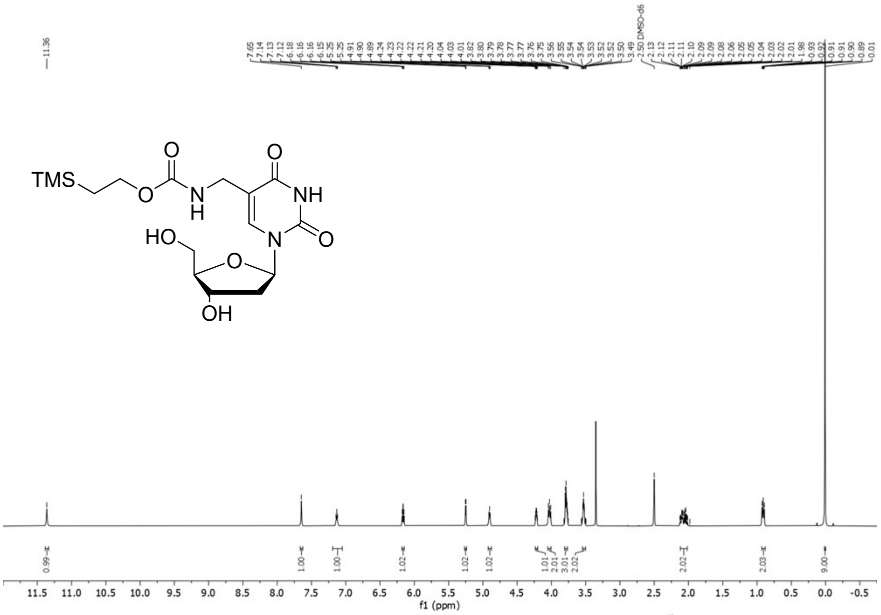

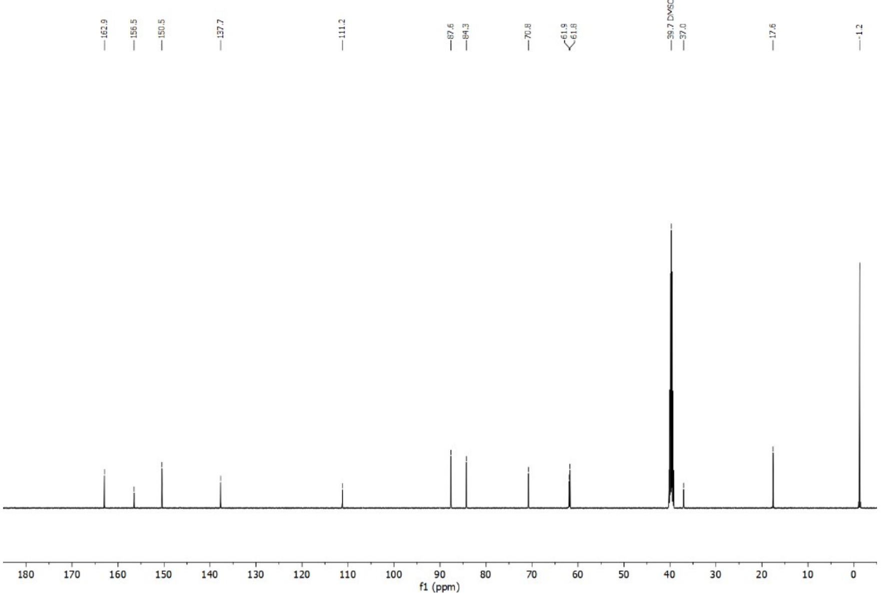

Compound **23** was synthesized following a procedure described above for compound **6** (Section 2.1).

23: Yield = 99%. Rf = 0.44 (1:2.5 iHex/EtOAc). ^{1}H NMR (500 MHz with cryoprobe, DMSO-d_6, 298 K): δ (ppm) = 11.44 (s, 1H); 8.58 – 8.56 (m, 2H); 7.77 (tt, J = 7.6 Hz, J = 1.8 Hz, 1H); 7.42 – 7.34 (m, 4H); 7.33 – 7.24 (m, 6H); 7.24 – 7.18 (m, 1H); 6.91 – 6.85 (m, 4H); 6.16 (t, J = 6.6 Hz, 1H); 5.33 (d, J = 4.7 Hz, 1H); 4.22 (p, J = 5.1 Hz, 1H); 3.99 – 3.93 (m, 2H); 3.87 (q, J = 4.3 Hz, 1H); 3.73 (s, 6H); 3.59 (dd, J = 5.2 Hz, J = 2.5 Hz, 2H); 3.20 (qd, J = 10.4 Hz, J = 4.5 Hz, 2H); 2.17 (t, J = 6.0 Hz, 2H); 0.86 (td, J = 8.0 Hz, J = 3.4 Hz, 2H); -0.01 (s, 9H). ^{13}C{^{1}H} NMR (125 MHz with cryoprobe, DMSO-d_6, 298 K): δ (ppm) = 162.9; 158.3; 156.3; 150.4; 149.8; 145.0; 138.3; 136.3; 135.8; 135.6; 129.9; 128.0; 127.9; 126.9; 124.1; 113.4; 110.9; 85.9; 85.6; 84.4; 70.5; 64.0; 61.7; 55.2; 17.6; -1.3. FTIR v_{max} (cm^{-1}): 2951 (w); 1649 (s); 1608 (w); 1507 (m); 1464 (m); 1247 (s); 1175 (m); 1090 (m); 1031 (s); 967 (w); 827 (s); 791 (m); 754 (m); 727 (w); 701 (s). HRMS (ESI) m/z: [M-H]$^{-}$ Calcd for $C_{37}H_{44}N_3O_9Si$ 702.2852; Found 702.2860.

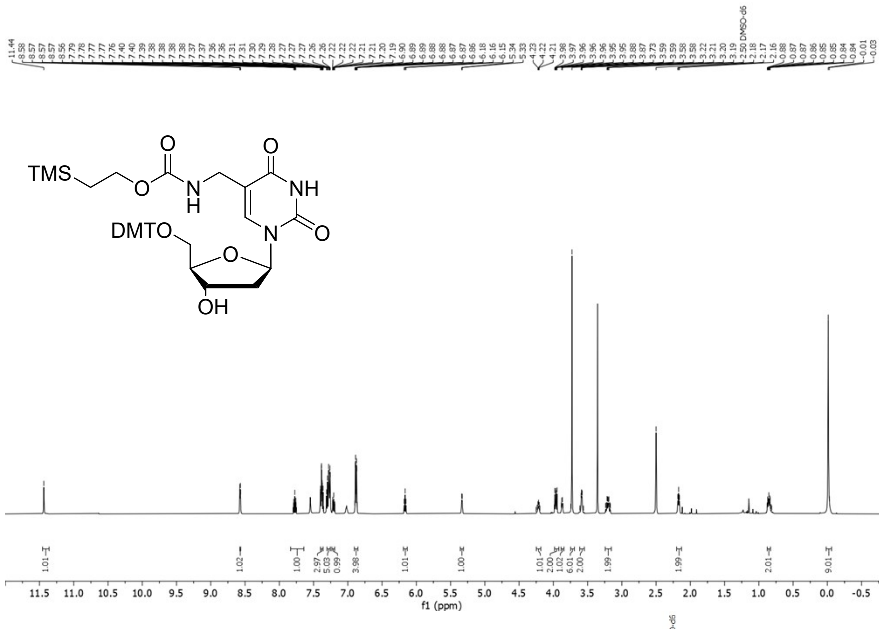

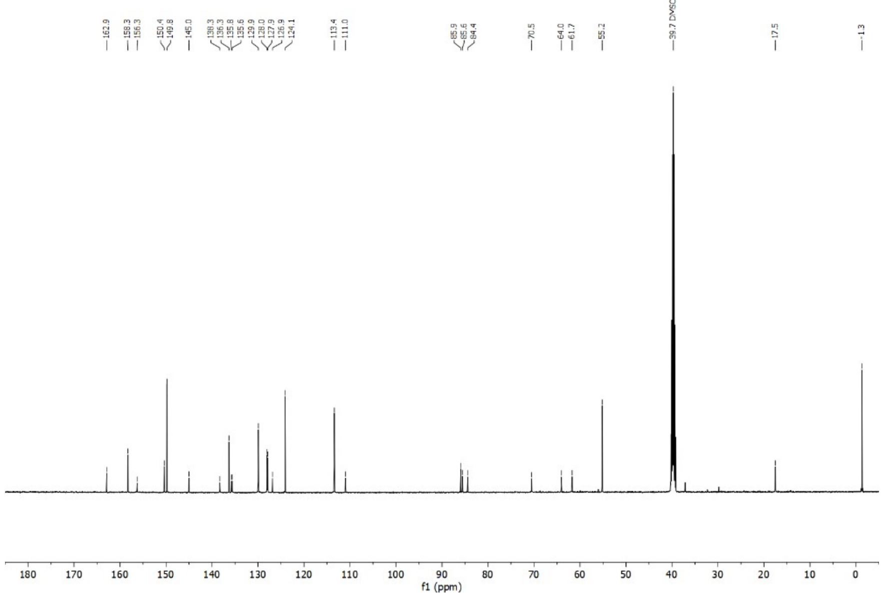

Compound **24** was synthesized following a procedure describe above for compound **7** (Section 2.1).

24: Yield = 78%. Rf = 0.53 (1:1 iHex/EtOAc). $^{31}P\{^{1}H\}$ NMR (202 MHz with cryoprobe, acetone-d_6, 298 K): δ (ppm) = 148.3; 148.2. HRMS (ESI) m/z: $[M+Cl]^-$ Calcd for $C_{46}H_{62}N_5O_{10}PSiCl$ 938.3698; Found 938.3699.

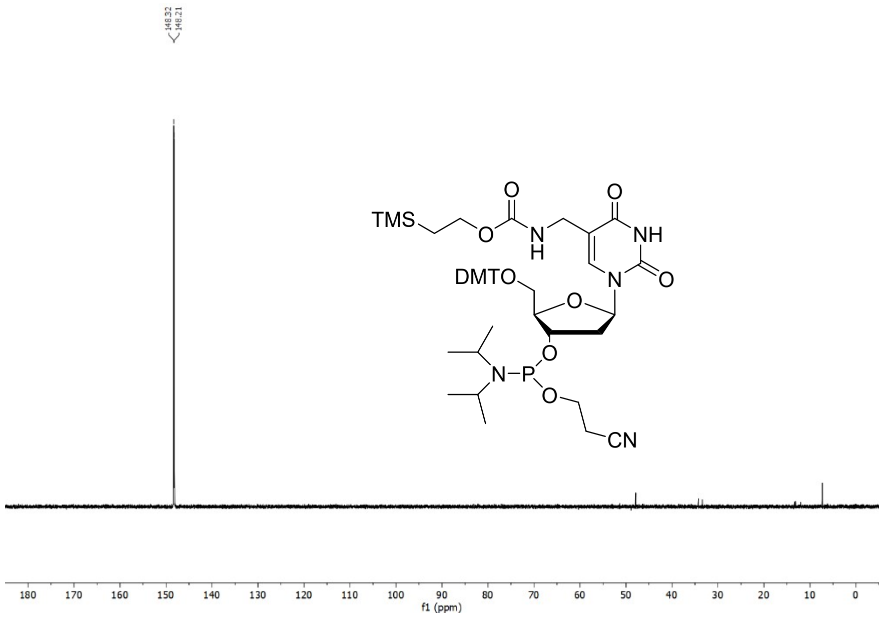

3. General information and instruments for oligonucleotides

Synthesis and purification of oligonucleotides

Phosphoramidites of 2'-O-Me ribonucleosides (2'-OMe-Bz-A-CE, 2'-OMe-Dmf-G-CE, 2'-OMe-Ac-C-CE and 2'-OMe-U-CE) and deoxyribonucleosides (Bz-dA-CE, Dmf-dG-CE, Ac-dC-CE, T-CE) were purchased from LinkTech and Sigma-Aldrich. Oligonucleotides (ONs) were synthesized on a 1 µmol scale using RNA SynBase™ CPG 1000/110 and High Load Glen UnySupport™ as solid supports using an RNA automated synthesizer (Applied Biosystems 394 DNA/RNA Synthesizer) with a standard phosphoramidite chemistry. ONs were synthesized in DMT-OFF mode using DCA as a deblocking agent in CH_2Cl_2, BTT or Activator 42® as activator in MeCN, Ac_2O as capping reagent in pyridine/THF and I_2 as oxidizer in pyridine/H_2O.

Deprotection of Onpe and teoc groups

For the deprotection of the *para*-nitrophenylethyl (Onpe) group in ONs containing amino acid-modified carbamoyl adenosine nucleosides, the solid support beads were suspended in a 9:1 THF/DBU solution mixture (1 mL) and incubated at r.t. for 2 h.[4] After that, the supernatant was removed, and the beads were washed with THF (3×1 mL).

For the deprotection of the 2-(trimethylsilyl)ethoxycarbonyl (teoc) group in ONs containing 5-methylaminomethyl uridine nucleosides, the solid support beads were suspended in a saturated solution of $ZnBr_2$ in 1:1 $MeNO_2$/IPA (1 mL) and incubated at r.t. overnight.[5] After that, the supernatant was removed and the beads were washed with 0.1 M EDTA in water (1 mL) and water (1 mL).

Coupling of amino acids to ONs anchored to the solid support beads

The solid support beads (1 µmol) in an Eppendorf tube were washed with dry DMF (0.3 mL). In a separate Eppendorf tube, Boc-protected L- or D-Valine, DMTMM•BF_4 (100 µmol) as activator and dry DIPEA (200 µmol) were dissolved in dry DMF (0.6 mL). Subsequently, the amino acid solution was added to the solid support beads and the reaction was incubated in an orbital shaker at r.t. for 1 h. The suspension was centrifuged and the supernatant was removed. The solid support beads were washed with dry DMF (2×0.3 mL) and dry MeCN (2×0.3 mL). Finally, the beads were dried using a SpeedVac concentrator.

For the deprotection of the *tert*-butyloxycarbonyl (Boc) group in ONs after the coupling of a Boc-protected amino acid or peptide, the solid support beads were suspended in a 1:1 TFA/CH_2Cl_2 solution mixture (0.5 mL) and incubated for 5 min at r.t.[6] After that, the supernatant was removed and the solid support beads were washed with CH_2Cl_2 (2×0.5 mL).

Cleavage from beads and precipitation of the synthesized ON

The solid support beads were suspended in a 1:1 aqueous solution mixture (0.6 mL) of 30% NH_4OH and 40% $MeNH_2$. The suspension was heated at 65°C (8 min for SynBase™ CPG 1000/110 and 60 min for High Load Glen UnySupport™). The ONs containing dipeptide-modified carbamoyl adenosine derivatives were cleaved from the solid support beads using a 30% NH_4OH aqueous solution (0.6 mL) at r.t. overnight. Subsequently, the supernatant was collected, and the beads were washed with water (2×0.3 mL). The combined aqueous solutions were concentrated under reduced pressure using a SpeedVac concentrator. After that, the crude was dissolved in DMSO (100 µL) and the ON was precipitated by adding 3 M NaOAc in water (25 µL) and *n*-butanol (1 mL). The mixture was kept at -80°C for 2 h and centrifuged at 4°C for 1 h. The supernatant was removed, and the white precipitate was lyophilized.

Purification of the synthesized ON by HPLC and desalting

The crude was purified by semi-preparative HPLC (1260 Infinity II Manual Preparative LC System from Agilent equipped with a G7114A detector) using a reverse-phase (RP) VP 250/10 Nucleodur 100-5 C18ec column from Macherey-Nagel (buffer A: 0.1 M AcOH/Et_3N pH 7 in H_2O and buffer B: 0.1 M AcOH/Et_3N pH 7 in 20:80 H_2O/MeCN; Gradient: 0-25% of B in 45 min; Flow rate = 5 mL·min^{-1}). The purified ON was analyzed by RP-HPLC (1260 Infinity II LC System from Agilent equipped with a G7165A detector) using an EC 250/4 Nucleodur 100-3 C18ec from Macherey-Nagel (Gradient: 0-30% of B in 45 min; Flow rate = 1 mL·min^{-1}). Finally, the purified ON was desalted using a C18 RP-cartridge from Waters.

Determination of the concentration and the mass of the synthesized ON

The absorbance of the synthesized ON in H_2O solution was measured using an IMPLEN NanoPhotometer® N60/N50 at 260 nm. The extinction coefficient of the ON was calculated using the OligoAnalyzer Version 3.0 from Integrated DNA Technologies. For ONs incorporating non-canonical bases, the extinction coefficients were assumed to be identical to those containing only canonical counterparts.

The synthesized ON (2-3 µL) was desalted on a 0.025 µm VSWP filter (Millipore), co-crystallized in a 3-hydroxypicolinic acid matrix (HPA, 1 µL) and analyzed by matrix-assisted laser desorption/ionization – time-of-flight (MALDI-ToF) mass spectrometry (negative mode).

4. Synthesized oligonucleotides using a DNA/RNA automated synthesizer

4.1 Donor strands containing amino acid-modified methyl N^6-carbamoyl adenosine at the 5'-end

Table S1. HPLC retention times (0-40% of B in 45 min) and MALDI-ToF mass spectrometric analysis (negative mode) of donor strands **ON1$_{R1}$** containing a Val- or Val-Val-modified N^6-carbamoyl adenosine at the 5'-end. Subscript $_L$ stands for the L-Val enantiomer and subscript $_D$ stands for the D-Val enantiomer.

Sequence	Donor strand	t_R (min)	*m/z* calcd. for [*M*-H]$^-$	found
5'-**R^1**(AUCGCU)$_m$-3'	**ON1$_L$**; **R^1** = $m^6v_L{}^6A_m$	27.8	2417.5	2417.2
	**ON1$_D$**; **R^1** = $m^6v_D{}^6A_m$	28.9	2417.5	2417.0
	**ON1$_{LL}$**; **R^1** = $m^6v_Lv_L{}^6A_m$	30.1	2516.5	2516.5
	**ON1$_{LD}$**; **R^1** = $m^6v_Lv_D{}^6A_m$	31.0	2516.5	2516.3
	**ON1$_{DL}$**; **R^1** = $m^6v_Dv_L{}^6A_m$	32.8	2516.5	2516.2
	**ON1$_{DD}$**; **R^1** = $m^6v_Dv_D{}^6A_m$	33.9	2516.5	2516.4

4.2 Acceptor strands containing 5-methylaminomethyl uridine at the 3'-end

Table S2. HPLC retention times (0-40% of B in 45 min) and MALDI-ToF mass spectrometric analysis (negative mode) of acceptor strands **ON2$_{R2}$** containing a methylaminomethyl uridine at the 3'-end.

Sequence	Acceptor strand	t_R (min)	*m/z* calcd. for [*M*-H]$^-$	found
5'-(GUACAGCGAU)$_m$**R^2**-3'	**ON2$_L$**; **R^2** = v_L-mnm^5U_m	24.8	3785.4	3786.0
	**ON2$_D$**; **R^2** = v_D-mnm^5U_m	24.7	3785.4	3788.7

4.3 Donor strands containing amino acid-modified methyl N^6-carbamoyl deoxy adenosine at the 5'-end

Table S3. HPLC retention times (0-40% of B in 45 min) and MALDI-ToF mass spectrometric analysis (negative mode) of donor strands **dON1$_{R1}$** containing an L- or D-Val-modified N^6-carbamoyl deoxy adenosine at the 5'-end.

Sequence	Donor strand	t_R (min)	*m/z* calcd. for [*M*-H]$^-$	found
5'- **R^1**d(ATCGCT)-3'	**dON1$_L$**; **R^1** = $m^6v_L{}^6$dA	24.5	2236.6	2236.3
	**dON1$_D$**; **R^1** = $m^6v_D{}^6$dA	23.2	2236.6	2236.2

4.4 Acceptor strands containing 5-aminomethyl thymidine at the 3'-end

Table S4: HPLC retention times (0-40% of B in 45 min) and MALDI-ToF mass spectrometric analysis (negative mode) of acceptor strands **dON2$_{R2}$** containing an aminomethyl thymidine at the 3'-end.

Sequence	Acceptor strand	t_R (min)	*m/z* calcd. for [*M*-H]$^-$	found
5'-d(GTACAGCGAT)**R^2**-3'	**dON2$_L$**; **R^2** = v_L-nm^{5}dT	19.3	3470.4	3469.1
	**dON2$_D$**; **R^2** = v_D-nm^{5}dT	19.2	3470.4	3469.8

5. Peptide coupling reactions between donor and acceptor oligonucleotides

Stock solutions of MES buffer pH 6 (400 mM), NaCl (1 M) and activator (500 mM) were prepared in water. Subsequently, equimolar amounts of (**d**)**ON1**$_{\mathbf{R1}}$ and (**d**)**ON2**$_{\mathbf{R2}}$ (3-5 nmol) were annealed at 95°C for 4 min in water containing NaCl (half of the volume required for the reaction). Finally, buffer, NaCl, activator solutions and water were added to the ON solution and the reaction was incubated in a ThermoMixer at 25°C for 2 h. The concentration of the components in the reaction mixture was: 50 µM of (**d**)**ON1**$_{\mathbf{R1}}$, 50 µM of (**d**)**ON2**$_{\mathbf{R2}}$, 100 mM of buffer, 100 mM of NaCl and 50 mM of EDC/Sulfo-NHS.

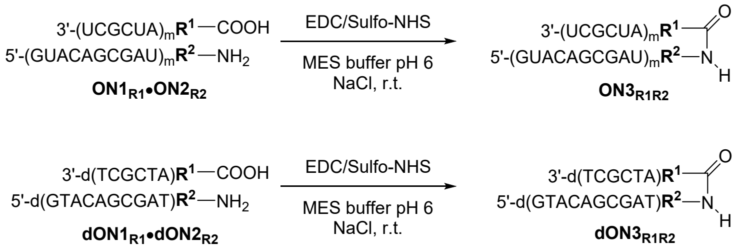

Scheme S5. Peptide coupling reactions between oligonucleotides (**d**)**ON1**$_{\mathbf{R1}}$ and (**d**)**ON2**$_{\mathbf{R2}}$ to give hairpin (**d**)**ON3**$_{\mathbf{R1R2}}$.

The peptide coupling reactions were only performed to isolate and characterize the products by HPLC (t_R) and mass spectrometry (*m/z*). The isolated products were used as references for the analyses of the pair-wise competitive reactions. In this respect, the reaction yields are not given.

Table S5. HPLC retention times (0-40% of B in 45 min) and MALDI-ToF mass spectrometric analysis (negative mode) of isolated hairpin products (**d**)**ON3**$_{\mathbf{R1R2}}$.

Donor strand	Acceptor strand	Hairpin strand	t_R (min)	*m/z* calcd. for [*M*-H]$^-$	found
**ON1**$_{\mathbf{L}}$; **R^1** = m^{6}v$_L$^{6}A$_m$	**ON2**$_{\mathbf{L}}$; **R^2** = v$_L$-mnm^{5}U$_m$	**ON3**$_{\mathbf{LL}}$	32.2	6186.9	6186.1
**ON1**$_{\mathbf{D}}$; **R^1** = m^{6}v$_D$^{6}A$_m$	**ON2**$_{\mathbf{L}}$; **R^2** = v$_L$-mnm^{5}U$_m$	**ON3**$_{\mathbf{DL}}$	33.6	6186.9	6187.3
**ON1**$_{\mathbf{LL}}$; **R^1** = m^{6}v$_L$v$_L$^{6}A$_m$	**ON2**$_{\mathbf{L}}$; **R^2** = v$_L$-mnm^{5}U$_m$	**ON3**$_{\mathbf{LLL}}$	34.0	6285.9	6286.7
**ON1**$_{\mathbf{LL}}$; **R^1** = m^{6}v$_L$v$_L$^{6}A$_m$	**ON2**$_{\mathbf{D}}$; **R^2** = v$_D$-mnm^{5}U$_m$	**ON3**$_{\mathbf{LLD}}$	36.1	6285.9	6285.6
**ON1**$_{\mathbf{LD}}$; **R^1** = m^{6}v$_L$v$_D$^{6}A$_m$	**ON2**$_{\mathbf{L}}$; **R^2** = v$_L$-mnm^{5}U$_m$	**ON3**$_{\mathbf{LDL}}$	35.5	6285.9	6285.9
**ON1**$_{\mathbf{LD}}$; **R^1** = m^{6}v$_L$v$_D$^{6}A$_m$	**ON2**$_{\mathbf{D}}$; **R^2** = v$_D$-mnm^{5}U$_m$	**ON3**$_{\mathbf{LDD}}$	33.5	6285.9	6286.2
**ON1**$_{\mathbf{DL}}$; **R^1** = m^{6}v$_D$v$_L$^{6}A$_m$	**ON2**$_{\mathbf{L}}$; **R^2** = v$_L$-mnm^{5}U$_m$	**ON3**$_{\mathbf{DLL}}$	34.6	6285.9	6286.9
**ON1**$_{\mathbf{DL}}$; **R^1** = m^{6}v$_D$v$_L$^{6}A$_m$	**ON2**$_{\mathbf{D}}$; **R^2** = v$_D$-mnm^{5}U$_m$	**ON3**$_{\mathbf{DLD}}$	35.5	6285.9	6285.7
**ON1**$_{\mathbf{DD}}$; **R^1** = m^{6}v$_D$v$_D$^{6}A$_m$	**ON2**$_{\mathbf{L}}$; **R^2** = v$_L$-mnm^{5}U$_m$	**ON3**$_{\mathbf{DDL}}$	34.5	6285.9	6287.0
**ON1**$_{\mathbf{DD}}$; **R^1** = m^{6}v$_D$v$_D$^{6}A$_m$	**ON2**$_{\mathbf{D}}$; **R^2** = v$_D$-mnm^{5}U$_m$	**ON3**$_{\mathbf{DDD}}$	35.3	6285.9	6286.4
**dON1**$_{\mathbf{L}}$; **R^1** = m^{6}v$_L$^{6}dA	**dON2**$_{\mathbf{L}}$; **R^2** = v$_L$-nm^{5}dT	**dON3**$_{\mathbf{LL}}$	22.1	5689.0	5689.7
**dON1**$_{\mathbf{D}}$; **R^1** = m^{6}v$_D$^{6}dA	**dON2**$_{\mathbf{L}}$; **R^2** = v$_L$-nm^{5}dT	**dON3**$_{\mathbf{DL}}$	25.1	5689.0	5689.2
**dON1**$_{\mathbf{L}}$; **R^1** = m^{6}v$_L$^{6}dA	**dON2**$_{\mathbf{D}}$; **R^2** = v$_D$-nm^{5}dT	**dON3**$_{\mathbf{LD}}$	24.3	5689.0	5689.0
**dON1**$_{\mathbf{D}}$; **R^1** = m^{6}v$_D$^{6}dA	**dON2**$_{\mathbf{D}}$; **R^2** = v$_D$-nm^{5}dT	**dON3**$_{\mathbf{DD}}$	23.6	5689.0	5688.8
**ON1**$_{\mathbf{cal}}$; **R^1** = m^{6}g^{6}A$_m$	**ON2**$_{\mathbf{cal}}$; **R^2** = mnm$_5$U$_m$	**ON3**$_{\mathbf{cal}}$	26.8	6045.7	6044.8

6. Calibration curve hairpin

Hairpin oligonucleotide **ON3$_{cal}$** or acceptor oligonucleotide **ON2$_L$** was used for the development of a HPLC calibration curve. A stock solution of **ON3$_{cal}$** or **ON2$_L$** was prepared in water (100 µM). Separate standard solutions containing 1.2; 1.0; 0.8; 0.6; 0.4; 0.2 and 0.1 nmol of **ON3$_{cal}$** or **ON2$_L$** were prepared in a final volume of 20 µL. The standard solutions were injected in an analytical HPLC equipped with a C18 column (buffer A: 0.1 M AcOH/Et_3N pH 7 in H_2O and buffer B: 0.1 M AcOH/Et_3N pH 7 in 20:80 H_2O/MeCN; Gradient: 0-40% of B in 45 min; Flow rate = 1 mL·min^{-1}). The absorbance was monitored at 260 nm and the areas of the chromatographic peaks were determined by integration of the HPL-chromatograms. The plot of the chromatographic area (a.u.) versus the amount (nmol) of the oligonucleotide followed a linear relationship.

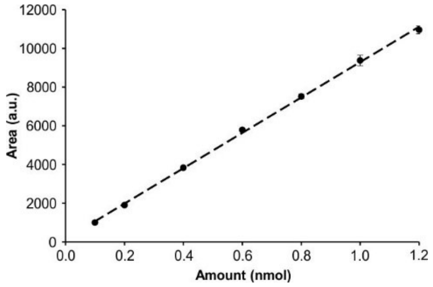

Figure S2. Chromatographic area (a.u.) *vs.* amount (nmol) of **ON3$_{cal}$**. Line shows the fit of the data to a linear regression equation. Error bars are standard deviations from two independent experiments.

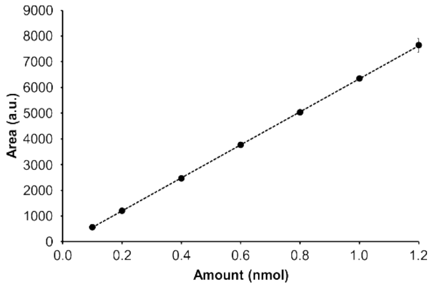

Figure S3. Chromatographic area (a.u.) *vs.* amount (nmol) of **ON2$_L$**. Line shows the fit of the data to a linear regression equation. Error bars are standard deviations from two independent experiments.

Table S6. Calibration curve (y = mx + n) obtained by HPLC analysis of **ON3$_{cal}$** and **ON2$_L$** and calculated extinction coefficients of **ON1$_{cal}$**, **ON2$_{cal}$**, **ON3$_{cal}$** and **ON2$_L$**.

**Strand**	**Slope, m (nmol^{-1})**	**Intercept, n**	**r^2**	***ε* (M^{-1}·cm^{-1})**
**ON1$_{cal}$**[a]	-	-	-	68800
**ON2$_{cal}$**[a]	-	-	-	113400
**ON3$_{cal}$**[b]	9142.5	149.4	0.99	159182
**ON2$_L$**	6428.6	- 89.8	1.0	113400

The extinction coefficients were calculated using: [a] the OligoAnalyzer Version 3.0 from Integrated DNA Technologies and [b] an hypochromicity value of *h* = 0.827 for the section of the hairpin forming base pairs as reported in the literature.[7]

7. Cleavage reaction of hairpin products

A stock solution of acetate buffer pH 4 (400 mM) and NaCl (1 M) were prepared in water. Subsequently, **ON3$_{R1R2}$** was mixed with buffer, NaCl, and water and the reaction mixture was incubated at 90°C for 20 h. The concentration of the components in the reaction mixture was: 50 µM of **ON3$_{R1R2}$**, 100 mM of buffer, 100 mM of NaCl.

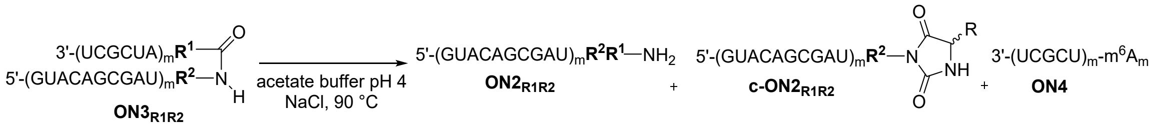

Scheme S6: Cleavage reactions of hairpin products **ON3$_{R1R2}$** to give **ON2 $_{R1R2}$**, **c-ON2 $_{R1R2}$** and **ON4.**

The hairpin cleavage reactions were only performed to isolate and characterize the products by HPLC (t_R) and mass spectrometry (*m/z*). The isolated products were used as references for the analyses of the pair-wise competitive reactions. In this respect, the reaction yields are not given.

Table S7. HPLC retention times (0-40% of B in 45 min) and MALDI-ToF mass spectrometric analysis (negative mode) of isolated cleavage products **ON2$_{R1R2}$**, **c-ON2$_{R1R2}$** and **ON4**.

Acceptor strand	t_R (min)	*m/z* calcd. for [*M*-H]$^-$	found
**ON2$_{LL}$**	25.9	3884.4	3885.3
**c-ON2$_{LL}$**	30.0	3910.3	3911.3
**ON2$_{DL}$**	28.0	3884.4	3885.6
**c-ON2$_{DL}$**	30.0	3910.3	3910.6
**ON2$_{LLL}$**	27.6	3983.4	3984.5
**c-ON2$_{LLL}$**	34.0	4009.4	4011.5
**ON2$_{LDL}$**	31.4	3983.4	3983.9
**c-ON2$_{LDL}$**	32.8	4009.4	4012.3
**ON2$_{DLL}$**	28.7	3983.4	3985.0
**c-ON2$_{DLL}$**	34.3	4009.4	4011.0
**ON2$_{DDL}$**	28.7	3983.4	3984.3
**c-ON2$_{DDL}$**	34.3	4009.4	4010.4
**ON4**	26.0	2274.3	2272.6

8. Competitive coupling and cleavage reactions (one-pot)

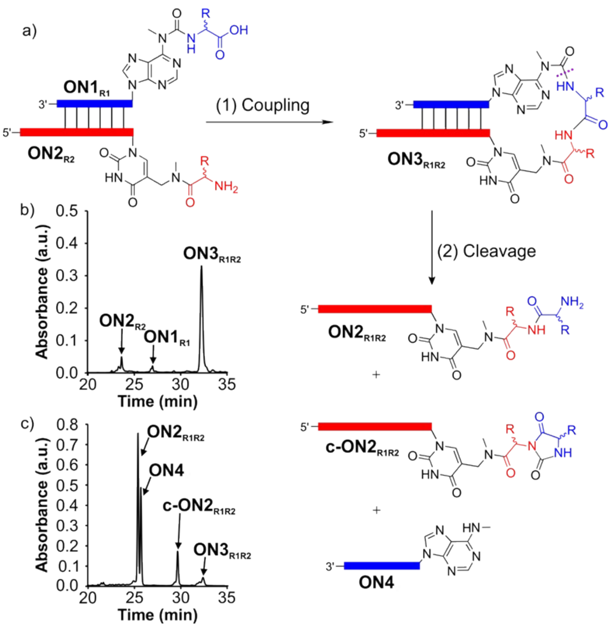

Figure S4. a) Coupling and cleavage reactions between acceptor and donor strands. Reaction conditions: (1) Coupling: EDC/Sulfo-NHS, MES buffer pH 6, NaCl, r.t., 2 h; (2) Cleavage: acetate buffer pH 4, NaCl, 90 °C, 48 h. HPL-chromatograms of the b) coupling reaction of **ON1**$_{\mathbf{R1}}$ and **ON2**$_{\mathbf{R2}}$ to yield **ON3**$_{\mathbf{R1R2}}$ and of the c) cleavage reaction of **ON3**$_{\mathbf{R1R2}}$ to yield **ON2**$_{\mathbf{R1R2}}$, **c-ON2**$_{\mathbf{R1R2}}$ and **ON4**.

$$\mathbf{ON1_L + ON1_D + ON2_L} \xrightarrow[\text{Coupling 1}]{} \mathbf{ON3_{LL}\ (+\ ON3_{DL})} \xrightarrow[\text{Cleavage 1}]{} \mathbf{ON2_{LL} + c\text{-}ON2_{LL}\ (+\ ON2_{DL} + c\text{-}ON2_{DL}) + ON4}$$

$$\xrightarrow[\text{Coupling 2}]{\mathbf{ON1_L + ON1_D}} \mathbf{ON3_{LLL}\ (+\ ON3_{DLL})} \xrightarrow[\text{Cleavage 2}]{} \mathbf{ON2_{LLL} + c\text{-}ON2_{LLL} + ON4}$$

Scheme S7. Schematic representation of the one-pot coupling/cleavage cycle.

The one-pot reactions were carried out using 20 nmol of **ON2**$_{\mathbf{L}}$ as starting material. An equimolar solution of 20 nmol **ON1**$_{\mathbf{L}}$ and **ON1**$_{\mathbf{D}}$ was prepared and the coupling reaction was carried out as described in Section 5. After each coupling step, the crude mixture was filtered using an Amicon® ultra centrifugal filter (3 kDa nominal molecular weight cut-off) to remove the unreacted activator and to exchange the buffer solution. Then, the cleavage reaction was performed as described in Section 7, although the reaction time was extended to 48 h to optimize the yield. After each step, 20 µL of the reaction mixture was analysed by HPLC.

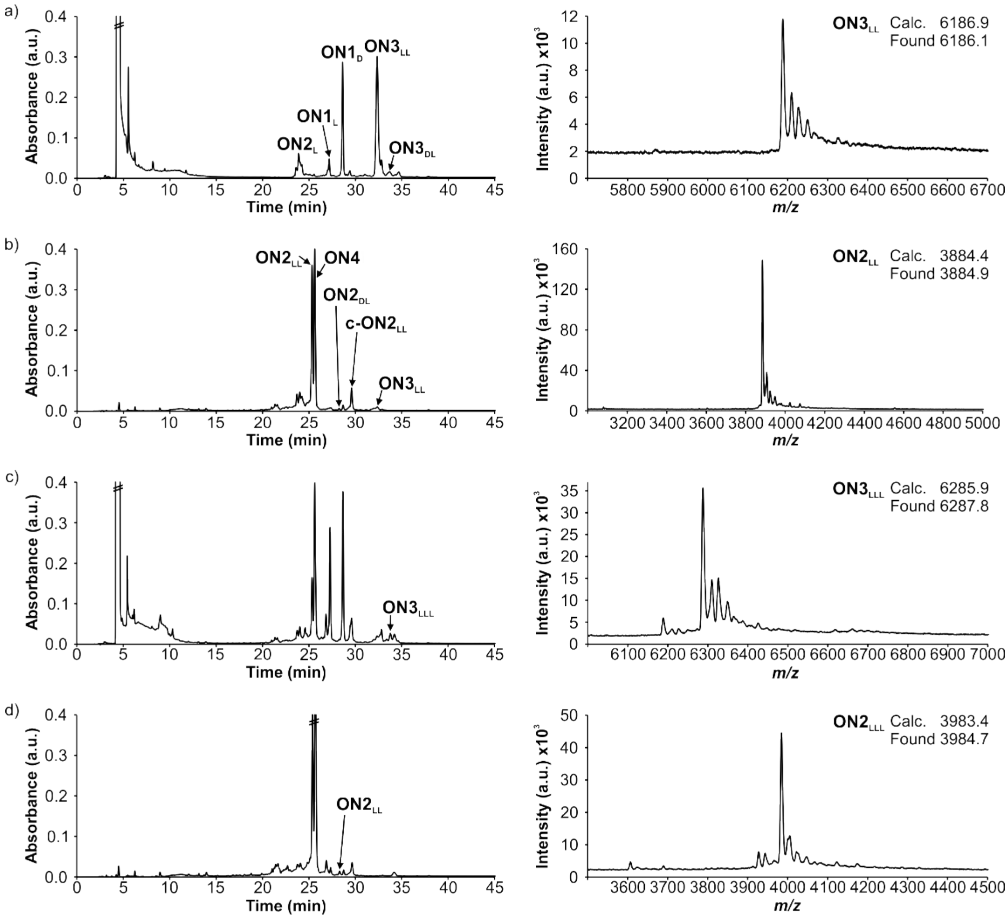

Figure S5: HPL-chromatograms of the crude reaction mixtures of the one-pot reaction and MALDI-ToF spectra of the isolated products; a) first coupling; b) first cleavage; c) second coupling and d) second cleavage.

Table S8: Results obtained in one-pot coupling and cleavage cycle.

Step	Activator	pH	T (°C)	Time (h)	Yield (%)	LL vs. DL ratio
**Coupling 1**	EDC/Sulfo-NHS	6	25	2	58%	94:6
**Cleavage 1**	-	4	90	48	42% (over two steps)	98:2
**Coupling 2**	EDC/Sulfo-NHS	6	25	2	3% (over three steps)	n.d.
**Cleavage 2**	-	4	90	48	1% (over four steps)	n.d.

9. Pair-wise competitive peptide coupling reactions between donor and acceptor oligonucleotides containing dipeptides

EDC/Sulfo-NHS as activator

The peptide coupling reactions were carried out under identical conditions to those described in Section 5 using EDC/Sulfo-NHS as activator. An equimolar solution of **ON1$_X$** and **ON1$_Y$** was prepared in water and analyzed by HPLC. The 1:1 solution mixture of donor strands, **ON1$_X$** and **ON1$_Y$**, was used to perform the coupling reactions with 1 equiv. of acceptor strand, **ON2$_L$** or **ON2$_D$**.

$$\mathbf{ON1_X + ON1_Y + ON2_L} \xrightarrow[\text{MES buffer pH 6, NaCl, r.t.}]{\text{EDC/Sulfo-NHS}} \mathbf{ON3_{XL} + ON3_{YL}}$$

$$\mathbf{ON1_X + ON1_Y + ON2_D} \xrightarrow[\text{MES buffer pH 6, NaCl, r.t.}]{\text{EDC/Sulfo-NHS}} \mathbf{ON3_{XD} + ON3_{YD}}$$

$_X$ and $_Y$ can be either $_{LL}$, $_{LD}$, $_{DL}$, or $_{DD}$, but $_X \neq _Y$

Scheme S8. Pair-wise competitive peptide coupling reactions of **ON1$_X$** and **ON1$_Y$** with **ON2$_L$** or **ON2$_D$**.

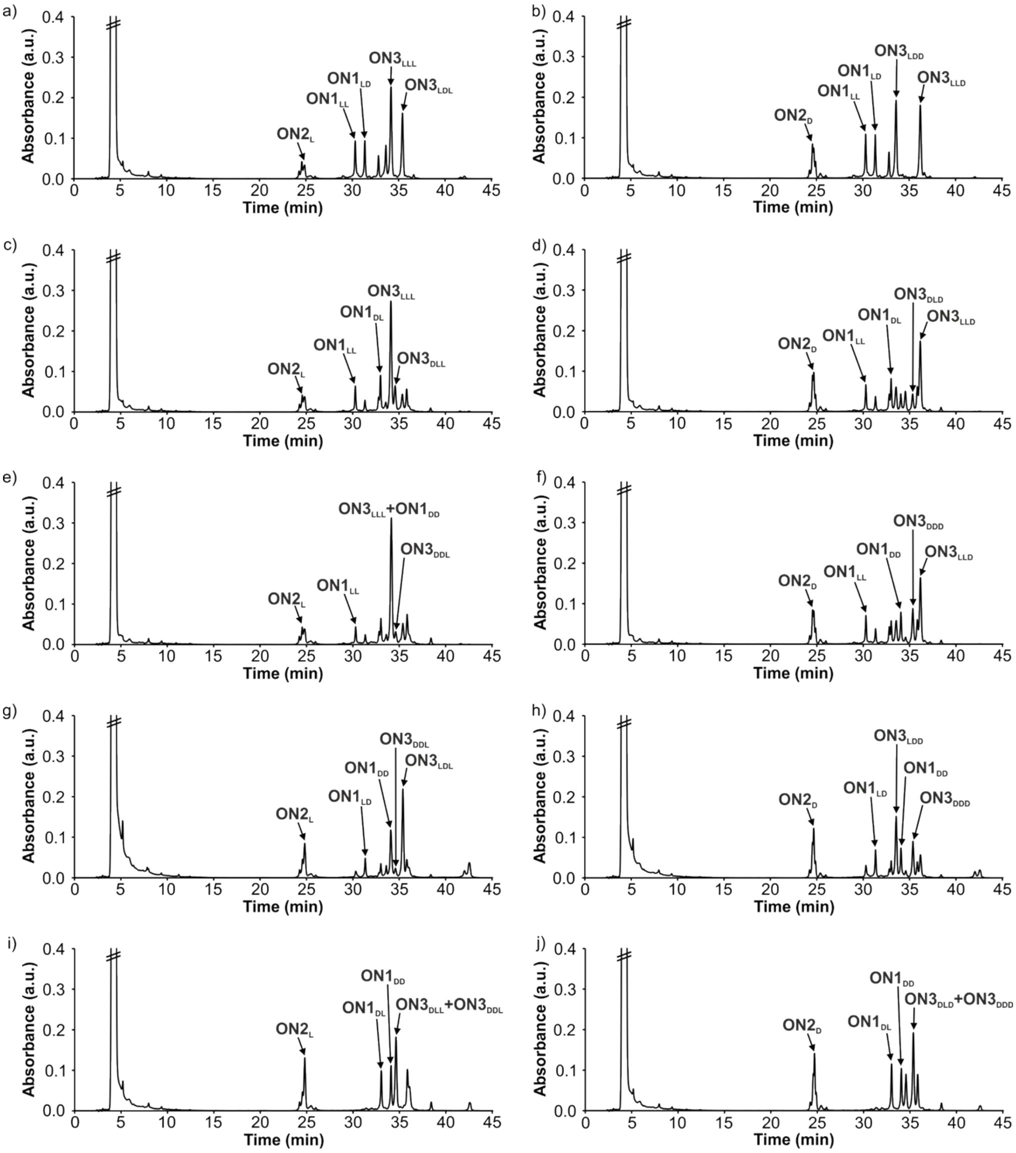

Figure S6. HPL-chromatograms of the crude reaction mixtures for the pair-wise competitive peptide coupling reactions of **ON2$_L$** with equimolar amounts of: a) **ON1$_{LL}$** and **ON1$_{LD}$**; c) **ON1$_{LL}$** and **ON1$_{DL}$**; e) **ON1$_{LL}$** and **ON1$_{DD}$**; g) **ON1$_{LD}$** and **ON1$_{DD}$** and i) **ON1$_{DL}$** and **ON1$_{DD}$** or of **ON2$_D$** with equimolar amounts of: b) **ON1$_{LL}$** and **ON1$_{LD}$**; d) **ON1$_{LL}$** and **ON1$_{DL}$**; f) **ON1$_{LL}$** and **ON1$_{DD}$**; h) **ON1$_{LD}$** and **ON1$_{DD}$** and j) **ON1$_{DL}$** and **ON1$_{DD}$**. Reactions were performed with EDC/Sulfo-NHS (2 h).

Table S9. Results obtained in the pair-wise competitive peptide coupling reactions of **ON2$_L$** and **ON2$_D$** with equimolar amounts of **ON1$_X$** and **ON1$_Y$** ($_X$ and $_Y$ can be either $_{LL}$, $_{LD}$, $_{DL}$, or $_{DD}$, but $_X \neq {}_Y$) using EDC/Sulfo-NHS as activator (average of two experiments). Errors were determined to be lower than 10%.

Acceptor strand	Donor strands	Overall yield (%)	ON3$_{XL}$/ON3$_{YL}$ ratio
	**ON1$_{LL}$** + **ON1$_{LD}$**	56	59:41
	**ON1$_{LL}$** + **ON1$_{DL}$**	48	81:19
**ON2$_L$**	**ON1$_{LL}$** + **ON1$_{DD}$**	50	n.d.
	**ON1$_{LD}$** + **ON1$_{DD}$**	48	62:38
	**ON1$_{DL}$** + **ON1$_{DD}$**	25	n.d.
**Acceptor strand**	**Donor strands**	**Overall yield (%)**	**ON3$_{XD}$/ON3$_{YD}$ ratio**
	**ON1$_{LL}$** + **ON1$_{LD}$**	50	50:50
	**ON1$_{LL}$** + **ON1$_{DL}$**	30	80:20
**ON2$_D$**	**ON1$_{LL}$** + **ON1$_{DD}$**	37	66:34
	**ON1$_{LD}$** + **ON1$_{DD}$**	34	64:36
	**ON1$_{DL}$** + **ON1$_{DD}$**	24	n.d.

n.d. = not determined (due to peak overlap)

Competitive reactions between two acceptor strands with one donor strand

The peptide coupling reactions were carried out under identical conditions to those described in Section 5 using EDC/Sulfo-NHS as activator. An equimolar solution of **ON2$_L$** and **ON2$_D$** was prepared in water and analyzed by HPLC. The 1:1 solution mixture of acceptor strands, **ON2$_L$** and **ON2$_D$**, was used to perform the coupling reactions with 1 equiv. of donor strand, **ON1$_{LL}$, ON1$_{LD}$, ON1$_{LD}$** or **ON1$_{DD}$**.

$$\mathbf{ON1_X + ON2_L + ON2_D} \xrightarrow[\text{MES buffer pH 6, NaCl, r.t.}]{\text{EDC/Sulfo-NHS}} \mathbf{ON3_{XL} + ON3_{XD}}$$

$_X$ can be either $_{LL}$, $_{LD}$, $_{DL}$, or $_{DD}$

Scheme S9. Pair-wise competitive peptide coupling reactions between **ON2$_L$** and **ON2$_D$** with **ON1$_{LL}$, ON1$_{LD}$, ON1$_{DL}$** or **ON1$_{DD}$**.

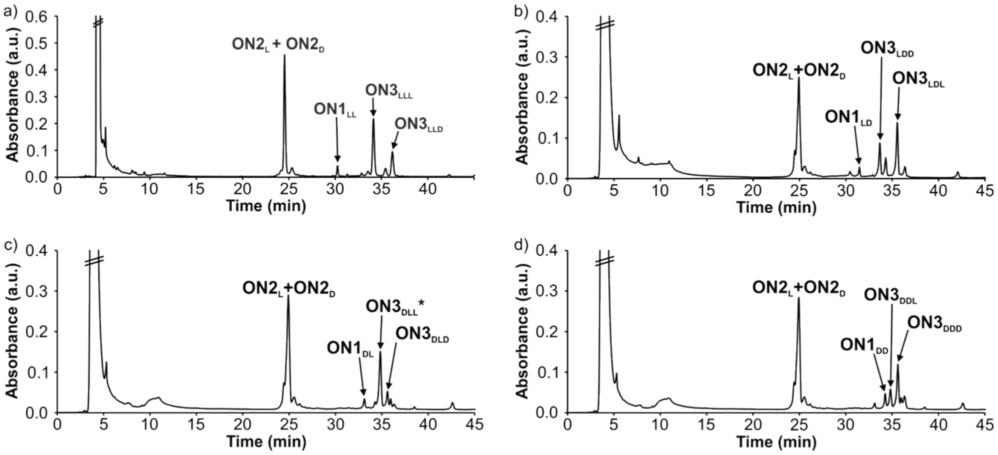

Figure S7. HPL-chromatograms of the crude reaction mixtures for the pair-wise competitive peptide coupling reactions of equimolar amounts of **ON2$_L$** and **ON2$_D$** with a) **ON1$_{LL}$**; b) **ON1$_{LD}$**; c) **ON1$_{DL}$** and d) **ON1$_{DD}$**. Reactions performed with EDC/Sulfo-NHS (2 h).

Table S10. Results obtained in the pair-wise competitive peptide coupling reactions of equimolar amounts of **ON2$_L$** and **ON2$_D$** with **ON1$_{LL}$**, **ON1$_{LD}$**, **ON1$_{LD}$** or **ON1$_{DD}$** using EDC/Sulfo-NHS as activator (average of two experiments). Errors were determined to be lower than 10%.

Acceptor strands	Donor strand	Overall yield (%)	ON3$_{XL}$/ON3$_{XD}$ ratio
	**ON1$_{LL}$**	54	66:34
	**ON1$_{LD}$**	40	62:38
**ON2$_L$ + ON2$_D$**	**ON1$_{DL}$**	n.d.	n.d.
	**ON1$_{DD}$**	27	29:71

n.d. = not determined (due to overlap with impurity)

10. Pair-wise competitive peptide coupling reactions between donor and acceptor deoxy oligonucleotides

The competitive peptide coupling reactions of an equimolar mixture of **dON1$_L$** and **dON1$_D$** with **dON2$_L$** or **dON2$_D$** were carried out under identical conditions to those described in Section 5 using EDC/Sulfo-NHS as activator.

$$\mathbf{dON1_L + dON1_D + dON2_L} \xrightarrow[\text{MES buffer pH 6, NaCl, r.t.}]{\text{EDC/Sulfo-NHS}} \mathbf{dON3_{LL} + dON3_{DL}}$$

$$\mathbf{dON1_L + dON1_D + dON2_D} \xrightarrow[\text{MES buffer pH 6, NaCl, r.t.}]{\text{EDC/Sulfo-NHS}} \mathbf{dON3_{LD} + dON3_{DD}}$$

Scheme S10. Pair-wise competitive peptide coupling reactions between **dON1$_L$** and **dON1$_D$** with **dON2$_L$** or **dON2$_D$**.

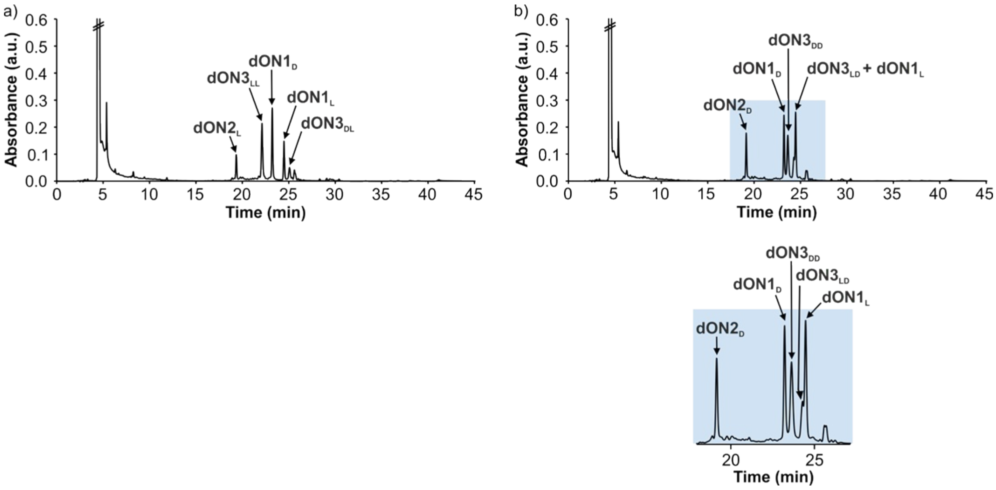

Figure S8: HPL-chromatograms of the crude reaction mixtures for the pair-wise competitive peptide coupling reactions of a) **dON2$_L$** and b) **dON2$_D$** with equimolar amounts of **dON1$_L$** and **dON1$_D$**. Reactions performed with EDC/Sulfo-NHS (2 h).

Table S11: Results obtained in the pair-wise competitive peptide coupling reactions of **dON2$_L$** and **dON2$_D$** with equimolar amounts of **dON1$_L$** and **dON1$_D$** using EDC/Sulfo-NHS as activator (average of two experiments). Errors were determined to be lower than 10%.

Acceptor strand	Donor strands	Overall yield (%)	dON3$_{LL}$/dON3$_{DL}$ ratio
**dON2$_L$**	**dON1$_L$ + dON1$_D$**	24	85:15
**Acceptor strand**	**Donor strands**	**Overall yield (%)**	**dON3$_{LD}$/dON3$_{DD}$ ratio**
**dON2$_D$**	**dON1$_L$ + dON1$_D$**	23	31:69

11. Circular dichroism measurements

The circular dichroism (CD) spectra were measured on a Jasco J-810 spectropolarimeter using 5 mm cuvettes in the spectral range from 220 nm to 310 nm and are the average of three measurements. For the experiments we prepared aqueous solutions with the following concentrations: [ON] = 5 µM, [NaCl] = 150 mM, [MES buffer] = 10 mM. The oligonucleotides were annealed by heating to 95°C for 4 min and, subsequently, by cooling down slowly to 5°C before the circular dichroism measurement.

The experiments were conducted with a) an annealed duplex of **ON1**$_{L}$ and **ON2**$_{L}$, b) with the annealed hairpin **ON3**$_{LL}$ and with c) an annealed duplex of canonical strands **ON5** and **ON6**.

ON5: 5'-(AAUCGCU)$_{m}$-3' and **ON6** 3'-(UUAGCGACAUG)$_{m}$-5' (with 2'OMe nucleotides)

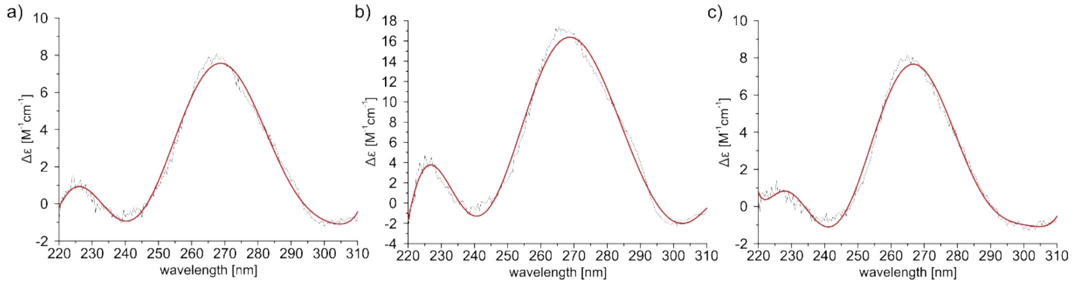

Figure S9: Circular dichroism (CD) spectra of a) annealed duplex of **ON1**$_{L}$ and **ON2**$_{L}$, b) annealed hairpin of **ON3**$_{LL}$ and c) annealed duplex of canonical strands **ON5** and **ON6**.

12. References

1. G. R. Fulmer, A. J. M. Miller, N. H. Sherden, H. E. Gottlieb, A. Nudelman, B. M. Stoltz, J. E. Bercaw and K. I. Goldberg, *Organometallics*, 2010, **29**, 2176-2179.
2. F. Müller, L. Escobar, F. Xu, E. Węgrzyn, M. Nainytė, T. Amatov, C. Y. Chan, A. Pichler and T. Carell, *Nature*, 2022, **605**, 279-284.
3. M. Nainytė, F. Müller, G. Ganazzoli, C.-Y. Chan, A. Crisp, D. Globisch and T. Carell, *Chem. Eur. J.*, 2020, **26**, 14856-14860.
4. F. Himmelsbach, B. S. Schulz, T. Trichtinger, R. Charubala and W. Pfleiderer, *Tetrahedron*, 1984, **40**, 59-72.
5. F. Ferreira and F. Morvan, *Nucleosides, Nucleotides Nucleic Acids*, 2005, **24**, 1009-1013.
6. A. V. Tataurov, Y. You and R. Owczarzy, *Biophys. Chem.*, 2008, **133**, 66-70.
